# A Compartmentalized Mathematical Model of the β_1_-Adrenergic Signaling System in Mouse Ventricular Myocytes

**DOI:** 10.1371/journal.pone.0089113

**Published:** 2014-02-21

**Authors:** Vladimir E. Bondarenko

**Affiliations:** Department of Mathematics and Statistics and Neuroscience Institute, Georgia State University, Atlanta, Georgia, United States of America; Loyola University Chicago, United States of America

## Abstract

The β_1_-adrenergic signaling system plays an important role in the functioning of cardiac cells. Experimental data shows that the activation of this system produces inotropy, lusitropy, and chronotropy in the heart, such as increased magnitude and relaxation rates of [Ca^2+^]_i_ transients and contraction force, and increased heart rhythm. However, excessive stimulation of β_1_-adrenergic receptors leads to heart dysfunction and heart failure. In this paper, a comprehensive, experimentally based mathematical model of the β_1_-adrenergic signaling system for mouse ventricular myocytes is developed, which includes major subcellular functional compartments (caveolae, extracaveolae, and cytosol). The model describes biochemical reactions that occur during stimulation of β_1_-adrenoceptors, changes in ionic currents, and modifications of Ca^2+^ handling system. Simulations describe the dynamics of major signaling molecules, such as cyclic AMP and protein kinase A, in different subcellular compartments; the effects of inhibition of phosphodiesterases on cAMP production; kinetics and magnitudes of phosphorylation of ion channels, transporters, and Ca^2+^ handling proteins; modifications of action potential shape and duration; magnitudes and relaxation rates of [Ca^2+^]_i_ transients; changes in intracellular and transmembrane Ca^2+^ fluxes; and [Na^+^]_i_ fluxes and dynamics. The model elucidates complex interactions of ionic currents upon activation of β_1_-adrenoceptors at different stimulation frequencies, which ultimately lead to a relatively modest increase in action potential duration and significant increase in [Ca^2+^]_i_ transients. In particular, the model includes two subpopulations of the L-type Ca^2+^ channels, in caveolae and extracaveolae compartments, and their effects on the action potential and [Ca^2+^]_i_ transients are investigated. The presented model can be used by researchers for the interpretation of experimental data and for the developments of mathematical models for other species or for pathological conditions.

## Introduction

Cardiac cells belong to a wide class of excitable cells which include electrical activity, Ca^2+^ dynamics, and protein signaling networks. While early experimental studies of cardiac cells are predominantly devoted to their electrical activity, and later to Ca^2+^ dynamics, more recent studies involve investigations of protein signaling systems, which modulate both action potentials and intracellular Ca^2+^ transients [1], [2]. On the tissue and whole heart levels, the activation of such signaling systems either promotes or suppresses pro-arrhythmic behavior. In addition, in diseased hearts, protein signaling networks become modified and do not properly regulate the electrical activity or Ca^2+^ handling system [3]. As a result, the studies of major signaling protein networks in cardiac cells identified new potential therapeutic targets for treatment of heart diseases; some of the targets include signaling proteins involved in β_1_-adrenergic signaling system [4], [5], [6], [7].

Mathematical modeling of protein signaling networks is a supplementary tool for understanding their functions in the heart. Recently, particular attention has been paid to the development of comprehensive models for β_1_-adrenergic system in ventricular myocytes of different species [8], [9], [10], [11], [12]. The first model developed by Saucerman et al. [10] for rat ventricular myocytes set high standards for simulation of the β_1_-adrenergic signaling system. The model included biochemical and electrophysiological parts with two major protein kinase A targets, phospholamban and the L-type Ca^2+^ channel, and consisted of one cytosolic compartment (single-compartment model). Later, a similar model was developed for rabbit ventricular myocytes and included several new PKA targets: ryanodine receptors, troponin I, and slow delayed rectifier K^+^ current, I_Ks_ [11]. The model was further extended to simulate the effects of the β_1_-adrenergic signaling system in mouse ventricular myocytes, predominantly on Ca^2+^ dynamics [12]. Finally, a model of β_1_-adrenergic signaling system in guinea pig ventricular myocytes [9] was developed based on the model of Saucerman et al. [10], which is devoted to the analysis of the changes in action potential, intracellular [Ca^2+^]_i_ transients, and ionic fluxes upon stimulation of β_1_-adrenoceptors with agonist isoproterenol.

Simultaneously, multi-compartmental models of protein signaling networks, including the β_1_-adrenergic signaling system, were developed [8], [13], [14]. The compartmentalized models of Iancu et al. [13], [14] included only the biochemical part of β_1_-adrenergic and M_2_-muscarinic signaling systems and described the dynamics of cAMP and PKA in different subcellular compartments (caveolae, extracaveolae, and cytosol). The only compartmentalized model of cardiac protein signaling network, which includes both biochemical and electrophysiological parts, β_1_- and β_2_-adrenergic and CaMKII-mediated signaling systems, was developed recently for canine ventricular myocytes by Heijman et al. [8]. The model was extensively verified by experimental data and reproduced major features of stimulation of the three signaling systems.

Compartmentalization of the signaling systems in cardiac cells is an important property. This property allows for regulation of multiple cellular functions, such as electrical activity, Ca^2+^ dynamics, and cellular contraction (for examples see reviews [1], [15], [16], [17], [18]). The experimental data demonstrates differential localization of the components of the Ca^2+^-mediated, α- and β-adrenergic signaling systems [19], [20], [21], [22]. In cardiac myocytes, β_1_-adrenergic receptors are mostly localized in membrane compartments that lack caveolin-3, while β_2_-adrenergic receptors are mostly found in caveolin-3-rich domains [20]. Investigations of the physiological role of the β-receptors have shown their differing effects in the development of disease states: excessive activation of β_1_-adrenergic signaling led to cardiac hypertrophy and heart failure [23], while moderately increased stimulation of β_2_-adrenergic signaling was cardioprotective [24]. In addition, β_1_- and β_2_-adrenergic receptors modulate differently cardiac ionic currents and contraction proteins, which are also localized in different cellular compartments [15], [16]. In the β_1_-adrenergic signaling system alone, which is the major topic of this paper, multiple signaling molecules are also distributed among the major cellular compartments related to caveolin-3, non-caveolae cellular membrane, or cytosol, and these molecules are differentially modulated upon activation of β_1_-receptors. In particular, the recent discovery of the two subpopulations of the L-type Ca^2+^ channels, the major players in cardiac excitation-contraction coupling, which are localized in caveolin-3-rich and non-caveolae compartments and play different physiological roles, requires more comprehensive, compartmentalized models of cardiac cells [22].

In this paper, we developed a new compartmentalized model for the β_1_-adrenergic signaling system in mouse ventricular myocytes. The model is based on our previously published models for action potential and Ca^2+^ dynamics in mouse ventricular myocytes [25], [26]. The new model includes both biochemical and electrophysiological parts, as well as compartmentalization of the β_1_-adrenergic signaling system, which includes three major compartments: caveolae, extracaveolae, and cytosol. Both biochemical and electrophysiological parts are verified by extensive experimental data, primarily obtained from the rodent cardiac cells. Activation of the major proteins in the signaling system, such as adenylyl cyclases and phosphodiesterases, is compared directly to the data from the mouse ventricular myocytes in absolute magnitudes. The model successfully reproduced existing experimental data on cAMP dynamics, activation of adenylyl cyclases and phosphodiesterases, protein kinase A and phosphorylation of its targets, and the effects of phosphodiesterases inhibition on cAMP transients. Simulations also reproduced data obtained from voltage-clamp protocols for major repolarization currents of mouse ventricular myocytes. The model is able to simulate action potential shape and duration upon stimulation of β_1_-adrenergic receptors (β_1_-ARs). The model elucidated the mechanism of relatively moderate AP prolongation, significant increase in intracellular [Ca^2+^]_i_ transients, modification of the intracellular and transmembrane Ca^2+^ fluxes, and [Na^+^]_i_ fluxes and dynamics. The model includes two pools of the L-type Ca^2+^ channels, one in the caveolae and the other in the extracaveolae compartments. The simulations demonstrated their different modulations upon stimulation of β_1_-ARs and their different effects on the action potentials and [Ca^2+^]_i_ transients. The model simulated frequency dependences of [Ca^2+^]_i_ transient decays for control conditions and after stimulation of β_1_-ARs, and made testable predictions for the frequency dependences of [Ca^2+^]_i_ transient amplitudes, AP amplitudes and durations. The simulation results are compared to the results obtained from other models of β_1_-adrenergic signaling system in other species, and the model limitations are discussed.

## Methods

**Figure 1:**
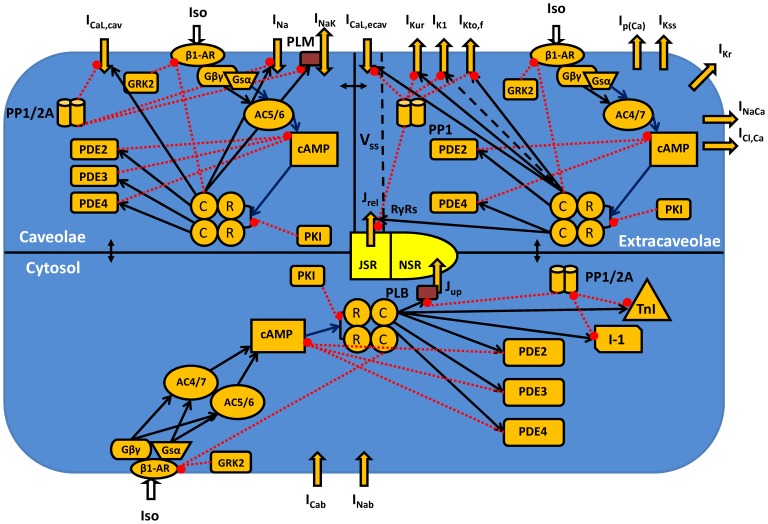
Schematic representation of the β_1_-adrenergic signaling system in mouse ventricular myocytes. Transmembrane currents are the fast Na^+^ current (I_Na_), the two components of the L-type Ca^2+^ current in caveolae and extracaveolae compartments (I_CaL,cav_ and I_CaL,ecav_, respectively), the sarcolemmal Ca^2+^ pump (I_p(Ca)_), the Na^+^/Ca^2+^ exchanger (I_NaCa_), the rapidly recovering transient outward K^+^ current (I_Kto,f_), the rapid delayed rectifier K^+^ current (I_Kr_), the ultrarapidly activating delayed rectifier K^+^ current (I_Kur_), the noninactivating steady-state voltage activated K^+^ current (I_Kss_), the time-independent K^+^ current (I_K1_), the Na^+^/K^+^ pump (I_NaK_, is regulated by phospholemman, PLM), the Ca^2+^ and Na^+^ background currents (I_Cab_ and I_Nab_). The Ca^2+^ fluxes are uptake of Ca^2+^ from the cytosol to the network sarcoplasmic reticulum (NSR) (J_up_) by the SERCA pump and Ca^2+^ release from the junctional sarcoplasmic reticulum (JSR) (J_rel_) through the ryanodine receptors (RyRs). There are three intracellular compartments in the β_1_-adrenergic signaling system: caveolae, extracaveolae, and cytosol. The subspace volume (V_ss_) is located in the extracaveolae domain. Components of the β_1_-adrenergic signaling system are the β_1_-adrenergic receptors (β1-AR), the α-subunit of stimulatory G-protein (G_sα_), the βγ-subunit of stimulatory G-protein (G_βγ_), the adenylyl cyclases of type 5/6 or 4/7 (AC5/6 or AC4/7, respectively), the phosphodiesterases of type 2, 3, or 4 (PDE2, PDE3, or PDE4, respectively), the cyclic AMP (cAMP), regulatory (R) and catalytic (C) subunits of protein kinase A holoenzyme, the protein kinase A inhibitor (PKI), the G-protein-coupled receptor kinase of type 2 (GRK2), the protein phosphatases of type 1 or 2A (PP1 or PP2A, respectively), the inhibitor-1 (I-1). The cytosolic proteins which are the substrates of the β_1_-adrenergic signaling system are the phospholamban (PLB) and troponin I (TnI). Stimulatory links are shown by black arrows and inhibitory links are shown by red dashed lines with balls. [Ca^2+^]_i_, [Na^+^]_i_, and [K^+^]_i_ are the intracellular Ca^2+^, Na^+^, and K^+^ concentrations in the caveolae, extracaveolae, and cytosol; [Ca^2+^]_o_, [Na^+^]_o_, and [K^+^]_o_ are the extracellular Ca^2+^, Na^+^, and K^+^ concentrations.

A mathematical model for the β_1_-adrenergic signaling system in mouse ventricular myocytes is a natural extension of the previously published model for action potential and Ca^2+^ dynamics in mouse ventricular myocytes [25], with model improvements from [26] (Fig. 1). We incorporated a β_1_-adrenergic signaling pathway in our model of electrical activity and Ca^2+^ handling with modifications [25], [26] (see Appendix S1).

Our model cell consists of several compartments (Fig. 1; Appendix S1). For the β_1_-adrenergic signaling system we consider three major *functional* compartments: caveolae (cav), extracaveolae (ecav), and cytosol (cyt) [8], [13]. The caveolae compartment is a submembrane compartment associated with the protein caveolin-3. The extracaveolae compartment is also a submembrane compartment associated with cholesterol-rich lipid rafts, but it does not include caveolin-3. Both caveolae and extracaveolae compartments together represent the particulate fraction. The cytosolic compartment (the soluble fraction) represents the rest of the cell volume, excluding mitochondria. As the dynamics of Ca^2+^ concentration does not directly depend on the β_1_-adrenergic signaling system, we use the same compartmentalization of Ca^2+^ handling as in Bondarenko et al. [25]. The subspace volume (V_ss_) of the Ca^2+^ handling system is completely located in the extracaveolae compartment of the β_1_-adrenergic signaling system.

Total protein concentrations and their activities are normalized to the cell volume as in [8]. In most cases, we used Table 9 from [27] to convert protein concentrations from pmol/mg cell protein to the concentrations in µM. The localization of different signaling proteins and protein kinase A substrates in subcellular compartments can be found in Fig. 1 and Appendix S1 and will be described below in the corresponding chapters. The experimental data in the Methods chapter are used for constraining the model; the experimental data in the Results chapter are used for testing the developed model. The latter experimental data were not used for constraining the model.

In all compartments, the β_1_-adrenergic signaling system is activated by agonist (isoproterenol) (Fig. 1). Stimulation of β_1_-ARs leads to activation of the stimulatory G protein, G_s_, which dissociates into G_sα_ and G_sβγ_ subunits. Both subunits activate adenylyl cyclases (AC5/6 or AC4/7, depending on the cellular compartment), which produce cyclic AMP. cAMP is degraded by phosphodiesterases, three isoforms of which, PDE2, PDE3, and PDE4, are included in our model. Balanced activities of ACs and PDEs establish steady-state levels of cAMP in different compartments. cAMP further activates protein kinase A holoenzyme, which consists of two regulatory and two catalytic subunits. Binding four cAMP molecules to PKA holoenzyme causes dissociation of two catalytic subunits that phosphorylate target proteins, among them are PDE3 and PDE4. β_1_-ARs are phosphorylated by PKA, as well as by G protein coupled receptor kinase of type 2 (GRK2). PKA is also regulated by heat-stable protein kinase inhibitor (PKI). Intracellular proteins are dephosphorylated by two types of phosphatases, protein phosphatase 1 and 2A. PKA target proteins are located in different compartments. In our model, 20% of the L-type Ca^2+^ channels (the L-type Ca^2+^ current, I_CaL_), the fast Na^+^ current, I_Na_, and the phospholemman, which regulates the Na^+^-K^+^ pump, I_NaK_, are localized in the caveolae compartment; 80% of the L-type Ca^2+^ channels, the ryanodine receptors, RyRs, the ultra-rapidly activating delayed rectifier K^+^ current, I_Kur_, the rapidly inactivating transient outward K^+^ current, I_Kto,f_, and the time-independent K^+^ current, I_K1_, are localized in the extracaveolae compartment; and phospholamban and troponin I are localized in the cytosolic compartment (more details are shown below).

### Model Development: Biochemical Part

#### β_1_-adrenergic receptor module

According to the experimental findings [19], [20], [28], the vast majority of β_1_-adrenergic receptors are located in non-caveolae fractions. The estimated total concentration of β_1_-ARs in mouse ventricular myocytes is 0.0103 µM [29]. In our model, we distribute the β_1_-ARs almost evenly between the extracaveolae and cytosolic compartments, with only 1% located in the caveolae compartment (see Appendix S1). Such distribution of β_1_-ARs allowed us to obtain in the model approximately equal cAMP transients in the caveolae and extracaveolae compartments, which is in line with the measurements of local cAMP concentrations in similar compartments in rat and mouse ventricular myocytes [30]. In the β_1_-adrenergic receptor module, we separate relatively fast biochemical reactions (ligand-receptor and G-protein-receptor interactions, with time scales of tens milliseconds [31]), which are described by algebraic equations in steady-state approximation, and slower reactions (G-protein activation (hundreds milliseconds [31]), PKA- and GRK2-mediated phosphorylation (hundreds seconds [31]; GRK2, G-protein-coupled receptor kinase of type 2)), which are described by ordinary differential equations (see Appendix S1).

In order to derive algebraic equations which describe ligand-receptor and G-protein-receptor interactions in the caveolae compartment (equations (A.7)-(A.11) in the Appendix S1), we consider mass conservation laws for non-phosphorylated β_1_-adrenergic receptors and G-proteins in that compartment:
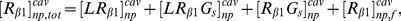
(1)

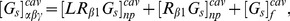
(2)where 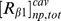 is the total concentration of non-phosphorylated (*np*) β_1_-ARs in the caveolae compartment, 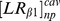 is the concentration of β_1_-ARs with bound ligand *L* (concentration [*L*]), 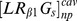 is the concentration of β_1_-ARs with bound ligand *L* and stimulatory G-protein *G*_s_, 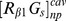 is the concentration of β_1_-ARs with bound *G*_s_, 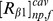 is the concentration of free β_1_-ARs, 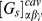 is the total concentration of the stimulatory G-protein *G*_s_, and 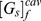 is the concentration of free *G*_s_.

Concentrations of complexes 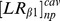, 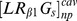, and 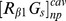
can be obtained from the steady-state approximation for corresponding biochemical reactions, provided that the related dissociation constants are known (equations (A.12)-(A.14) in the Appendix S1). The steady-state approximation for a chemical reaction describes the formation of complex AB from the substances A and B: A+B ↔ AB. For this reaction, k^+^ and k^−^ are the forward and backward rates, respectively, and K is the dissociation constant (K = k^−^/k^+^). The complex formation is described by the differential equation d[AB]/dt = k^+^[A]·[B] − k^−^[AB], where [A], [B], and [AB] are the concentrations of the substances A, B, and complex AB, respectively. In steady state, d[AB]/dt = 0, therefore, k^+^[A]·[B] − k^−^[AB] = 0. From the last equation, we can determine the concentration of complex AB in the steady-state approximation: [AB] = [A]·[B]/(k^−^/k^+^) = [A]·[B]/K.

Substitution of the equation for 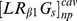, and 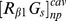 into equation (1) and solution with respect to 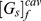 yields equation (A.11). Further substitution of the expressions for 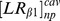, 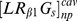, 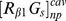, and 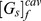 into equation (2) results in equation (A.10). The complete system of algebraic equations for ligand-receptor and G-protein-receptor interactions for the caveolae, extracaveolae, and cytosolic compartments is given in Appendix S1.

Activation of G_s_-proteins, with dissociation of 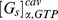 and 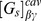-subunits, occurs through a relatively fast process with 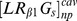 complex or a relatively slow process with 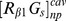 complex, and 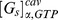 is hydrolyzed further to 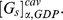 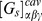 is formed back through re-association of 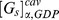 and 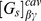 (equations (A.17)-(A.19) in Appendix S1) [8], [10].

**Figure 2:**
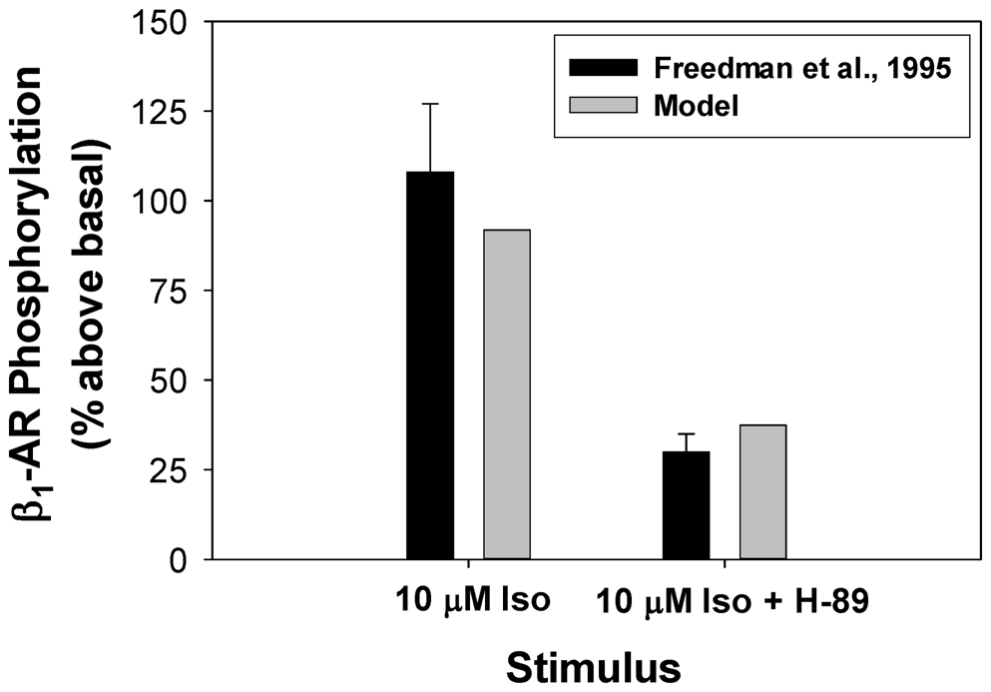
β_1_-adrenoceptors phosphorylation. β_1_-adrenoceptors phosphorylation above basal level after 15-minute application of 10 µM isoproterenol or 10 µM isoproterenol+PKA inhibitor H-89. Experimental data from Freedman et al. [32] are shown with black bars with errors, simulation data are shown with gray bars. Effect of H-89 was simulated by setting [*PKA*]_tot_ = 0 µM.

Phosphorylation (desensitization) of β_1_-ARs occurs by both protein kinase A and G-protein-coupled receptor kinase of type 2, GRK2 [8], [10] (equations (A.15) and (A.16) for the caveolae compartment). PKA phosphorylates β_1_-ARs both in ligand- and G-protein-bound states and unbound states, while GRK2 phosphorylation affects only ligand-bound states [8], [10]. According to the experimental finding [32], application of 10 µM of β_1_-ARs agonist isoproterenol increased the phosphorylation level of β_1_-ARs by about 108% (Fig. 2), which is reproduced by our model. Moreover, about 30% of phosphorylation of β_1_-ARs occurs by GRK2, as it is found from the suppression of PKA by H-89 (Fig. 2). Our model is also able to reproduce this effect.

#### Adenylyl cyclase module

In a β_1_-adrenergic pathway, adenylyl cyclases are responsible for synthesis of cAMP from ATP. Our model includes four major types of adenylyl cyclases (AC) found in mouse ventricular myocytes, AC4, AC5, AC6, and AC7 [33], [34]. Two of them, AC5 and AC6, are located in caveolae and have similar properties (we denote them as AC5/6) [35]. Two others, AC4 and AC7, also have similar properties: they do not co-immunoprecipitate with caveolin-3 and they are excluded from caveolae (we denote them as AC4/7) [13], [28], [36].

We simulated activation of adenylyl cyclases by the α-subunit of G-protein, G_sα_, [8], [13] (see Appendix S1). In addition, we considered the stimulation of adenylyl cyclases AC5/6 and AC4/7 by βγ-subunit, G_sβγ_, according to the experimental data [37], [38], [39], [40]. The total amount of adenylyl cyclases in a cardiac cell is estimated as 0.02622 µM [41], with 74% of AC5/6 type [8].

**Figure 3:**
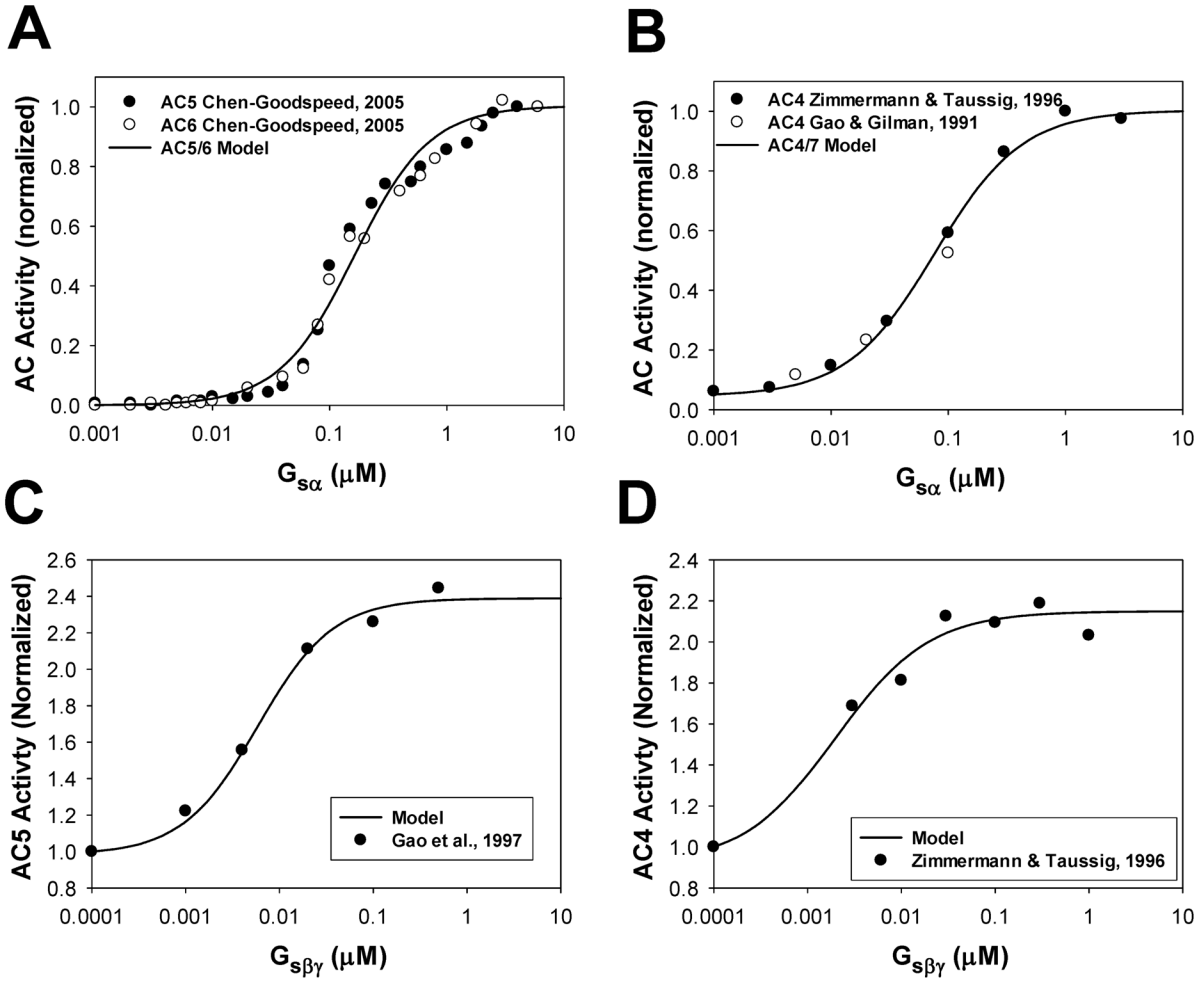
Normalized activity of adenylyl cyclases as functions of G_sα_ and G_sβγ_. **Panel A**: Experimental normalized activity of AC5 (filled circles) and AC6 (unfilled circles) as functions of G_sα_ [37]. Simulated data for normalized activity of AC5/6 is shown by a solid line. **Panel B**: Experimental normalized activity of AC4 (filled circles from [40] and unfilled circles from [38]) as functions of G_sα_. Simulated data for normalized activity of AC4/7 is shown by a solid line. **Panel C**: Experimental normalized activity of AC5 (filled circles) [39] as functions of G_sβγ_. Simulated data for normalized activity of AC5/6 is shown by a solid line. **Panel D**: Experimental normalized activity of AC4 (filled circles) [40] as functions of G_sβγ_. Simulated data for normalized activity of AC4/7 is shown by a solid line.

Figure 3A shows experimental data on activation of AC5 and AC6 by G_sα_ [37] and corresponding simulation data using our model for AC5/6 activation. In Fig. 3B, simulation data on activation of AC4/7 by G_sα_ is compared to the experimental data for AC4 [38], [40]. Figures 3C and 3D compare the experimental data from [39], [40] to our simulations for the dependence of AC5/6 and AC4/7 activities on G_sβγ_. Both figures show good agreement between the experimental and simulated results.

**Figure 4:**
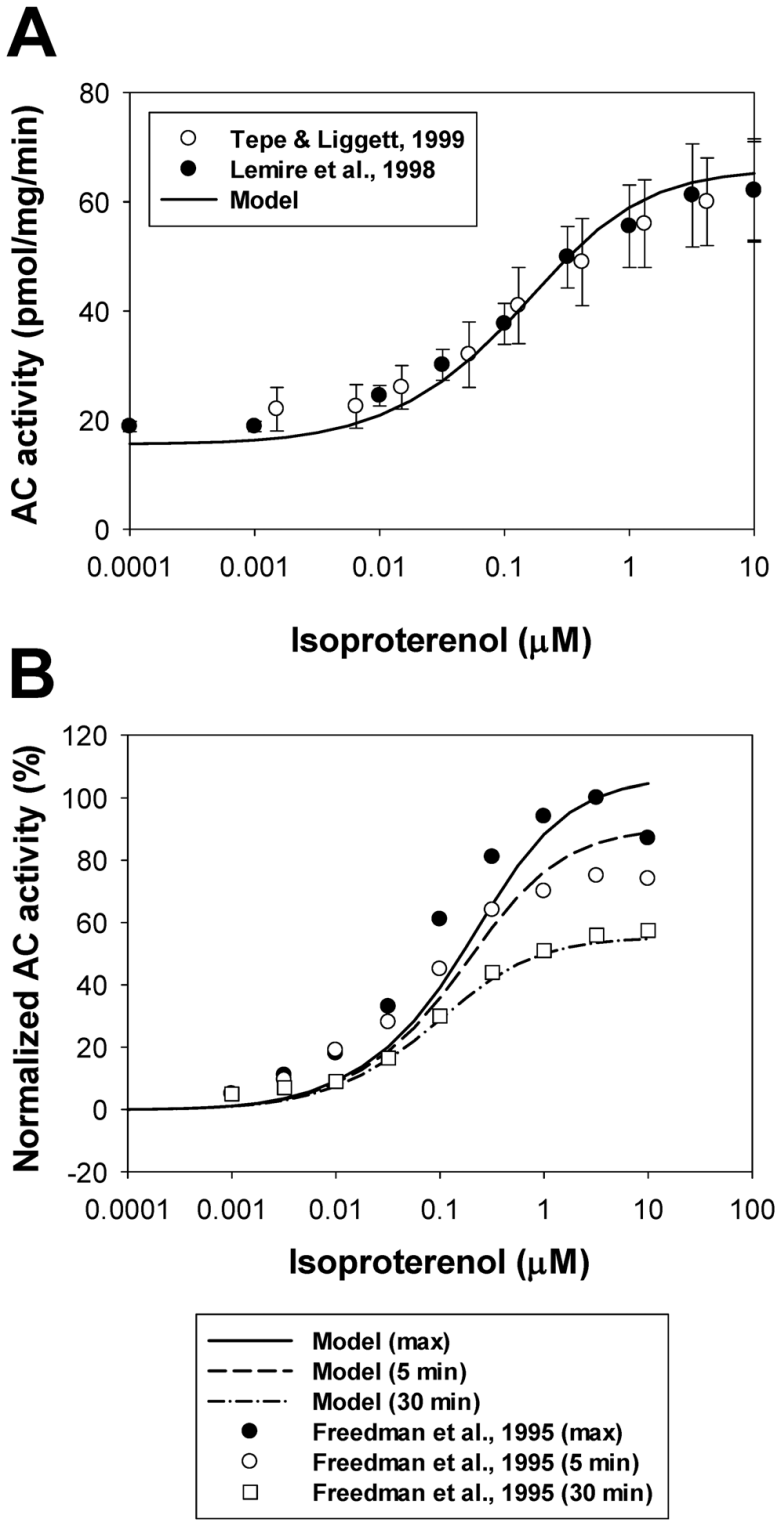
Adenylyl cyclase activity as a function of isoproterenol. **Panel A**: Experimental data on AC activity (in pmol/mg/min) in mouse hearts and ventricular myocytes obtained after 10-minutes exposure to isoproterenol are shown by unfilled circles [42] and filled circles [43]. The solid line shows corresponding simulated AC activities at different concentrations of isoproterenol. **Panel B**: Desensitization of β_1_-ARs. Increase in adenylyl cyclase activities above basal level (in %) are measured at maximum (from 50^th^ to 75^th^ seconds, control, filled circles) and at two time moments (5 min and 30 min, unfilled circles and unfilled squares, respectively) after exposure to different concentrations of isoproterenol [32]. Corresponding simulated data for the maximum, 5-minute, and 30-minute delays are shown by solid, dashed, and dash-dotted lines, respectively.

We also simulated the effects of different concentrations of β_1_-adrenoceptor agonist isoproterenol on adenylyl cyclase activity in mouse ventricular myocytes (Fig. 4A). Experimental data on total AC activity in mouse ventricles and cardiac cells as a function of isoproterenol concentration after 10-min exposures are shown by unfilled [42] and filled circles [43] with error bars. Simulation data on AC activity at the 10^th^ minute after the exposure to different concentrations of isoproterenol are shown by a solid line. As seen in Fig. 4A, our model was able to reproduce absolute values of the total cellular adenylyl cyclase activity as a function of isoproterenol.

Adenylyl cyclase activity can also be used as an indicator of desensitization of β_1_-ARs. Experimental data on AC activity as a function of isoproterenol were obtained by Freedman et al. [32] at three time moments after exposure to an agonist (at the maximum activity (from 50^th^ to 75^th^ seconds, depending on isoproterenol concentration), 5^th^ min, and 30^th^ min). It is shown that the AC activity decreases in time, reflecting β_1_-ARs desensitization (phosphorylation by PKA and GRK2) (symbols in Fig. 4B). Our model satisfactory reproduced this phenomenon. Simulation data also demonstrates the decrease in AC activity as a function of time at different concentrations of isoproterenol (solid, dashed, and dash-dotted lines in Fig. 4B).

#### Phosphodiesterase module

Phosphodiesterases in the β_1_-adrenergic signaling system degrades cAMP into inert molecule 5′-AMP. We included in our model three major types of phosphodiesterases (PDE2, PDE3, and PDE4) found in mouse ventricular myocytes [44]. While a significant amount of PDE1 was found in mouse ventricles, the study of Bode et al. [45] shows that this type of PDE is predominantly located in non-myocyte cells. As in previous models [8], [13], we put PDE2, PDE3, and PDE4 into the caveolae and cytosolic compartments, and PDE2 and PDE4 into the extracaveolae compartment. Such distribution fits available experimental data on their localization [46], [47]. For subcellular distribution of the PDE isoforms we used experimental data obtained by Mongillo et al. [48] for rat ventricular myocytes, and the parameters were adjusted to fit experimental data on PDE2, PDE3, and PDE4 activities in mouse hearts [44], [49].

PDE2, PDE3, and PDE4 are activated by cAMP molecules, but with different affinities (see Appendix S1). In addition, phosphorylation of PDE3 and PDE4 increases their activities by several folds. These processes are simulated by ordinary differential equations derived for three subcellular compartments (caveolae, extracaveolae, and cytosol, see Appendix S1). Our model also tested the effects of non-specific PDE inhibitor 3-isobutyl-1-methylxanthine (IBMX).

**Figure 5:**
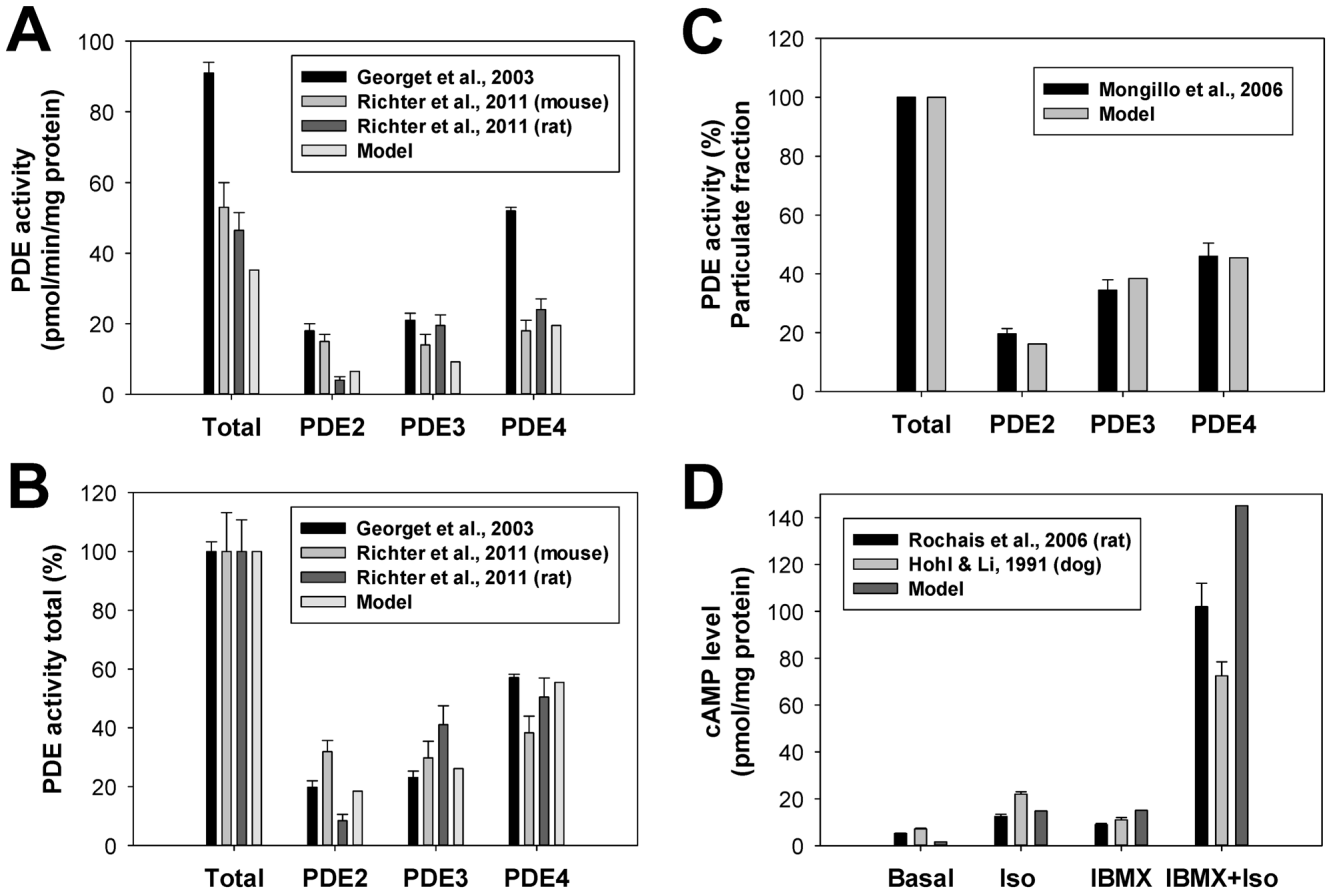
The effects of β_1_-adrenoceptor stimulation on PDE activities. **Panel A**: Absolute total PDE activities and the activities of PDE2, PDE3, and PDE4 obtained experimentally from the mouse and rat hearts (in pmol/min/mg protein, bars with errors [44], [49]) and corresponding simulation data (bars without errors). **Panel B**: Fractional activities of PDE2, PDE3, and PDE4 obtained experimentally from the mouse and rat hearts (in %, bars with errors [44], [49]) and corresponding simulation data (bars without errors). **Panel C**: Partial activities of PDE2, PDE3, and PDE4 obtained experimentally from the particulate fraction of the rat hearts (in %, bars with errors [48]) and corresponding simulation data (bars without errors). In the experiments and simulations, PDE activities are obtained at fixed cAMP concentrations of 1 µM. **Panel D**: The effects of PDE inhibitor IBMX on cAMP levels in ventricular myocytes. Experimental data for rat [147] and canine [148] ventricular myocytes are shown by bars with errors, simulation data are shown by bars without errors. In the experiments with rat ventricular myocytes measurements were performed after 3-minute stimulations (5 µM isoproterenol, or 100 µM IBMX, or both) [147]. In the experiments with canine ventricular myocytes measurements were performed after 5-minute stimulations (10 µM isoproterenol, or 10 µM IBMX, or both) [148]. Simulated cAMP level is determined at the 3^rd^ minute upon application of 5 µM isoproterenol, or 100 µM IBMX, or both.

Figure 5A shows experimental and simulated absolute activities of PDE2, PDE3, and PDE4 in mouse and rat hearts. There are some differences in the magnitudes of experimental contributions of different PDE isoforms in total cellular PDE activity obtained by different research groups and between species. However, these differences are diminished in Fig. 5B for fractional contributions of different PDEs and between species. Our model successfully reproduced both absolute PDE activities and the fractional contributions of PDE2, PDE3, and PDE4 to the total cellular PDE activity. In addition to the cellular activity, we were able to reproduce the partial contributions of different PDE isoforms into the total PDE activity of particulate fraction (Fig. 5C). In this figure, we used experimental data of Mongillo et al. [48] obtained from rat cardiomyocytes.

The effects of the PDE inhibitor IBMX on cAMP production in ventricular myocytes can be estimated by the levels of cAMP at different experimental conditions. Figure 5D shows experimental cAMP levels in ventricular myocytes from the rat and canine hearts at the resting state after application of isoproterenol or IBMX separately and at simultaneous application of IBMX and isoproterenol. It is remarkable that the experimental data from both species are very similar (bars with the errors in Fig. 5D). Our model reproduces the experimental cAMP level after application of different combinations of IBMX and isoproterenol (bars without errors).

#### cAMP-PKA module

cAMP molecules generated in cardiac myocytes by adenylyl cyclases and partially hydrolyzed by phosphodiesterases activate protein kinase A, a major signaling molecule in the β_1_-adrenergic signaling system which phosphorylates ion channels and proteins of the Ca^2+^ handling system. Inactive PKA holoenzyme consists of two regulatory subunits R (both either of type I, RI, or type II, RII) and two catalytic subunits C. Activation of PKA holoenzyme requires binding four cAMP molecules, in general, with different affinities. PKA holoenzyme with bound four cAMP molecules dissociates two catalytic subunits, which phosphorylates target proteins. In our model, we consider identical affinities for all four binding sites of PKA. Two isoforms of PKA holoenzyme are found in mouse ventricular myocytes, PKAI and PKAII, which differ by their regulatory subunits. PKAI is predominantly located in the cytosolic compartment, and PKAII is the predominant isoform in the caveolae and extracaveolae compartments.

PKAI and PKAII differ not only by their location, but also by their half-activation constants, *K_PKAI_* and *K_PKAII_*. Some experimental data suggest half-activation constants for *K_PKAI_* ∼ 0.1 µM [50], [51] and *K_PKAII_* ∼ 0.5 µM [51], one of which is significantly smaller and the other is comparable to the resting cAMP concentrations in cardiac cells ∼ 0.5–1 µM [52], [53]. Under such conditions, almost all PKA in the cytosol and most PKA in the caveolae and extracaveolar compartments must be activated. Other experimental data suggest *K_PKAI_* and *K_PKAII_* in the range ∼ 1.5–3 µM [54], [55], [56]. As it was pointed out by Dao et al. [55], relatively small values of *K_PKAI_* and *K_PKAII_* in the nanomolar range were obtained in some experiments because they used free regulatory subunits RI or RII instead of PKA holoenzymes. Experimental data with PKA holoenzymes yielded significantly larger dissociation constants, which are used in our model (*K_PKAI,1_* and *K_PKAI,2_* = 2.9 µM [55]; *K_PKAII,1_* and *K_PKAII,2_* = 2.5 µM [8], [54]). Activation of PKA holoenzyme is described by ordinary differential equations (see Appendix S1). In addition, the model includes inhibition of PKA by heat-stable protein kinase inhibitor (PKI), the dynamics of which are also described by a differential equation.

**Figure 6:**
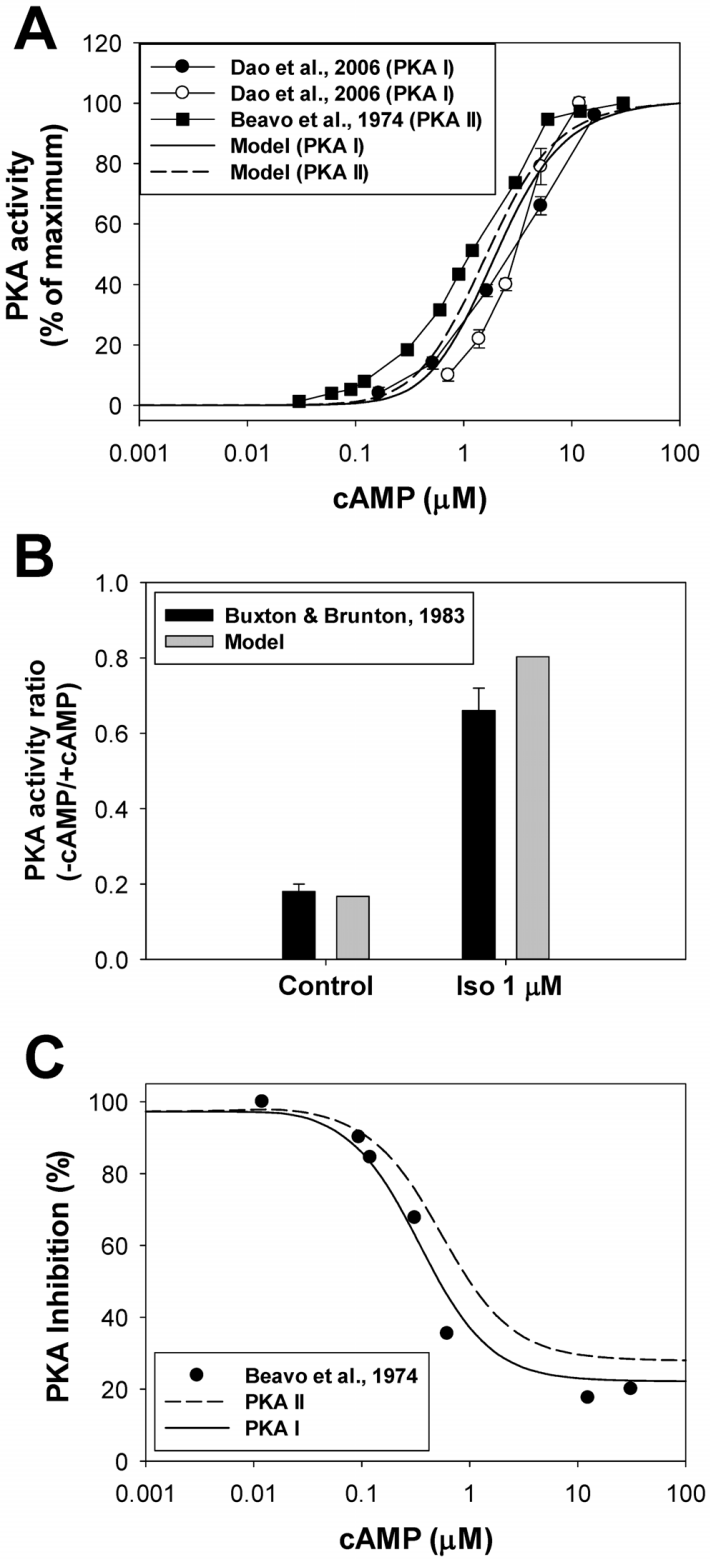
The effects of β_1_-adrenoceptor stimulation on PKA activities. **Panel A**: PKA I and PKA II activities as functions of cAMP. Experimental data for PKA I obtained by two methods are shown by filled and unfilled circles [55]; data for PKA II obtained by Beavo et al. [54]. Corresponding simulated data are shown by a solid (PKA I) and a dashed (PKA II) line. **Panel B**: PKA activity ratio. Experimental data were obtained without (−cAMP) and with (+cAMP) externally applied 3 µM cAMP, both without and with 1 µM isoproterenol (black bars [57]). We also performed four simulations: no isoproterenol/basic level cAMP (−cAMP), no isoproterenol/3 µM cAMP (+cAMP), 1 µM isoproterenol/no externally applied cAMP (−cAMP), and 1 µM isoproterenol/3 µM cAMP (+cAMP). Then, the corresponding PKA ratios were calculated. **Panel C**: Protein kinase A inhibition by a heat-stable protein kinase inhibitor PKI. Experimentally, PKA activities were measured with and without PKI at different concentrations of cAMP, then their ratio was calculated (in %) and subtracted from 100% (filled circles, [54]). Corresponding simulation data for PKA I and PKA II are shown by solid and dashed lines. Concentration of [*PKI*]_tot_ = 2·0.2·[*PKA*]_tot_.

Figure 6A shows activation levels of PKAI and PKAII as functions of cAMP concentrations. We used experimental data [54], [55] shown by circles (PKAI) and squares (PKAII). Our simulations are displayed by a solid (PKAI) and a dashed (PKAII) line, respectively. They fit reasonably well to the experimental data. We also simulated the cellular PKA activity ratio in control and upon stimulation of the β_1_-adrenergic signaling system by 1 µM isoproterenol (Fig. 6B). Four simulations were performed for this figure: no isoproterenol/basic cAMP level (−cAMP), no isoproterenol/3 µM cAMP (+cAMP), 1 µM isoproterenol/no externally applied cAMP (−cAMP), and 1 µM isoproterenol/3 µM cAMP (+cAMP). Then, the corresponding PKA(−cAMP)/PKA(+cAMP) ratios were calculated. The simulations (grey bars) compare well to the experimental data for the rabbit hearts obtained in control and after application of 1 µM isoproterenol (black bars [57]).

The effect of a heat-stable protein kinase inhibitor (PKI) on the PKA activity is shown in Fig. 6C. Experimental data obtained from [54] for PKA II are shown by filled circles, and simulation results are plotted by a solid line for PKA I and by a dashed line for PKA II. PKA activities are calculated with and without PKI at different concentrations of cAMP, then their ratios were calculated (in %) and subtracted from 100%. As seen from Fig. 6C, model simulations fit reasonably well to the experimental data.

#### Protein phosphatases and inhibitor-1 module

There are two major types of protein phosphatases which are important for cardiac myocyte function, protein phosphatase 1 (PP1) and 2A (PP2A). Localization of PP1 and PP2A in three subcellular compartments can be determined by their co-localization with caveolin-3 and by modulation of their targets. Data of Hescheler et al. [58] and Balijepalli et al. [19] show that the L-type Ca^2+^ current is inhibited by both PP1 and PP2A, and that the portion of the L-type Ca^2+^ channels and PP2A co-localize with caveolin-3, suggesting caveolae localization for both PP1 and PP2A. PP1 and PP2A are also localized in the cytosolic compartment, as they interact with phospholamban and troponin I [59], [60]. In mouse hearts, PP1 is the predominant phosphatase, whose contribution to the total phosphatase activity is ∼75% [61], [62]. As there is no current consensus on whether PP1 or PP2A is predominant in the extracaveolae compartment [63], we assume that only PP1 contributes to phosphatase activity in extracaveolae. We use basically the same molar distribution of PP1 and PP2A in subcellular compartments as Heijman et al. [8] (see Appendix S1), provided that 75% and 25% of cellular phosphatase are of PP1 and PP2A, respectively, as found experimentally in mice [61], [62].

In ventricular myocytes, protein phosphatase 1 is regulated by endogenous inhibitor-1 (I-1) [64]. I-1 is localized in the cytosolic compartment and inhibits PP1 activity when phosphorylated. Cellular concentration of I-1 is estimated as 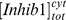 = 0.08543 µM [65]. Ablation of I-1 leads to a moderate increase in PP1 activity and impaired β_1_-adrenergic function [64], [65]. As the affinity of I-1 to PP1 is very high (*K_Inhib1_* = 1 nM), we used steady-state approximation to describe I-1-PP1 interaction with corresponding mass conservation relationships:
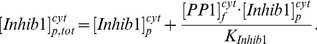
(3)

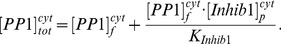
(4)

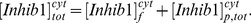
(5)where 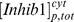 is the total concentration of phosphorylated I-1 in cytosol, 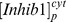 is the unbound phosphorylated I-1 concentration, 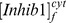 is the non-phosphorylated I-1 concentration,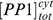 is the total concentration of PP1 in cytosol, and 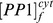 is the unbound PP1 concentration. Solution of equations (3) – (5) gives the equations for calculating 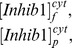 and 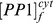 (see equations (A.124) – (A.129) in the Appendix S1). Phosphorylation and dephosphorylation of I-1 occurs by the catalytic subunit of PKA and PP2A, respectively [64], [66], and is described by equation (A.130) (see Appendix S1).

**Figure 7:**
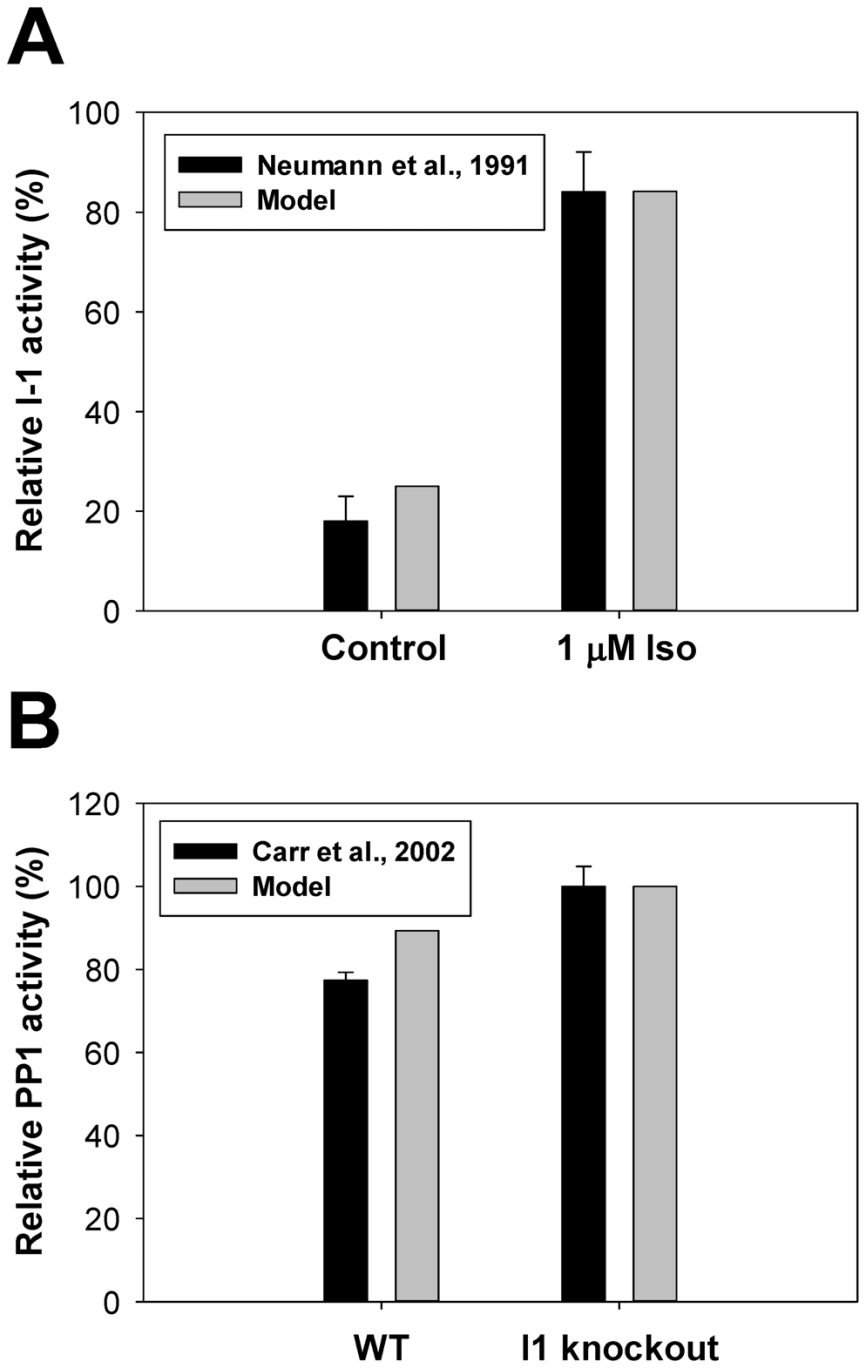
The effects of β_1_-adrenoceptor stimulation on activities of I-1 and PP1. **Panel A**: Relative I-1 activity in ventricular myocytes in control (left bars) and upon stimulation with 1 µM isoproterenol (right bars). Experimental data for guinea pig hearts [67] are shown by black bars, simulation data with our model are shown by gray bars. **Panel B**: Relative PP1 activity in WT and I-1 knockout mouse hearts. Experimental data [64] are shown by black bars, our simulations – by gray bars. Experimental PP1 activity from I-1 knockout mouse hearts is normalized to 100%.

Figure 7A shows the effects of activation of the β_1_-adrenergic signaling system by 1 µM isoproterenol on inhibitor-1 activity. As there is no data for mice, we used the experimental data from guinea pig hearts [67] which shows significant activation of I-1 from ∼20% to ∼80% of its maximum activity. Our model reproduced the experimental data on I-1 activation. The effects of I-1 on PP1 activity can be estimated from data obtained from WT and I-1 knockout mice [64]. Figure 7B shows a slight increase in PP1 activity upon I-1 ablation (activity of PP1 in I-1 knockout mice is normalized to 100%). In the model, the effect of I-1 knockout is simulated by setting 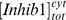 = 0, which leads to a slightly smaller increase in PP1 activity compared to that found experimentally.

#### cAMP dynamics

cAMP is a one of the major signaling small molecules in the β_1_-adrenergic signaling system. It moves freely between cellular compartments and has PKA as a major target. cAMP concentration is determined by the balance between cAMP production by adenylyl cyclases, cAMP degradation by phosphodiesterases, and cAMP diffusion between intracellular compartments (caveolae, extracaveolae, and cytosol) (see (A.131)-(A.133) in Appendix S1). Experimental data with unstimulated cells shows cAMP concentrations in mouse ventricular myocytes in the range from 2.4 to 6.0 pmol/mg protein (0.4522–1.13 µM) [43], [52], [53]. Our model gives a background cellular cAMP concentration ∼ 0.3 µM. This concentration is close to the experimental values and to the values obtained in the models of others [8], [11].

**Figure 8:**
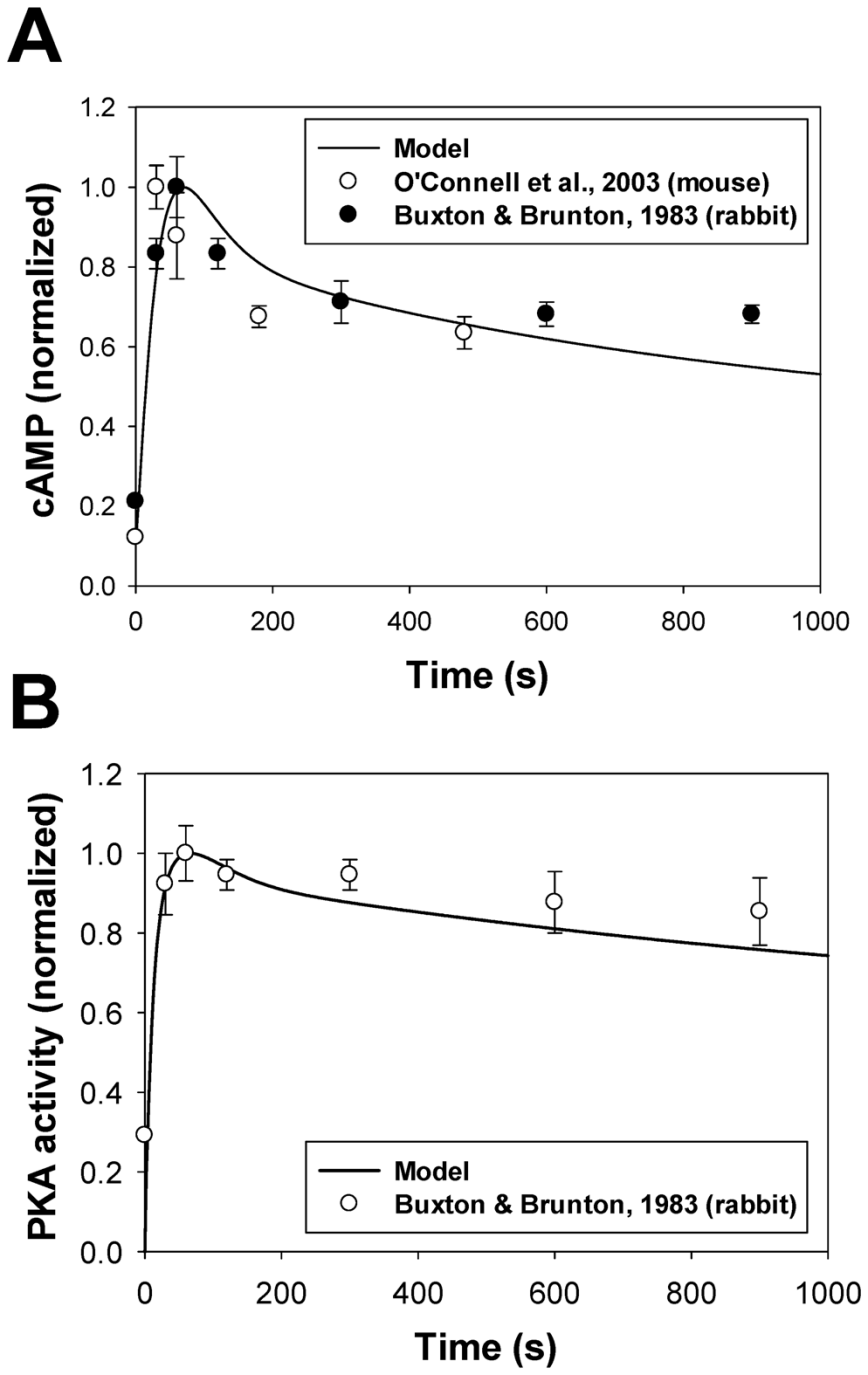
cAMP and PKA dynamics in mouse ventricular myocytes. **Panel A**: cAMP dynamics in ventricular myocytes. Experimental data of normalized cAMP in mouse [68] and rabbit [57] ventricular myocytes are shown by unfilled and filled circles, respectively; simulation data is shown by a solid line. **Panel B**: PKA dynamics in ventricular myocytes. Experimental data of normalized PKA activity in rabbit [57] ventricular myocytes are shown by unfilled circles; simulation data is shown by a solid line. Data in **Panels** **A** and **B** was obtained upon application of 1 µM isoproterenol.

Stimulation of the β_1_-adrenergic signaling system results in an increase of cAMP production by adenylyl cyclases and cAMP degradation by phosphodiesterases in the caveolae, extracaveolae, and cytosolic compartments. Figure 8A shows experimental time courses of normalized cAMP in mouse [68] and rabbit [57] ventricular myocytes obtained upon stimulation with 1 µM isoproterenol (unfilled and filled circles, respectively). A solid line in Fig. 8A shows corresponding simulated time behavior of cAMP. Both simulated and experimental data show transient increase in cAMP concentration, which is due to changes in cAMP production by adenylyl cyclases, cAMP degradation by phosphodiesterases, cAMP fluxes between compartments, and β_1_-ARs desensitization.

#### PKA dynamics

Our model is able to reproduce the time course of the cellular PKA activity, which is determined by the cellular concentration of catalytic subunit (solid line in Fig. 8B). As there is no data for mice, we used data of Buxton and Brunton [57] (unfilled circles in Fig. 8B) obtained for rabbit ventricular myocytes to constrain the model. Similar to cAMP dynamics, both experimental and simulation data for PKA demonstrate transient increase in activation, with subsequent decrease in activity.

### Model Development: Electrophysiological Part

Electrical activity of the mouse ventricular myocytes is described by the equation for transmembrane potential [25]:
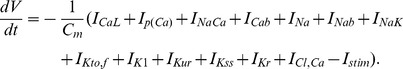
where I_CaL_ is the L-type Ca^2+^ current, I_p(Ca)_ is the sarcolemmal Ca^2+^ pump, I_NaCa_ is the Na^+^/Ca^2+^ exchanger, I_Cab_ is the Ca^2+^ background current, I_Na_ is the fast Na^+^ current, I_Nab_ is the Na^+^ background current, I_NaK_ is the Na^+^-K^+^ pump, I_Kto,f_ is the rapidly recovering transient outward K^+^ current, I_K1_ is the time-independent K^+^ current, I_Kur_ is the ultrarapidly activating delayed rectifier K^+^ current, I_Kss_ is the noninactivating steady-state voltage activated K^+^ current, I_Kr_ is the rapid delayed rectifier K^+^ current, I_Cl,Ca_ is the Ca^2+^-activated chloride current, and I_stim_ is the stimulus current.

In our model, we consider four of these currents (I_CaL_, I_Na_, I_Kto,f_, and I_Kur_) as the substrates of the β_1_-adrenergic signaling system. The Na^+^/K^+^ pump is affected by β_1_-ARs through phosphorylation of phospholemman. One more current, I_K1_, is also affected by β_1_-ARs; however, in the voltage range from about −80 to +40 mV, this current does not change significantly. In addition, there are three other phosphorylation substrates, which are the major players in Ca^2+^ dynamics and are affected by β_1_-ARs: ryanodine receptors, phospholamban, and troponin I. We consider all these β_1_-adrenoceptor substrates below as separate modules in the model.

#### L-type Ca^2+^ current module

As found experimentally, the L-type Ca^2+^ channels are localized in both the caveolae and the extracaveolae compartments [19], [21], [22], [69]. About 80% of the L-type Ca^2+^ channels are found within Ca^2+^ release units, or couplons [21], which are localized in the extracaveolae compartment, contribute directly to excitation-contraction coupling, and are the targets of the β_1_-adrenergic signaling system [22], [70]. The other 20% of the L-type Ca^2+^ channels are found in the caveolae [19], [22]. The total amount of L-type Ca^2+^ channel protein can be estimated from the experimental data on the amount of RyRs in the mouse hearts (1058±45 fmol/mg protein, or 0.1993 µM [71]) and dihydropyridine (DHPR) receptor-to-RyRs ratio for rats (RyR/DHPR = 7.3 [72]), resulting in [I_CaL_]_tot_ = 0.0273 µM. In the presented model, 20% and 80% of the L-type Ca^2+^ channels are localized in the caveolae and the extracaveolae compartments, respectively.

**Figure 9:**
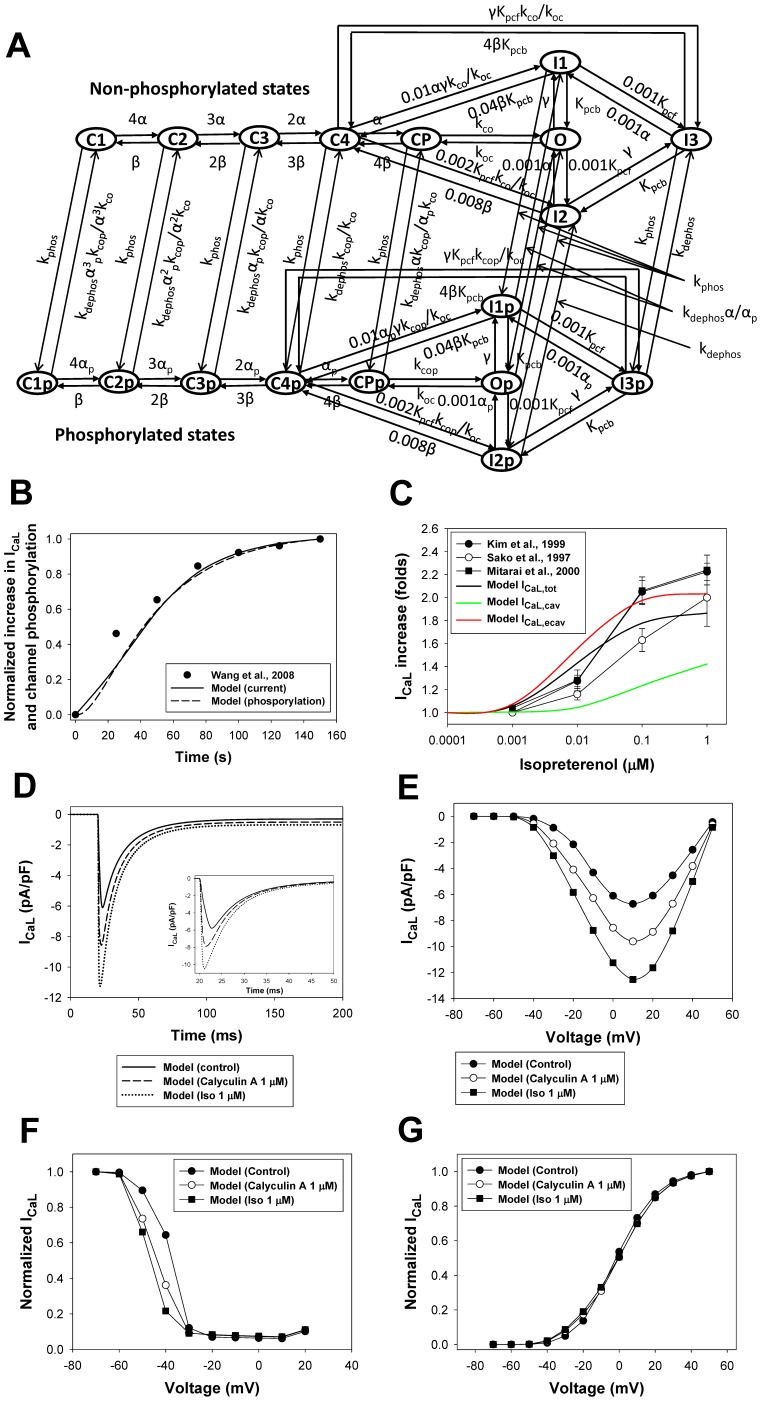
The effects of β_1_-adrenoceptor stimulation on the L-type Ca^2+^ current. **Panel A:** Markov model of the L-type Ca^2+^ channel. State diagram consists of two similar sub-diagrams for non-phosphorylated (upper sub-diagram) and phosphorylated states (lower sub-diagram). C_1_, C_2_, C_3_, C_4_, and C_P_ are closed states; O is the open state; I_1_, I_2_, and I_3_ are inactivated states; C_1p_, C_2p_, C_3p_, C_4p_, and C_Pp_ are closed phosphorylated states; O_p_ is the open phosphorylated state; and I_1p_, I_2p_, and I_3p_ are phosphorylated inactivated states. The rate constants α, α_p_, and β are voltage-dependent; γ is calcium dependent; k_co_, k_oc_, k_cop_, K_pcf_ and K_pcb_, are voltage-insensitive; and k_phos_ and k_dephos_ are the phosphorylation and dephosphorylation rates, respectively (see Appendix S1). **Panel B:** Time course of the peak values of L-type Ca^2+^ current and L-type Ca^2+^ channel phosphorylation level upon stimulation with 1 µM isoproterenol. Experimental data for peak I_CaL_ is obtained by a series of pulses to 0 mV for 200 ms from a holding potential of −80 mV with a frequency 0.2 Hz [75]. Modeling data are obtained by a series of pulses to 0 mV for 50 ms from a holding potential of −80 mV with a frequency 0.04 Hz. Increase in phosphorylation level is determined as a fractional increase related to the total increase in phosphorylation of L-type Ca^2+^ channels at 150^th^ s after application of isoproterenol. **Panel C:** Peak L-type Ca^2+^ current as a function of isoproterenol concentration. Experimental data are obtained by Kim et al. [79] (filled circles), Sako et al. [80] (unfilled circles), and Mitarai et al. [81] (filled squares). Simulation data is obtained by a voltage pulse to 0 mV from a holding potential of −80 mV after a 600-second exposure to different concentrations of isoproterenol. Simulation data for the total cellular I_CaL,tot_, the caveolae-localized I_CaL,cav_, and the extracaveolae-localized I_CaL,ecav_ are shown by black, green, and red solid lines, respectively. **Panel D:** Simulated time course of the L-type Ca^2+^ currents in control (solid line), after 1000-s exposure to PP1/PP2A inhibitor Calyculin A (65% of PP1/PP2A activity inhibition, dashed line), and after 600-s exposure to 1 µM isoproterenol (dotted line). Currents are obtained by a voltage pulses to 0 mV from a holding potential of −80 mV and without Ca^2+^-induced Ca^2+^ release to account for heavy buffer conditions. Insert in **Panel D**: Same simulations performed with intact Ca^2+^-induced Ca^2+^ release. **Panel E:** Peak current-voltage (I–V) relationships for I_CaL_ in control (filled circles) and after exposure to Calyculin A (unfilled circles) and isoproterenol (filled squares). **Panel F:** Steady-state inactivation relationships for I_CaL_ in control (filled circles) and after exposure to Calyculin A (unfilled circles) and isoproterenol (filled squares). **Panel G:** Normalized maximum conductance (G/G_max_) for I_CaL_ as functions of voltage in control (filled circles) and after exposure to Calyculin A (unfilled circles) and isoproterenol (filled squares). In **Panels E, F, and G**, currents are obtained by the two-pulse protocols: a 500-ms depolarizing first pulse to between −70 and +50 mV (in 10-mV increment) is applied from a holding potential of −80 mV; this is followed by a second 500-ms pulse to +10 mV. Simulations are performed without Ca^2+^-induced Ca^2+^ release to account for heavy buffer conditions.

Effects of the β_1_-adrenergic signaling system on the L-type Ca^2+^ channels and channel gating can be described with a Markov model for non-phosphorylated and phosphorylated states (Fig. 9A). The model includes two activation pathways: one for non-phosphorylated and one for phosphorylated channels. Each pathway includes five closed, one open, and three inactivated states. Transitions between non-phosphorylated and phosphorylated states are determined by the rates of phosphorylation by PKA and dephosphorylation by PP1 and PP2A (see Appendix S1). As we were unable to reproduce the experimental data on isoproterenol effects on the L-type Ca^2+^ channels with the Markov model from [25], [26] due to the high maximum opening probability for the channels at large depolarizations, we introduced a voltage-independent rate limiting step and a new closed state (C_P_, closed pre-open state) in the new model (Fig. 9A), which reduced the opening probability for non-phosphorylated channels compared to the models of Bondarenko et al. [25] and Petkova-Kirova et al. [26]. Experimental data shows that the stimulation of β_1_-ARs increases the magnitude of the L-type Ca^2+^ current and causes a hyperpolarization shift in normalized channel conductance (G/G_max_) and a steady-state inactivation relationship in mouse ventricular myocytes [73], [74], [75]. These effects are simulated by several changes to the phosphorylated pathway in the model (see Appendix S1) and an increase in G_CaLp_ (G_CaLp_ = 2.09G_CaL_ according to the estimation of an increase in maximum opening probability for cardiac Ca^2+^ channels from [76]).

Model parameters are adjusted to fit experimental data on both the basal L-type channel phosphorylation level and the time course of the current amplitude upon stimulation with isoproterenol. Experimental data suggest the basal phosphorylation level of the L-type Ca^2+^ channels in ventricular myocytes to be about 13–20% [77], [78]. In our model, a fraction of phosphorylated channels under basal conditions is set to 10.3%. Upon application of 1 µM isoproterenol, peak values of I_CaL_ increase in time upon stimulation with voltage pulses to 0 mV, as is seen from the experimental data [75] (Fig. 9B). Our model reproduced the time course of the peak current increase under similar stimulations (solid line in Fig. 9B). It is remarkable that the simulated time course of the peak current increase (solid line in Fig. 9B) is very similar to the simulated time course of the relative phosphorylation level of the L-type Ca^2+^ channels (dashed line in Fig. 9B).

In addition to the time course of I_CaL_ increase, we simulated magnitudes of I_CaL_ increase after a 600-second exposure to different concentrations of isoproterenol (Fig. 9C). We evaluated the effects of isoproterenol on the total I_CaL_ (I_CaL,tot_, black bold solid line in Fig. 9C) as well as on I_CaL_ populations localized in the caveolae (I_CaL,cav_, green line in Fig. 9C) and extracaveolae (I_CaL,ecav_, red line in Fig. 9C) compartments. The results of simulations for the total I_CaL_ are in a good agreement with the experimental data obtained by Kim et al. [79], Sako et al. [80], and Mitarai et al. [81] (Fig. 9C). Simulations also show that the effects of β_1_-adrenergic receptor agonist is significantly stronger on the channels localized in the extracaveolae compartment (2.03 folds at 1 µM Iso) compared to the channels in the caveolae compartment (1.42 folds at 1 µM Iso). If the caveolae-located I_CaL_ is responsible for ∼20% of the total current amplitude in control, after exposure to isoproterenol (1 µM) this fraction decreases to ∼15%. The results of simulations are in line with the experimental data of [70] who have shown that the I_CaL_ from the population of channels in the caveolae compartment comprises about 15% of the total current.

In addition to the effects of activation of the β_1_-adrenergic signaling system, our model is tested in respect to the effects of inhibition of phosphatase activity on the L-type Ca^2+^ current. Figure 9D shows simulated I_CaL_ traces under heavy buffer conditions (with suppression of Ca^2+^-induced Ca^2+^ release) elicited by voltage pulses to 0 mV in control (solid line) and after 1000-s exposure to 1 µM Calyculin A (dashed line) and 600-s exposure to 1 µM isoproterenol (dotted line). In simulations, we consider that Calyculin A inhibits 65% of the activity of both PP1 and PP2A. As seen from simulations in Fig. 9D, both isoproterenol and Calyculin A increase peak currents to different degrees, suggesting that both activation of PKA and inhibition of PP1 and PP2A increase the L-type Ca^2+^ current. Fitting simulated time courses from Fig. 9D yields bi-exponential inactivation of I_CaL_ with two time constants. Fast (Ca^2+^-dependent inactivation) and slow (voltage-dependent inactivation) time constants are equal to 7.77 and 22.0 ms, 7.19 and 19.9 ms, and 6.54 and 18.5 ms for control (solid line), after 1000-s exposure to 1 µM Calyculin A (dashed line), and 600-s exposure to 1 µM isoproterenol (dotted line), respectively. These data show significant increase in Ca^2+^-dependent inactivation of I_CaL_ upon stimulation of the β_1_-adrenergic signaling system. Similar behavior of I_CaL_ is also observed experimentally with BAPTA in pipette solution [73]. Insert in Fig. 9D shows similar simulations with intact Ca^2+^-induced Ca^2+^ release. In the last case, current traces also show bi-exponential decay with the fast and slow time constants 4.83 and 12.4 ms, 4.72 and 12.0 ms, and 4.66 and 12.8 ms for control (solid line), after 1000-s exposure to 1 µM Calyculin A (dashed line), and 600-s exposure to 1 µM isoproterenol (dotted line), respectively. These data show significant increase in the L-type Ca^2+^ current inactivation with intact Ca^2+^ dynamics. Simulations also show that activation of β_1_-ARs increases peak current amplitudes by about two-fold (Fig. 9E) and causes a hyperpolarization shift in steady-state inactivation relationships (Fig. 9F) and more moderately in G/G_max_ data (Fig. 9G). Inhibition of PP1 and PP2A produced an intermediate effect on the I_CaL_ in terms of an increase in amplitude and a hyperpolarization shift of steady-state inactivation relationships and G/G_max_. Similar increases in amplitudes and hyperpolarization shifts are also observed experimentally [73]. Therefore, our model for the β_1_-adrenergic signaling system is able to simulate the effects of both activation of PKA and inhibition of PP1 and PP2A on the L-type Ca^2+^ current, I_CaL_.

#### Fast Na^+^ current module

The channels responsible for the fast Na^+^ current, I_Na_, which are encoded by Nav1.5 subunit, are found in the caveolae compartment [16], [69], [82]. Using both immunoblot analysis and imaging technique, Yarbrough et al. [82] provided evidence of co-localization of the fast Na^+^ channels and caveonin-3 in rat cardiomyocytes, suggesting their localization in the caveolae compartment. Similar data were also obtained by Shibata et al. [69] by using immunogold labeling of plasma membranes from rat cardiomyocytes, where Nav1.5 subunits were co-localized on electron microscope images with caveolin-3. In addition, Palygin et al. [83] provided evidence of the involvement of caveolin-3 in isoproterenol-induced enhancement of I_Na_ in ventricular myocytes.

Activation of the β_1_-adrenergic signaling system with isoproterenol leads to an increase in the amplitude of I_Na_, however, without the effects on gating properties of the channel [84], [85]. Simultaneous activation of several components of the β_1_-adrenergic signaling system with cAMP-IBMX-forskolin cocktail or with excessive application of membrane permeant analogue of cAMP, CPTcAMP (5 mM), results in additional small shift in the G/G_max_ and steady-state inactivation [86], [87], [88]. Experimental data of Baba et al. [86] also shows that the inhibition of protein phosphatase PP2A causes an increase in I_Na_, and the addition of PP2A to pipette solution causes a decrease in I_Na_, which link phosphorylation level of I_Na_ to its magnitude. Mechanism of the changes in I_Na_ was studied by Zhou et al. [88] in detail. It was shown that, in addition to channel phosphorylation, an increased number of functional fast Na^+^ channels in the cell membrane is due to the channel trafficking. More recent analysis of the effects of PKA on I_Na_ suggested that phosphorylation and trafficking of I_Na_ are related processes: channels’ phosphorylation increases trafficking of fast Na^+^ channels [89].

**Figure 10:**
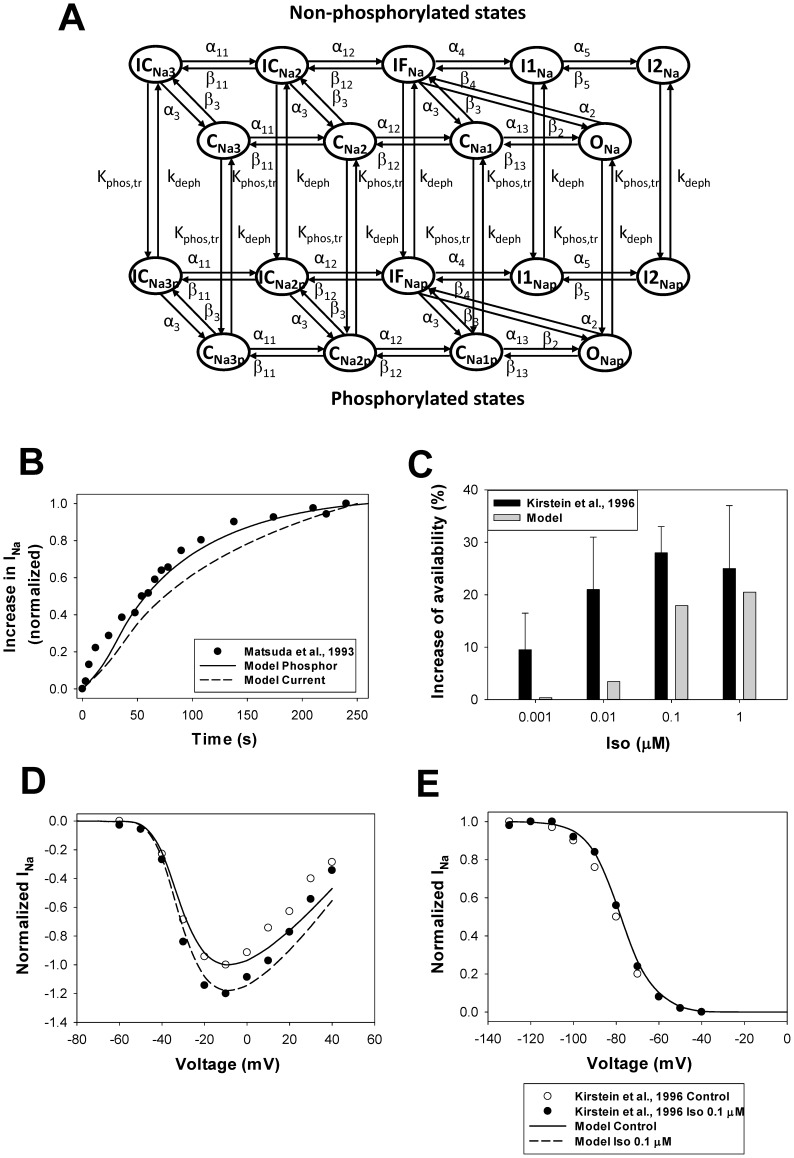
The effects of β_1_-adrenoceptor stimulation on the fast Na^+^ current. **Panel A:** Markov model of the fast Na^+^ channel. State diagram consists of two similar sub-diagrams for non-phosphorylated (upper sub-diagram) and phosphorylated-trafficked states (lower sub-diagram). C_Na1_, C_Na2_, and C_Na3_ are closed states; O_Na_ is the open state; IF_Na_, I1_Na_, and I2_Na_ are the fast, intermediate, and slow inactivated states, respectively; IC_Na2_ and IC_Na3_ are closed-inactivated states; C_Na1p_, C_Na2p_, and C_Na3p_ are closed phosphorylated states; O_Nap_ is the open phosphorylated state; and IF_Nap_, I1_Nap_, I2_Nap_, IC_Na2p_, and IC_Na3p_ are phosphorylated inactivated states. The rate constants for activation, deactivation, inactivation, phosphorylation-trafficking, and dephosphorylation are given in Appendix S1. **Panel B:** Time course of the activation of the fast Na^+^ current upon application 0.1 µM isoproterenol. Experimental data of Matsuda et al. [85] obtained for the normalized peak I_Na_ in rabbit ventricular myocytes is shown by closed circles. Data is obtained with 40-ms pulses from a holding potential of −100 mV to −30 mV at stimulation frequency 0.2 Hz. A solid line shows the time course of simulated data on relative I_Na_ phosphorylation upon application of 0.1 µM isoproterenol. A dashed line shows the time course of the simulated normalized peak I_Na_ after application of 0.1 µM isoproterenol. The simulated currents are obtained with 20-ms pulses from a holding potential of −140 mV to −30 mV at stimulation frequency 0.04 Hz. **Panel C:** An increase in peak I_Na_ availability upon application of different concentrations of isoproterenol (in %). Experimental data by Kirstein et al. [84] obtained from rat ventricular myocytes are shown by black bars with errors; corresponding simulation data are shown by gray bars. Peak current-voltage (**Panel D**) and steady-state inactivation (**Panel E**) relationships for the fast Na^+^ current in ventricular myocytes upon stimulation with 0.1 µM isoproterenol. Experimental data for rats in the absence (unfilled circles) and presence (filled circles) of 0.1 µM isoproterenol are obtained by Kirstein et al. [84] (holding potential is −100 mV, conditioning pulse duration is 2,500 ms; isoproterenol data is obtained after 10 min of application). Simulated data are shown by solid (no isoproterenol) and dashed (10 min after application of 0.1 µM isoproterenol) lines (data are obtained by two-pulse protocol, holding potential is −140 mV, first pulse duration is 500 ms for voltages from −140 to +40 mV in 10 mV steps, second pulse duration is 50 ms at voltage −20 mV). Isoproterenol increases I_Na_ availability, but does not affect gating properties.

To simulate these effects, we developed a Markov model for I_Na_, based on the model [25], [90], in a similar way as for I_CaL_, but with some differences. We consider that the amplification of I_Na_ occurs through two processes: phosphorylation of the channels and their subsequent trafficking to the cell membrane. As the kinetics of the two processes are indistinguishable in the experiments, we use only one rate constant to characterize the effect of PKA, k_phos,tr_, which includes both channel’s phosphorylation and trafficking. Two activation pathways are used, one is for non-phosphorylated channels and another is for phosphorylated-trafficked channels (Fig. 10A). Because isoproterenol does not change the gating properties of I_Na_, we use the same rate constants for both non-phosphorylated and phosphorylated-trafficked channels. The larger conductance is used for the phosphorylated-trafficked channels (see the fast Na^+^ current module in Appendix S1). We consider that the fast Na^+^ channels are dephosphorylated by PP1 and PP2A (Fig. 10A, Appendix S1). The set of equations that describes the fast Na^+^ channel gating is provided in Appendix S1 ((A.180) - (A.213)).

The resulting model for the fast Na^+^ current was tested against the experimental data on the time course of I_Na_ activation, its dependence on the isoproterenol concentration, and voltage-clamp experiments (Fig. 10). As there is no data for mice, we used the experimental time course of the activation of I_Na_ by 0.1 µM isoproterenol obtained from rabbit ventricular myocytes (filled circles in Fig. 10B). Figure 10B shows a good agreement between the experimental and simulated time dependences of I_Na_ amplitude upon stimulation of the β_1_-adrenergic signaling system. A similar time course is demonstrated by the simulated fraction of phosphorylated and trafficked Na^+^ channels (solid line in Fig. 10B). The model also successfully reproduced the concentration dependence of an increase in Na^+^ channel availability at different concentrations of isoproterenol (Fig. 10C).

Experimental data shows an increase in the fast Na^+^ current amplitude upon stimulation with 0.1 µM isoproterenol (Fig. 10D, unfilled and filled circles). A similar increase is obtained from our Markov model for Na^+^ channels (solid and dashed lines in Fig. 10D are for control and 0.1 µM isoproterenol, respectively). Neither the experimental data (unfilled and filled circles in Fig. 10E) nor the simulations (solid and dashed lines in Fig. 10E) demonstrate significant changes in steady-state inactivation relationships.

#### Ryanodine receptor module

Experimental studies of ryanodine receptors in rat ventricular myocytes provided evidence of their co-localization in couplons with the L-type Ca^2+^ channels (in dyads, or subspace volume V_ss_) or in close proximity to those couplons [21], [22], [91]. Between 80 to 85% of ryanodine receptors are localized in V_ss_ and the rest are very close to dyads, which suggests their involvement in Ca^2+^-induced Ca^2+^ release [21], [91]. Very small portions (∼3.5–9.2%) of ryanodine receptors in mouse ventricular myocytes are shown to co-localize with caveolin-3, but outside of couplons, but their functional significance is unclear [35], [92]. Therefore, in the model, we put ryanodine receptors in the extracaveolae compartment. The receptors are subjects of phosphorylation by PKA and dephosphorylation by PP1. The total concentration of RyRs in mouse ventricular myocytes was determined experimentally by Chu et al. [71] and is equal to 0.1993 µM.

**Figure 11:**
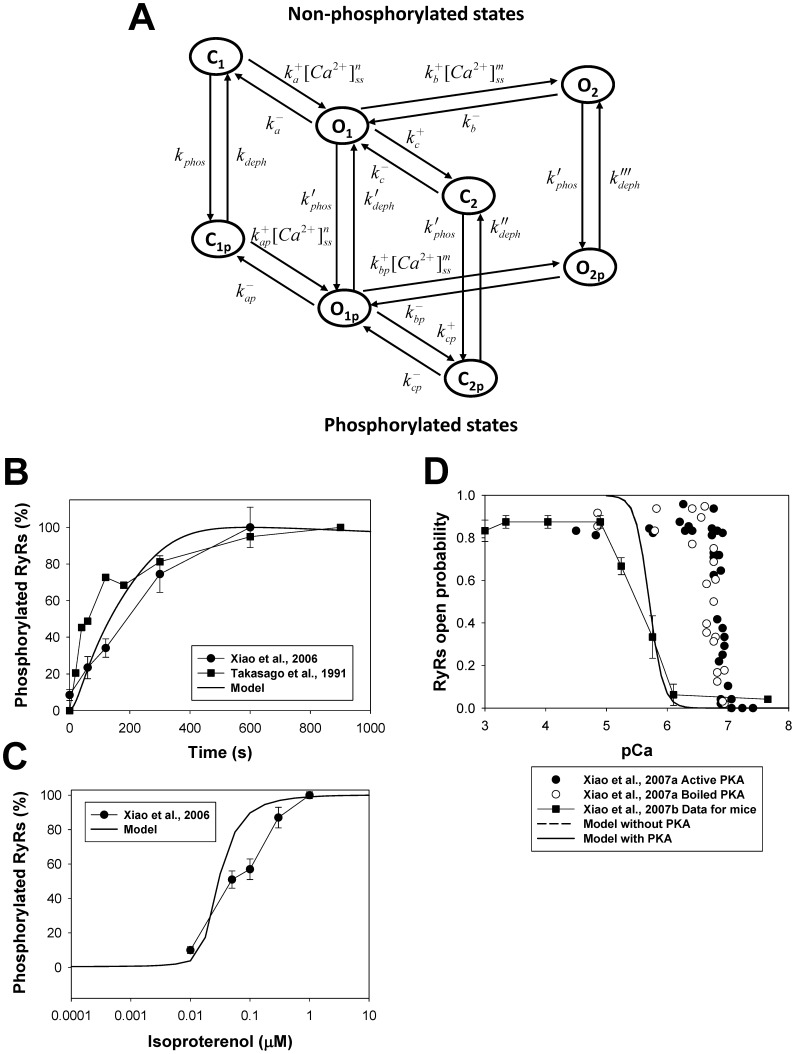
The effects of β_1_-adrenoceptor stimulation on ryanodine receptors. **Panel A:** Markov model of ryanodine receptors. State diagram consists of two similar sub-diagrams for non-phosphorylated (upper sub-diagram) and phosphorylated states (lower sub-diagram). C_1_ and C_2_ are closed states; O_1_ and O_2_ are open states; C_1p_ and C_2p_ are closed phosphorylated states; O_1p_ and O_2p_ are open phosphorylated states. The rate constants from C_1_ to O_1_, from O_1_ to O_2_, from C_1p_ to O_1p_, and from O_1p_ to O_2p_ are Ca^2+^-dependent; and k_phos_ and k_deph_ are the phosphorylation and dephosphorylation rates, respectively (see Appendix S1). **Panel B:** Time course of the relative phosphorylation level of RyRs upon activation of the β_1_-adrenergic signaling system. Experimental data of Xiao et al. [94] (filled circles) are obtained upon stimulation of rat ventricular myocytes with 1 µM isoproterenol; experimental data of Takasago et al. [95] (filled squares) are obtained from canine ventricular myocytes with endogenous PKA by application of [γ-^32^P]ATP. Modeling data are obtained by application of 1 µM isoproterenol. Increase in phosphorylation level is determined as a fractional increase related to the maximum increase in phosphorylation of RyRs after activation of the β_1_-adrenergic signaling system. **Panel C:** Relative phosphorylation level of RyRs at different concentrations of isoproterenol. Experimental data of Xiao et al. [94] (filled circles) obtained upon stimulation of rat ventricular myocytes for 15 minutes. Simulation data are obtained after 10-minute exposure to different concentrations of isoproterenol. **Panel D:** Effects of PKA on opening probability of RyRs as function of cytosolic Ca^2+^ concentration. Experimental data of Xiao et al. [93] are obtained for cardiac RyRs upon application of active (filled circles) and boiled (unfilled circles) PKA at relatively small luminal Ca^2+^ of 45 nM; experimental data of Xiao et al. [96] (filled squares) for RyRs are obtained from mouse ventricular myocytes. Simulation data for mouse ventricular myocytes in the absence and presence of PKA are obtained at intracellular [Ca^2+^]_i_ concentration ranged from 0.01 to 10 µM and are shown by dashed and solid lines. Experimental data obtained at small luminal Ca^2+^ concentration shows larger sensitivity of RyRs to cytosolic Ca^2+^.

A Markov model for ryanodine receptor gating, which includes phosphorylation-dephosphorylation processes, is shown in Fig. 11A. As for two other substrates, I_CaL_ and I_Na_, we developed a Markov model that consists of two populations of the channels, non-phosphorylated and phosphorylated. For non-phosphorylated channels, we employed our previously developed Markov model for RyRs, which contains two closed (C_1_ and C_2_) and two open (O_1_ and O_2_) states [25]. We added two closed-phosphorylated (C_1p_ and C_2p_) and two open phosphorylated (O_1p_ and O_2p_) states to the model and the transitions between corresponding non-phosphorylated and phosphorylated states (Fig. 11A). Because experimental data suggest slightly increased sensitivity of RyRs to cytosolic Ca^2+^ concentration [93], we sped up the forward transition rates to a greater magnitude than the backward transition rates for the phosphorylated channels (see Appendix S1). Differential equations, which describe the time behavior of the RyRs’ probabilities in closed and open states, are shown in Appendix S1 (Eqs. (A.214)-(A.222)).

Our model successfully reproduced the time course of the ryanodine receptor phosphorylation upon stimulation of β_1_-adrenoceptors. Figure 11B shows experimental time behavior of the relative phosphorylation level of RyRs in rat ventricular myocytes during exposure to 1 µM isoproterenol [94]. A similar time course is demonstrated by our model (solid line in Fig. 11B) and by the experimental data which used a different technique of RyR phosphorylation (by endogenous PKA in canine ventricular myocytes upon application of [γ-^32^P]ATP [95]). Figure 11C (filled circles) shows experimental data on the phosphorylation level of RyRs at different concentrations of isoproterenol [94]. Our model is able to reproduce this dependence as well (solid line in Fig. 11C).

In addition, the model simulated dependence of the RyRs open probability as a function of intracellular Ca^2+^ concentration for non-phosphorylated and phosphorylated channels (Fig. 11D). The experimental data on the effects of PKA on RyRs shows a tiny shift of the dependence of phosphorylated RyRs’ opening probability towards a smaller cytosolic Ca^2+^ concentration [93]. However, such data were obtained at relatively small luminal Ca^2+^ concentrations (45 nM), which increased sensitivity of RyRs by an order of magnitude compared to normal physiological conditions (compare the data shown by filled and unfilled circles (small luminal Ca^2+^ concentrations) with the data shown by filled squares (normal physiological luminal Ca^2+^ [96])). To estimate the effects of PKA on RyRs, we simulated two dependences of RyRs opening probabilities on cytosolic Ca^2+^: the first is obtained by stimulation of the cell with intact PKA (solid line in Fig. 11D), the second is obtained by setting [PKA]_tot_ = 0 µM. Our model shows a reasonable agreement of the Ca^2+^-dependence of the RyRs opening probability with the experimental data [96], but virtually no change in the sensitivity of phosphorylated RyRs to cytosolic Ca^2+^ concentration. Some deviations between the experimental (filled squares) and simulated (solid line) data at relatively large cytosolic Ca^2+^ concentrations can be due to the method of estimation of the opening probabilities: in the simulations, we used the maximum values of the channel’s opening probabilities, without consideration of the effects of ryanodine receptor inactivation, while in the single-channel experiments, such inactivation is always present and can potentially reduce measured opening probability.

#### Phospholemman and the Na^+^-K^+^ pump module

Experimental data on the localization of the Na^+^-K^+^ pump shows that ∼30–40% of α_1_-subunits and 80–90% of β_1_-subunits are localized in the caveolae-rich membrane fractions [97]. Another study provided similar data on the Na^+^-K^+^ pump and caveolin-3 co-localization, where ∼70% of those proteins are found in the same fractions [98]. Moreover, almost all the Na^+^-K^+^ pump activity was found in caveolae [97], [99].

**Figure 12:**
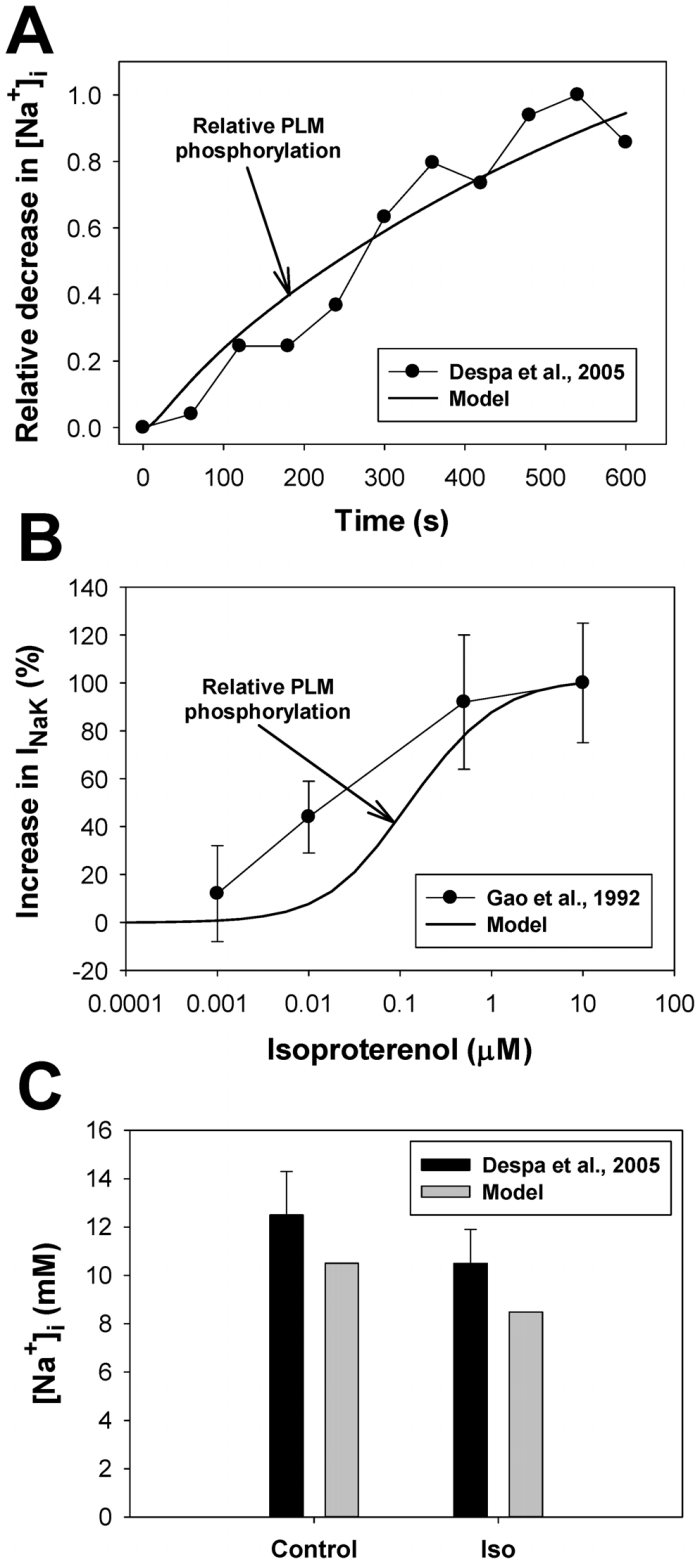
The effects of β_1_-adrenoceptor stimulation on the Na^+^-K^+^ pump. **Panel A:** Experimental time course of a relative decrease in the intracellular [Na^+^]_i_ concentration (filled circles) obtained by Despa et al. [100] from mouse ventricular myocytes after application of 1 µM isoproterenol and corresponding simulated time course of an increase in relative phosphorylation level of phospholemman obtained using our model (solid line). **Panel B:** Experimental data on a relative increase in I_NaK_ current (filled circles) obtained by Gao et al. [101] from guinea pig ventricular myocytes at different concentrations of isoproterenol. Corresponding simulation data with our model on a relative increase in phosphorylation of phospholemman is shown by a solid line. **Panel C:** Experimental (black bars with errors [100]) and simulated (gray bars) data on intracellular [Na^+^]_i_ concentration before (control) and after 10-minutes application of 1 µM isoproterenol (Iso).

In our model, the phospholemman, which regulates the Na^+^-K^+^ pump, is the third substrate of the β_1_-adrenergic signaling system located in the caveolae compartment. Activation of β_1_-ARs increases the function of the Na^+^-K^+^ pump by phosphorylation of phospholemman (PLM) through the effective change in dissociation constant K_m,Nai_ [12], thereby decreasing intracellular [Na^+^]_i_ concentration [100]. An experimental time course of a relative decrease in [Na^+^]_i_ in mouse ventricular myocytes after application of 1 µM isoproterenol is shown in Fig. 12A by filled circles. A corresponding simulated time course of a relative PLM phosphorylation level is shown by a solid line. An increase in the phosphorylation level of PLM decreases the Na^+^ half-saturation constant for the current and effectively increases the pumping rate of Na^+^ outside the cell. Both time dependences demonstrate similar behavior.

Figure 12B shows the experimental dependence of the relative increase in I_NaK_ as a function of isoproterenol concentration obtained from guinea pig ventricular myocytes (filled circles [101]). We compared the experimental data to our simulations of a relative increase in the phosphorylation level for phospholemman (solid line in Fig. 12B). In general, simulation data has a dependence on isoproterenol concentration similar to the experimental data, except for a point at 0.01 µM isoproterenol. Such deviation could be due to the species differences.

Finally, our model successfully reproduced the experimental behavior of intracellular [Na^+^]_i_ concentration in mouse ventricular myocytes measured before and 10 minutes after an application of 1 µM isoproterenol (Fig. 12C, [100]). Both the experimental and simulation data show relatively small, but significant decreases in [Na^+^]_i_ after stimulation of the β_1_-adrenergic signaling system.

#### Ultra-rapidly activating delayed rectifier K^+^ current module

The ultra-rapidly activating delayed rectifier K^+^ current, I_Kur_, is the substrate of PKA in the extracaveolae compartment in cardiomyocytes [15], [102]. I_Kur_ is predominantly encoded by Kv1.5 channels [103]. It is also localized mostly in lipid rafts, which lack caveolin-3 [104], pointing to the extracaveolae compartment (however, see also [105], [106], where localization of Kv1.5 channels is debated). Similar to the Heijman et al. model [8], we put the I_Kur_ current in the extracaveolae compartment.

**Figure 13:**
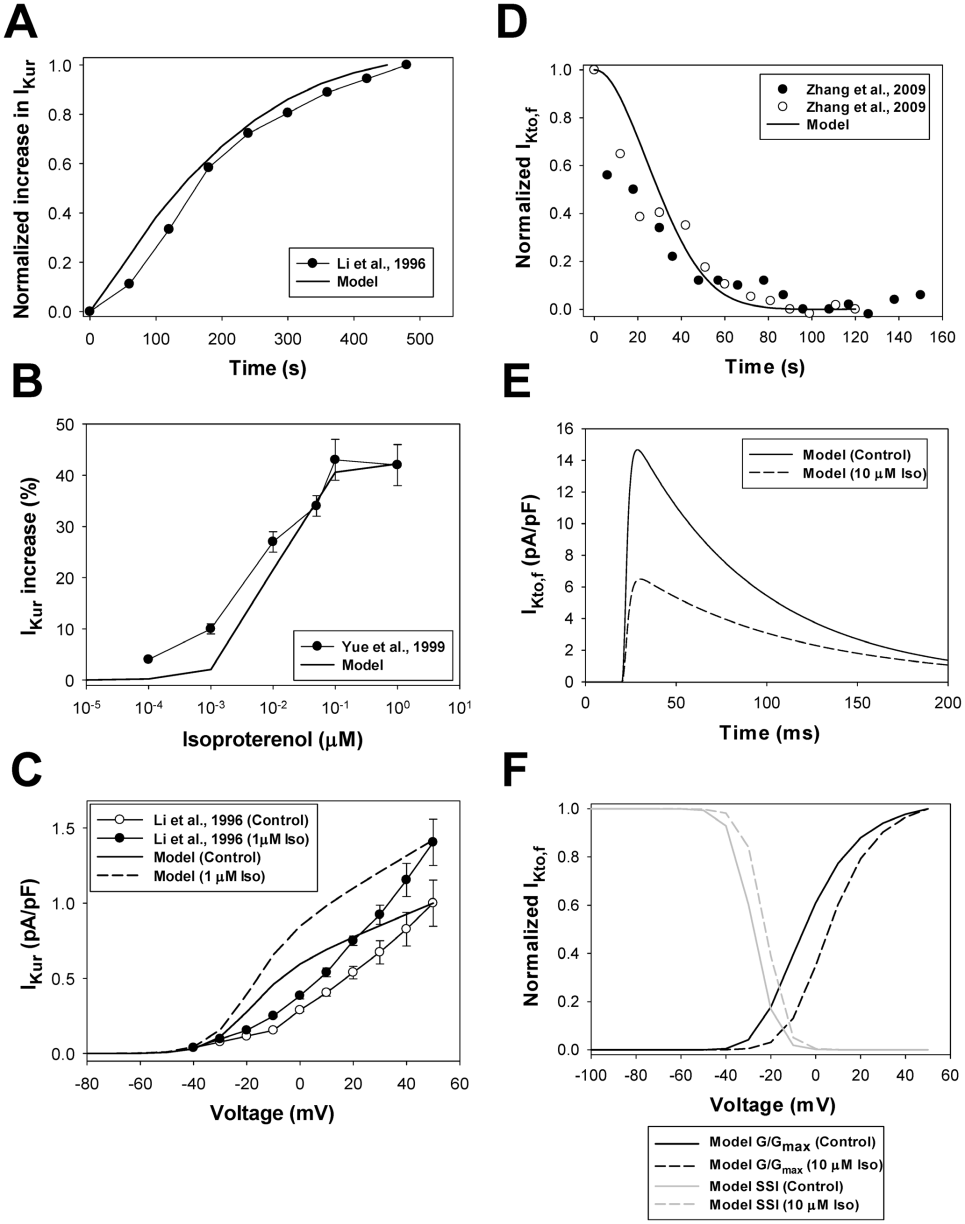
The effects of β_1_-adrenoceptor stimulation on I_Kur_ and I_Kto,f_. **Panel A:** Experimental time course of the relative increase in the ultra-rapidly activating delayed-rectifier K^+^ current I_Kur_ obtained by Li et al. [107] from human atrial myocytes after application of 1 µM isoproterenol (line with filled circles) and corresponding simulated time course of an increase in I_Kur_ obtained with our model (solid line). Simulation data is obtained with 200-ms pulses from a holding potential of −100 mV to +40 mV at stimulation frequency 0.02 Hz. **Panel B:** Experimental data on a relative increase in I_Kur_ current (filled circles) obtained by Yue et al. [108] from canine atrial myocytes at different concentrations of isoproterenol. Corresponding simulation data with our model on relative increase in I_Kur_ are shown by a solid line. Simulation data is obtained with 4.5-s pulses from a holding potential of −90 mV to 0 mV after 800-s exposure to different concentrations of isoproterenol. **Panel C:** Experimental (human atrial myocytes [107], solid lines with circles) and simulated (solid and dashed lines) data on current-voltage relationships for I_Kur_ current. Experimental data for control conditions and those obtained after application of 1 µM isoproterenol are shown by unfilled and filled circles, respectively. Simulated data for control conditions are shown by a solid line, and 1 µM isoproterenol simulations are shown by a dashed line. Simulated currents are obtained by 4.5-s depolarizing pulses to between −80 and +50 mV (in 10-mV increment) from a holding potential of −90 mV. **Panel D:** Experimental time course of the relative decrease in the rapidly recovering transient outward K^+^ current I_Kto,f_ obtained by Zhang et al. [111] from mouse Schwann cells after application of 1 µM forskolin (filled circles) or 10 µM db-cAMP (unfilled circles), and corresponding simulated time course of a relative decrease in I_Kto,f_ phosphorylation obtained with our model after stimulation with 10 µM isoproterenol (solid line). **Panel E:** Simulated time course of I_Kto,f_ traces obtained by depolarization pulses to −5 mV from a holding potential of −100 mV for control (solid line) and after stimulation with 10 µM isoproterenol (dashed line). **Panel F:** Simulated data for G/G_max_ (black lines) and steady-state inactivation relationships (gray lines) obtained for I_Kto,f_ with two-pulse protocol (P1 stimuli from −100 to +50 mV in 10 mV intervals for 500 ms, following P2 pulse to +50 mV for 500 ms; holding potential is −100 mV) in control (solid lines) and after application of 10 µM isoproterenol (dashed lines).

Stimulation of β_1_-ARs increases the function of I_Kur_ [107]. Figure 13A show an experimental time course of I_Kur_ activation in human atrial myocytes upon application of 1 µM isoproterenol (solid lines with circles, [107]). We adjusted model parameters and performed simulations to fit the experimental time behavior (solid line in Fig. 13A). Our model also reproduced an increase in I_Kur_ at different concentrations of isoproterenol (Fig. 13B). The saturation of the current amplitude found experimentally is observed at isoproterenol concentrations of 0.1 to 1 µM (solid lines with circles [108]). Similar data is obtained from simulations (solid line in Fig. 13B).

Experimental voltage dependence of the peak ultra-rapidly activating delayed rectifier K^+^ current in response to depolarizations in the interval from −40 to +50 mV for control conditions and after application of 1 µM isoproterenol is shown in Fig. 13C [107]. As in [25], simulated data show activation of I_Kur_ at slightly more hyperpolarized potentials to offset the effects of divalent cations, which are used in the experiments to block I_CaL_. However, both simulated and experimental data show similar increases in peak current amplitude upon stimulation of β_1_-ARs (solid and dashed lines in Fig. 13C).

#### Rapidly recovering transient outward K^+^ current module

The other substrate of PKA in the extracaveolae compartment in our model is the rapidly recovering transient outward K^+^ current, I_Kto,f_. I_Kto,f_ is found in lipid rafts that lack caveolin-3 pointing to non-caveolae localization [15], [69], [105]. Heijman et al. [8] also placed I_to1_, which is encoded by *Shal*-type K^+^ channels (Kv4.2/Kv4.3), to the extracaveolae compartment.

There are only a few qualitative experimental data on the effects of activation of the β_1_-adrenergic signaling system on I_Kto,f_ in cardiac cells [109]. Experimental data of Gonzalez de la Fuente et al. [109] shows clear inhibition of I_to1_ by isoproterenol (0.1 µM) in human left and right atrial myocytes by about 40–50%. Similar inhibition is obtained in neural cells [110] for A-type K^+^ current, I_A_, which is also encoded by *Shal*-type K^+^ channels (Kv4.2), after application of 8-br-cAMP (100 µM), and Schwann cells [111] after application of bradykinin (5 µM), forskolin (1 µM), or db-cAMP (10 µM). Our simulation data shows about a two-fold decrease in peak I_Kto,f_ at saturating concentrations of isoproterenol (Fig. 13E).

Figure 13D shows experimental time courses of I_A_-current inhibition by forskolin (1 µM) or db-cAMP (10 µM) obtained in Schwann cells [111] (filled and unfilled circles, respectively). Our simulation, shown by a solid line for the saturating concentration of isoproterenol (10 µM), gives a reasonable agreement with the experimental data. The saturating concentration of isoproterenol was chosen in the simulation to obtain cAMP concentration in the extracaveolae compartment similar to the experimental values and maximum activation of PKA.

Simulated time courses of I_Kto,f_ in control and after application of 10 µM isoproterenol are shown in Fig. 13E by solid and dashed lines, respectively. Calculations demonstrate about a two-fold inhibition of peak current after stimulation of β_1_-ARs. A similar level of inhibition of I_A_ is obtained by activation of PKA with saturating concentrations of 8-br-cAMP (100 µM) in neural cells [110], and I_to1_ after application of saturating concentrations of isoproterenol in human left and right atrial cells [109]. Simulations also demonstrate depolarization shifts in voltage dependences of G/G_max_ (13 mV) and steady-state inactivation (6 mV) for isoproterenol-stimulated compared to control cells (Fig. 13F), which is similar to the experimental data from [110] obtained for neural cells.

#### Time-independent K^+^ current module

While experimental data shows the effects of activation of the β_1_-adrenergic signaling system on the time-independent current I_K1_, such effects are clear only at potentials below −80 mV [112], [113]. Therefore, we did not include modulation of I_K1_ by β_1_-ARs in our model.

#### Phospholamban module

Phospholamban is the first PKA substrate in the cytosolic compartment, which is included in our model [114]. Its major role is the regulation of SERCA pump activity by changing the affinity of the latter. Activation of the β_1_-adrenergic signaling system leads to an increased phosphorylation of PLB by PKA, resulting in an increase in the SERCA pumping rate of Ca^2+^ from the cytosol to the SR. Dephosphorylation of PLB occurs by protein phosphatase 1 only [59], [115].

**Figure 14:**
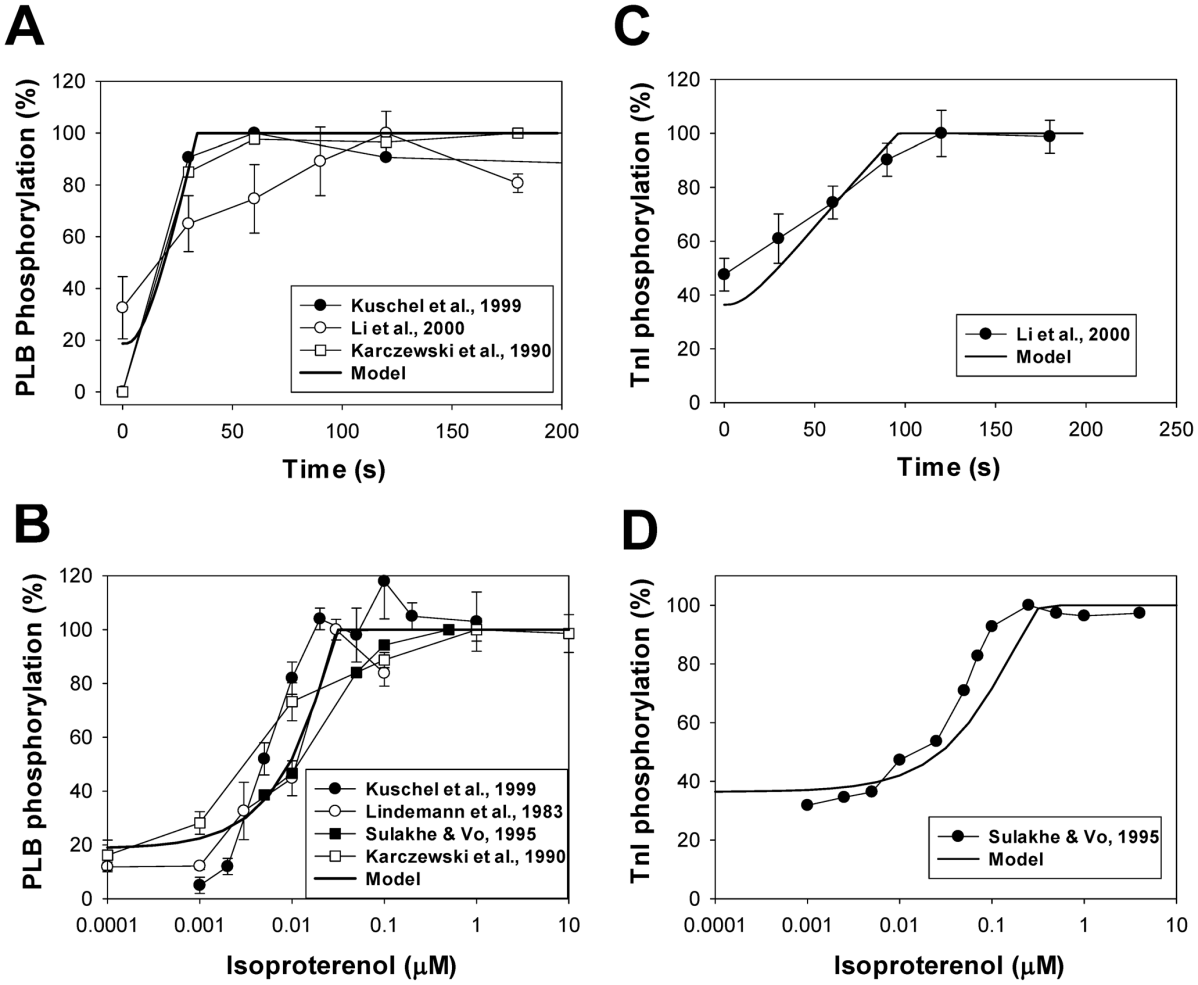
The effects of β_1_-adrenoceptor stimulation on phospholamban and troponin I. **Panel A:** Experimental time courses of the phospholamban phosphorylation (in %) obtained from rat hearts [116], [117] and mouse ventricular myocytes [118]. Data Karczewski et al. [116] are obtained with application of 0.5 µM PKA catalytic subunit; data Kuschel et al. [117] and Li et al. [118] are obtained with application of 1 µM isoproterenol. Simulated time course of PLB phosphorylation is shown by a solid line. **Panel B:** PLB phosphorylation (in %) as functions of isoproterenol concentration. Experimental data from rat hearts [116], [117] were obtained after 2-minute exposure to isoproterenol; data from guinea pig hearts [119] were obtained after 1-minute exposure to isoproterenol; and data from rat ventricular myocytes [120] were obtained after 3-minute exposure to isoproterenol. Simulated data after 2-minute exposure to different concentrations of isoproterenol is shown by a solid line. **Panel C:** Experimental time courses of the troponin I phosphorylation (in %) obtained from mouse ventricular myocytes [118] after application of 1 µM isoproterenol. Simulated time course of troponin I phosphorylation is shown by a solid line. **Panel D:** Troponin I phosphorylation (in %) as functions of isoproterenol concentration. Experimental data from rat ventricular myocytes [120] were obtained after 3-minute exposure to isoproterenol. Simulated data after 2-minute exposure to different concentrations of isoproterenol is shown by a solid line.

Figure 14A shows experimental and simulated time courses of PLB phosphorylation (in %) after stimulation of the β_1_-adrenergic signaling system. Experimental data were obtained from rat hearts by application of 0.5 µM PKA catalytic subunit (unfilled squares, [116]) or 1 µM isoproterenol (filled circles, [117]). Data of Li et al. [118] obtained from mouse ventricular myocytes with application of 1 µM isoproterenol is shown by unfilled circles. Phosphorylation occurs rapidly, within one to two minutes of exposure to agonists. The time course is reasonably well reproduced by the model (solid line in Fig. 14A).

Relative phosphorylation levels of PLB after stimulation with different concentrations of isoproterenol are shown in Fig. 14B. Experimental data obtained from rat [116], [117] and guinea pig [119] hearts and from rat ventricular myocytes [120] consistently show similar half-activation isoproterenol concentration in the range from 0.003 to 0.01 µM. A similar dependence of PLB phosphorylation levels is obtained from the model after 2-minute exposure to different concentrations of isoproterenol (solid line in Fig. 14B).

#### Troponin I module

Troponin I (inhibitory subunit of troponin complex) is the second substrate that is phosphorylated by PKA in the cytosolic compartment [27]. Phosphorylation of troponin I increases the Ca^2+^ unbinding rate by 50% [121], thereby decreasing the corresponding dissociation constant. Dephosphorylation of troponin I occurs by protein phosphatase 2A [61], [115].

Figure 14C shows experimental and simulated data of the time course of the relative phosphorylation level of troponin I for mouse ventricular myocytes. Experimental data were obtained by Li et al. [118] upon stimulation of cardiac cells with 1 µM isoproterenol. Both experimental and simulated data demonstrate a significant phosphorylation level of TnI without stimulation of the β_1_-adrenergic signaling system. Upon application of isoproterenol, both experimental data and simulations show saturating TnI phosphorylation levels within 2 minutes of stimulation (Fig. 14C).

Dependence of the relative phosphorylation level of TnI as a function of isoproterenol concentration is shown in Fig. 14D. Both our simulations and experimental data obtained by Sulakhe and Vo [120] show a significant phosphorylation level of TnI at very low concentrations of isoproterenol. Data also demonstrates that 0.1 µM isoproterenol causes almost complete TnI phosphorylation.

### Method of Simulation

The resulting model contains 141 ordinary differential equations solved by a fourth-order Runge-Kutta method, with different time steps. A relatively small time step of 0.000002 ms is used during the 10 milliseconds after the initiation of the stimulus current; the rest of the time, the time step is 0.0001 ms. Such small time steps are mainly determined by the very fast activation time constants of ryanodine receptors [25], [122]. For simulation of the cellular behavior without electrical stimulation the time step of 0.1 ms is used. The model is implemented as a program code in FORTRAN 90, which runs on a single processor under SUSE Linux 11 on a Dell Precision Workstation T3500 with six-core Intel Xeon CPU W3670 (3.2 GHz, 12 GB RAM). Simulation of one second of the activity of an electrically stimulated cell runs approximately 3 minutes on this workstation. All model equations, model parameters, and initial conditions are given in Appendix S1. The model is developed for a room temperature of 25°C (T = 298°K). Steady-state initial conditions were obtained by running the model equations without electrical stimulations until changes in each variable did not exceed 0.01%. To generate action potentials, a stimulus current, I_stim_, was applied (I_stim_ = 80 pA/pF, τ_stim_ = 1 ms) with the frequencies from 1 to 5 Hz (electrical stimulation). Voltage-clamp protocols for ionic currents are described in corresponding figure legends.

## Results

In this paper, our model for action potential and Ca^2+^ dynamics in an apical mouse cardiac cell [25], [26] was extended to include a β_1_-adrenergic signaling system by using, in considerable part, the methodology from the previously published models [8], [10], [11], [12], [13]. Unlike models [10], [11], [12], our model represents a compartmentalized β_1_-adrenergic signaling system. While the model of Hejiman et al. [8] also includes a compartmentalized β_1_-adrenergic signaling system, it is developed for larger species (dog) and is verified with a different set of experimental data. In particular, our model differs from the Hejiman et al. model [8] in the following: 1) our model was verified in significant part by the experimental data from mice; 2) the model was developed for a different species (mouse) and includes a significantly different set of ionic currents; 3) the model considers non-zero phosphorylation levels of the protein kinase A substrates before activation of the β_1_-adrenergic signaling system; 4) the model includes additional modulation of adenylyl cyclases by βγ-subunits of stimulatory G-protein, G_s_; 5) the model data is compared in significant part with both absolute and relative magnitudes of the activities of major signaling molecules of the β_1_-adrenergic signaling system; 6) the model includes two subpopulations of the L-type Ca^2+^ channels located both in the caveolae and extracaveolae compartments; 7) ryanodine receptors are localized in the extracaveolae compartment. Our model also differs from the recently published Yang and Saucerman model [12] for mouse ventricular myocytes in the following: 1) compartmentalization of β_1_-adrenergic signaling system; 2) inclusion of different types of adenylyl cyclases (AC4–7) and phosphodiesterases (PDE2–4), their compartmentalization and specific regulation by drugs (e.g., rolipram, Ro 20–1724, cilostamide, milrione); 3) multicompartmental distribution of protein kinase A targets and the possibility of their separate regulation by drugs; 4) the effects of β_1_-aderenergic signaling system not only on Ca^2+^ dynamics, but also on action potential.

### cAMP Dynamics During Activation of β_1_-adrenergic Signaling System: the Effects of Phosphodiesterases

First, we investigated the behavior of cAMP and PKA catalytic subunit concentrations in different cellular compartments in response to the activation of the β_1_-adrenergic signaling system with isoproterenol. In non-stimulated cells, cAMP concentrations in caveolae, extracaveolae, and cytosol are equal to 0.2534 µM, 0.5079 µM, and 0.4078 µM, respectively. Such steady state concentrations are determined by the balance between cAMP production by adenylyl cyclases, cAMP degradation by phosphodiesterases, and cAMP fluxes between the compartments. The steady-state concentrations of cAMP were obtained by running the model equations without electrical stimulations until changes in each variable did not exceed 0.01%. The steady-state distribution of cAMP concentrations are qualitatively similar to the values obtained by Iancu et al. [13] and Heijman et al. [8]. Iancu et al. [13] data gives 0.1 µM and 1 µM of caveolar and cellular cAMP concentrations; Heijman et al. [8] data shows 0.3471 µM, 9.6236 µM, and 0.47408 µM of cAMP in the caveolae, extracaveolae, and cytosolic compartments, respectively. All simulated cellular cAMP concentrations are close to the resting cellular cAMP concentrations (∼ 0.5–1 µM) obtained experimentally [52],[53].

**Figure 15:**
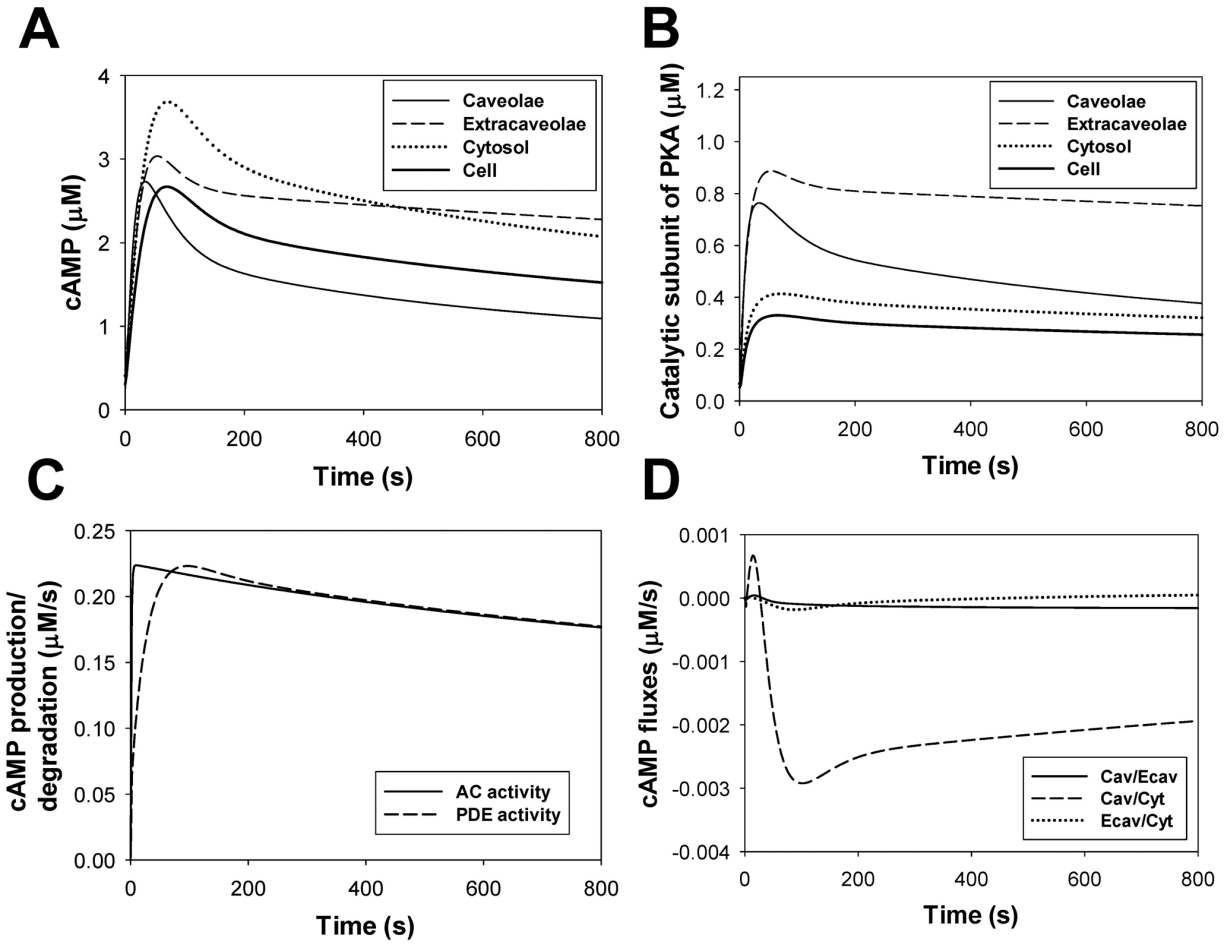
The effects of β_1_-adrenoceptor stimulation on the dynamics of cAMP and PKA. **Panel A:** Simulated time courses of cAMP concentrations in caveolae (thin solid line), extracaveolae (dashed line), and cytosolic compartments (dotted line), and in the whole cell volume (bold solid line) after application of 1 µM isoproterenol. **Panel B:** Simulated time courses of PKA catalytic subunit concentrations in caveolae (thin solid line), extracaveolae (dashed line), and cytosolic compartments (dotted line), and in the whole cell volume (bold solid line) after application of 1 µM isoproterenol. **Panel C:** Simulated time courses of cAMP production rate by adenylyl cyclases (solid line) and cAMP degradation rate by phosphodiesterases (dashed line) after application of 1 µM isoproterenol. **Panel D:** Simulated time course of cAMP fluxes between caveolae and extracaveolae (solid line), caveolae and cytosol (dashed line), and extracaveolae and cytosol (dotted line) after application of 1 µM isoproterenol. Fluxes are normalized to the cell volume *V^cell^*.

Figure 15A shows the simulated time courses of cAMP concentrations in different subcellular compartments in response to 1 µM isoproterenol. All cAMP transients show relatively fast rising (time-to peak is 35–70 s) and initial decaying phases due to the activation of ACs and PDEs (Fig. 15C), and relatively slow decay due to desensitization of β_1_-ARs. The fastest cAMP transient is observed in the caveolae compartment because of the larger relative activities of ACs and PDEs in this compartment (see Appendix S1). In caveolae, cAMP also decays to the smaller quasi-steady-state values during desensitization of β_1_-ARs. In the extracaveolae compartment, cAMP rises and decays somewhat slower than in the caveolae compartment and approaches the largest quasi-steady-state values at later times. cAMP transient in the cytosolic compartment has the largest time-to-peak value and the largest amplitude. It predominantly determines the behavior of cAMP in the cardiac cell (thick solid line). Note that the cellular cAMP concentration transient has a somewhat smaller amplitude due to its normalization to the whole cell volume *V^cell^*.

Figures 15C and 15D demonstrate major factors that affect cAMP transient development. cAMP increases upon relatively fast activation of adenylyl cyclases by the alpha subunit of the stimulatory G protein (G_sα_) in all subcellular compartments (time-to-peak is ∼10 s). This process is accompanied by activation of phosphodiesterases, with some delay, due to the rising cAMP concentration and phosphorylation of PDEs by catalytic subunits of PKA. The interaction between ACs and PDEs reaches quasi-steady-state by ∼200 s, when their activities become close to each other. The slower part of cAMP transient is determined by the desensitization of β_1_-ARs when the concentration of G_sα_ decreases due to its smaller production and faster re-association with G_sβγ_ to restore G_sαβγ_. In addition to the interaction of ACs and PDEs, cAMP fluxes between the subcellular compartments contribute to cAMP transients (Fig. 15D). At early times, up to ∼30 s, the largest flux is from the caveolae to the cytosolic compartment, which is determined by the largest rising rate of cAMP in caveolae. Then this flux changes direction, and a significant amount of cAMP flows from cytosol to caveolae (dashed line in Fig. 15D). Smaller fluxes, from extracaveolae to caveolae and from cytosol to extracaveolae, are also observed during this stage of activation of β_1_-ARs.

In our model, in contrast to cAMP, PKA does not move between the compartments. Its activation is determined by the type of PKA (PKAI or PKAII) and the level of cAMP in each compartment (Fig. 15B). Concentrations of catalytic subunits of PKA decay relatively slowly in extracaveolae and cytosolic compartments (dashed and dotted lines in Fig. 15B, respectively) compared to those in caveolae compartment (thin solid line in Fig. 15B), despite the relatively fast decay of cAMP concentrations in both caveolae and cytosol (Fig. 15A). Such relatively fast return of cAMP and PKA concentrations to their resting values in caveolae points to an important role of this compartment in the fast cAMP-mediated signal transduction within the β_1_-adrenergic signaling system.

**Figure 16:**
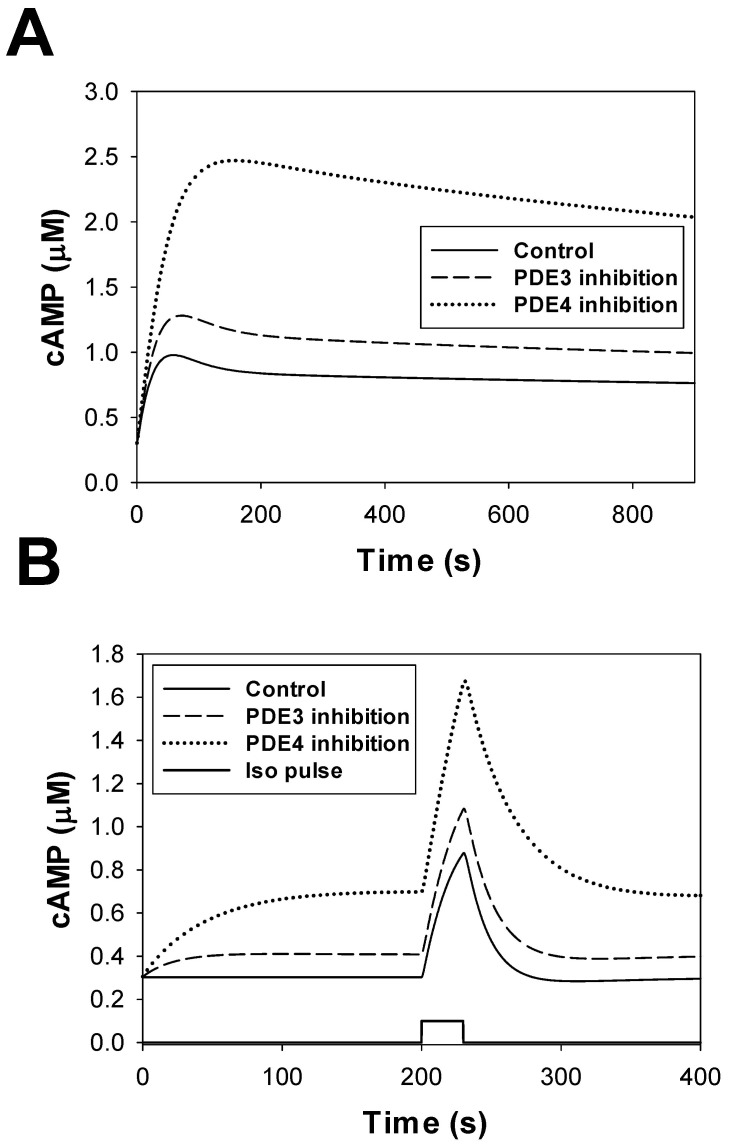
The effects of PDE3 and PDE4 inhibition on cAMP dynamics upon activation of β_1_-adrenergic receptors. **Panel A:** Simulated time courses of cellular cAMP concentrations for control conditions (solid line), upon inhibition of PDE3 (dashed line), and upon inhibition of PDE4 (dotted line) after sustained application of 0.1 µM isoproterenol at time moment *t* = 0 s. Activities of PDE3 or PDE4 are inhibited by 90% to simulate the effects of corresponding selective inhibitors, cilostamide or milrione for PDE3, or rolipam or Ro 20–1724 for PDE4. **Panel B:** Simulated time courses of cellular cAMP concentrations for control conditions (solid line), upon inhibition of PDE3 (dashed line), and upon inhibition of PDE4 (dotted line) after pulsed application of 0.1 µM isoproterenol at time moment *t* = 200 s for 30 s (thick solid line). The degrees of inhibition of PDE3 and PDE4 are the same as in Panel A.

In addition to control conditions, we also simulated the effects of PDE3 and PDE4 inhibition on cAMP dynamics (Fig. 16). As there is no detailed experimental data on cAMP dynamics during PDE inhibition in mouse ventricular myocytes, we compared qualitatively the simulated cellular response to the data obtained for rats [123]. The data for rats shows that the selective inhibition of PDE3 by cilostamide has significantly smaller effects on cAMP transient than the selective inhibition of PDE4 by Ro 20–1724. We simulated two different methods of cAMP transient production during inhibition of PDE3 or PDE4: with continuous and pulsed application of 0.1 µM isoproterenol. Figure 16A shows that continuous application of isoproterenol from *t* = 0 s with simultaneous selective inhibition of PDE3 (90% inhibition) leads to an increase in peak cAMP transient by about 30%. In contrast, simultaneous application of isoproterenol and selective inhibition of PDE4 (90% inhibition) increases peak cAMP value by about ∼150%. This result points to the primary role of PDE4 in cAMP degradation compared to PDE3. Similar results were obtained experimentally in rats [123], [124]. It needs to be determined experimentally whether such predicted effect will be observed in mouse ventricular myocytes as well.

In another set of simulations, the cells were first affected by the selective inhibitor of PDE3 (or PDE4), and then a pulse of 0.1 µM isoproterenol was applied at *t* = 200 s for 30 s (thick solid line in Fig. 16B). In the time interval from 0 to 200 s, the selective inhibition of PDE3 or PDE4 (both by 90%) increases steady-state cellular cAMP concentrations by ∼35% and ∼130% (Fig. 16B). Pulsed application of isoproterenol produces relatively short cAMP transients, which decay to the pre-pulse quasi-steady-state levels. When PDE3 is selectively inhibited, cAMP transient amplitude is equal to 0.6754 µM, which is ∼17% larger than the cAMP transient in control (0.5763 µM). Selective inhibition of PDE4 results in the larger cAMP transient of 0.9780 µM, or ∼69.7% larger than in control. Experimental data with pulsed application of isoproterenol and selective inhibition of PDE3 and PDE4 obtained from rat ventricular myocytes show qualitatively similar behavior [123]. Further experiments are necessary to determine whether similar effects can be observed in mice.

### The Effects of Activation of the β_1_-adrenergic Signaling System on Mouse Action Potential

**Figure 17:**
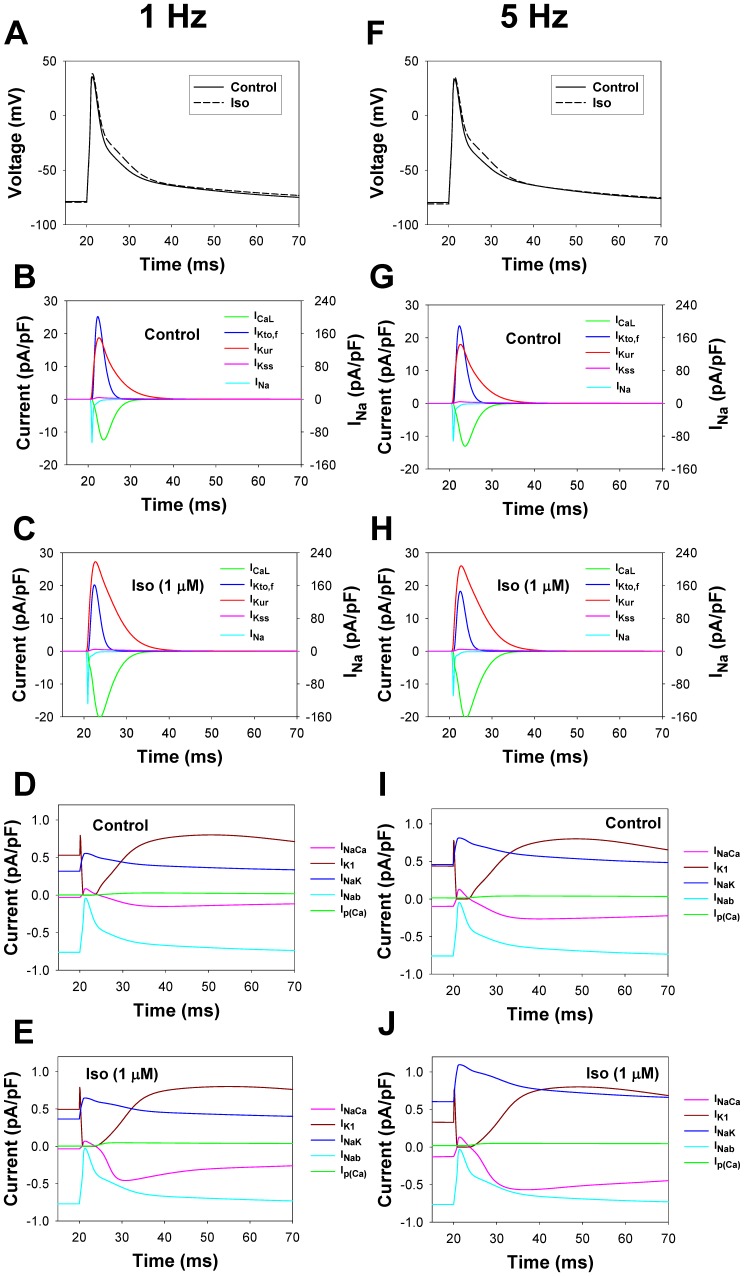
Simulated action potentials and underlying ionic currents of the isolated ventricular cell model. Simulated action potentials and underlying ionic currents are shown for control conditions and after application of 1 µM isoproterenol at two pacing frequencies 1 Hz (**Panels A–E**) and 5 Hz (**Panels F–J**) (I_stim_ = 80 pA/pF, τ_stim_ = 1.0 ms). **Panels A and F:** Simulated action potentials for control conditions (solid line) and after isoproterenol application (dashed line). **Panels B, D, G, and I:** Currents underlying the AP for control condition. **Panel C, E, H, and J:** Currents underlying the AP after isoproterenol application. The scales for the relatively large fast Na^+^ current, I_Na_, are given on the right axis in **Panels B, C, G and H**. APs and ionic currents are shown after 300 s stimulation. In **Panels A, C, E, F, H, and J** 1 µM isoproterenol is applied at time *t* = 0 s.

Experimental data obtained from mouse ventricular myocytes shows that the activation of the β_1_-adrenergic signaling system affects action potential shape and duration [75], [125], [126]. To simulate these effects, we stimulated the model cell with current pulses (I_stim_ = 80 pA/pF, τ_stim_ = 1.0 ms) at frequencies 1 and 5 Hz for 300 s without and with application of 1 µM isoproterenol. Figure 17A shows corresponding action potentials obtained by 1 Hz stimulation. Simulated data shows that isoproterenol prolongs mouse action potential at different degrees of repolarization. Table 1 shows the comparison of the simulated prolongations of the action potentials with the experimental data. For example, APD_50_ and APD_90_ are prolonged by 21.67% and 14.90%, respectively. The simulated data for APD_50_ prolongation is close to the corresponding experimental data, 13.5±4% [126] and 18% [125] prolongation, but somewhat smaller than 48% prolongation obtained by Wang et al. [75]. In addition, the simulated data for APD_90_ prolongation is within the experimental data variation (5±7% [126], 9% [125], and 51% [75]).

**Table 1:** Simulated and experimental prolongations (in %) of the action potentials in mouse ventricular myocytes at different repolarizations and stimulation frequencies (1 µM Iso).

	APD_25_, ms	APD_50_, ms	APD_75_, ms	APD_90_, ms
		Experimental values		
Wu et al. [126] *		13.5±4		5±7
Tong et al. [125] **		18		9
Tong et al. [125] ***		38		28
Wang et al. [75] ****		48		51
		Simulated values (this paper)		
1 Hz	7.65	21.67	19.10	14.90
2 Hz	8.09	22.58	17.20	15.95
3 Hz	9.14	24.76	18.77	16.73
4 Hz	9.04	26.09	18.04	13,16
5 Hz	8.33	27.27	17.00	10.37

* Experimental data are obtained at 0.5 Hz stimulation and 2 µM isoproterenol.

** Experimental data are obtained at 2 Hz stimulation.

*** Experimental data are obtained at 4 Hz stimulation.

**** Experimental data are obtained at 1 Hz stimulation.

It is remarkable that such relatively small AP prolongation due to activation of β_1_-ARs is accompanied by significant changes in underlying ionic currents (Fig. 17). Simulations elucidate the mechanism of this prolongation. The four major currents are responsible for the changes in AP shape at an early stage of repolarization. The fast Na^+^ current, I_Na_, changes only slightly, which leads to a small increase in AP amplitude (from 114.3 mV in control to 117.8 mV after Iso). The L-type Ca^2+^ current, I_CaL_, increases almost twice upon stimulation of β_1_-ARs, and promotes AP prolongation. There is also a decrease in the amplitude of the rapidly recovering transient outward K^+^ current, I_Kto,f_, which also contributes to AP prolongation. An increase in the amplitude of the ultrarapidly activating K^+^ current, I_Kur_, is the major opposing effect to AP prolongation, resulting in a relatively small total effect. At 90% of repolarization, four other, relatively small currents contribute to repolarization: the Na^+^/Ca^2+^ exchanger current, I_NaCa_, the time-independent K^+^ current, I_K1_, the Na^+^/K^+^ pump, I_NaK_, and the Na^+^ background current, I_Nab_. (Fig. 17, D and E). Significant increase in the magnitude of I_NaCa_ current upon application of isoproterenol (Fig. 17E) is one of the additional factors that cause action potential prolongation at 90% repolarization.

Simulation data obtained at stimulation frequency 5 Hz (Fig. 17, F–J) shows very similar behavior to the data for 1 Hz. The only significant change is in the peak amplitudes of the fast Na^+^ current, I_Na_, which are decreased during stimulation with 5 Hz. A smaller decrease is observed for K^+^ currents I_Kto,f_ and I_Kur_ at 5-Hz stimulations. Simulation of application of 1 µM isoproterenol at 5 Hz stimulation prolongs APD_50_ and APD_90_ by 27.27% and 10.37%, respectively (Table 1). These prolongations are compared to the experimental data of Tong et al. [125] obtained at 4 Hz stimulation (38% and 28% prolongation for APD_50_ and APD_90_, respectively). Simulation data shows a correct trend of APD_50_ prolongation, which increases with stimulation frequency. However, simulated prolongation of APD_90_ at 5 Hz-stimulation shows somewhat smaller magnitude compared to 4 Hz-data of Tong et al. [125].

We have also simulated the effects of isoproterenol on action potential durations (APDs) at the frequencies ranged from 1 to 5 Hz. Simulation data shows relatively small increase in APDs at different levels of repolarization with stimulation frequency. For example, without isoproterenol, APD_50_ changes from 3.0 to 3.3 ms and APD_90_ changes from 25.5 to 27.0 ms when stimulation frequency increases from 1 to 5 Hz. Upon exposure to isoproterenol, APD_50_ increases from 3.65 to 4.2 ms and APD_90_ ranges from 29.3 to 30.7 ms during the same increase in stimulation frequency. Prolongation of cardiac action potential durations upon application of isoproterenol is consistently observed experimentally in mouse ventricular myocytes [75], [125], [126] (Table 1).

### The Effects of Activation of the β_1_-adrenergic Signaling System on Ca^2+^ Dynamics

**Figure 18:**
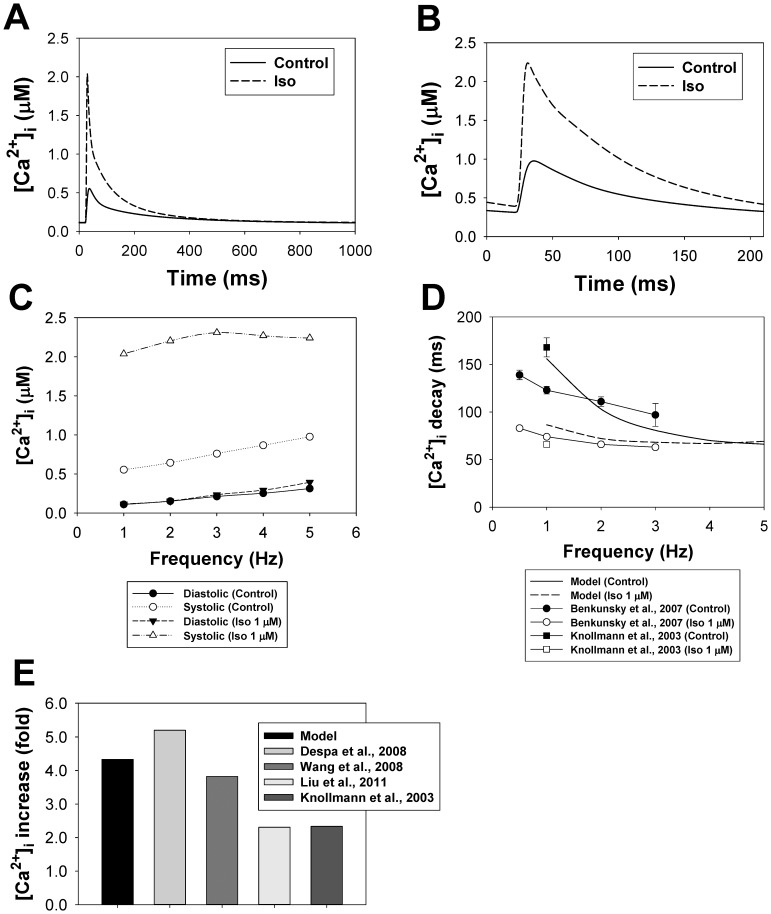
Simulated and experimental [Ca^2+^]_i_ transients and their characteristics in an isolated ventricular cell for control conditions and after application of isoproterenol. **Panel A:** Simulated [Ca^2+^]_i_ transients for control conditions (solid line) and after 1 µM isoproterenol application (dashed line) obtained with 1 Hz-stimulation. **Panel B:** Simulated [Ca^2+^]_i_ transients for control conditions (solid line) and after 1 µM isoproterenol application (dashed line) obtained with 5 Hz-stimulation. **Panel C:** Simulated diastolic (filled symbols) and systolic (unfilled symbols) [Ca^2+^]_i_ magnitudes for control conditions (circles) and after 1 µM isoproterenol application (triangles) as functions of stimulation frequency. **Panel D:** Decay time constants for intracellular [Ca^2+^]_i_ transients for control conditions and after 1 µM isoproterenol application as functions of stimulation frequency. Simulated data are shown by solid (control) and dashed (1 µM isoproterenol) lines. The lines with symbols show experimental data from Benkunsky et al. [131]; experimental data from Knollmann et al. [128] are shown by squares. **Panel E:** Increase in [Ca^2+^]_i_ transient amplitudes after application of isoproterenol (folds). Model data and data of Wang et al. [75] and Liu et al. [129] are obtained at 1 Hz and 1 µM isoproterenol; data of Despa et al. [127] are obtained at 2 Hz and 1 µM isoproterenol; data of Knollmann et al. [128] are obtained at 1 Hz and 0.5 µM isoproterenol. All simulation data in this figure are obtained after 300-s stimulations and exposure to 1 µM isoproterenol.

Experimental data obtained with micromolar concentrations of isoproterenol demonstrates significant (up to 5-fold) increase in intracellular [Ca^2+^]_i_ transients obtained from mouse ventricular myocytes (Fig. 18, [75], [127], [128], [129]). Our simulations are able to reproduce this behavior. Figure 18, A and B, shows simulated [Ca^2+^]_i_ transients, obtained without and with 1 µM isoproterenol at stimulation frequencies 1 and 5 Hz. Stimulation of the β_1_-adrenergic signaling system increases the simulated amplitudes of [Ca^2+^]_i_ at 1 Hz-stimulation from 0.44 µM to 1.92 µM (Fig. 18A), or by 4.33 fold. At larger stimulation frequency 5 Hz, [Ca^2+^]_i_ increases from 0.66 µM to 1.85 µM (Fig. 18B), or by 2.79 fold. These values are in good agreement with the experimental data [75], [127], [128], [129], which ranges from 2.3 to 5.2-folds increase. Frequency dependence of the diastolic and systolic [Ca^2+^]_i_ (Fig. 18C) demonstrates an increase of their magnitudes, with the exclusion of peak [Ca^2+^]_i_ at 1 µM isoproterenol and stimulation frequencies 4 and 5 Hz. It is remarkable that the model reproduces experimentally found feature such as independence of diastolic [Ca^2+^]_i_ from the β_1_-adrenergic stimulation [128], [130].

Our model also provides appropriate time constants for the decay of intracellular [Ca^2+^]_i_ and corresponding acceleration of the decay upon application of isoproterenol. Simulated and experimental frequency dependences of the decay of intracellular [Ca^2+^]_i_ without and with 1 µM isoproterenol are shown in Fig. 18D. The simulated time constants for the decay decrease with the stimulation frequency without and with isoproterenol and reasonably well approximate experimental data [128], [131]. Note, however, that the absolute values of [Ca^2+^]_i_ relaxation constants obtained by Wang et al. [75] without and with isoproterenol are significantly longer, 267 and 147 ms, respectively, but their ratio (∼1.8) is very close to the corresponding simulated value.

**Figure 19:**
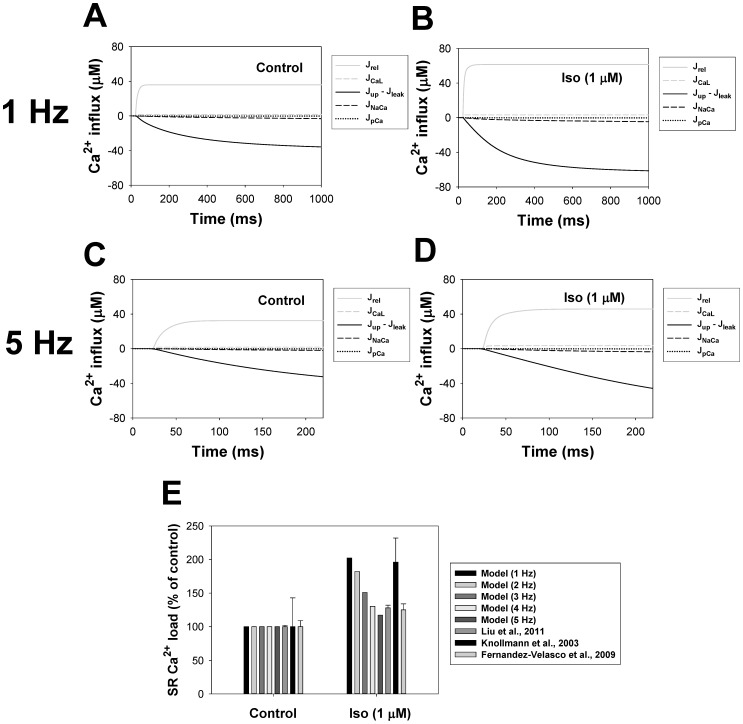
Ca^2+^ fluxes and the SR Ca^2+^ load. Simulated major integral Ca^2+^ fluxes during one cardiac cycle in the isolated ventricular cell model for control conditions (**Panels A and C**) and after application of 1 µM isoproterenol (**Panels B and D**). Pacing frequencies are 1 Hz (**Panels A and B**) and 5 Hz (**Panels C and D**). Major integral Ca^2+^ fluxes are shown after 300 s of stimulation. In **Panels B and D** 1 µM isoproterenol is applied at time *t* = 0 s. Here, J_CaL_ is the Ca^2+^ entering the cell through L-type Ca^2+^ channels; J_up_ – J_leak_ is the uptake Ca^2+^ from the cytosol to the network SR with subtracted Ca^2+^ leak from the SR to the cytosol; J_NaCa_ is the Ca^2+^ outflux from the cytosol through the Na^+^/Ca^2+^ exchanger; and J_pCa_ is the Ca^2+^ outflux through the sarcolemmal Ca^2+^ pump. **Panel E:** Increase in the SR Ca^2+^ load after 300-s application of isoproterenol. Model data are shown for stimulation frequencies 1, 2, 3, 4, and 5 Hz; experimental data of Liu et al. [129] and Fernandez-Velasco et al. [132] are obtained at 1 µM isoproterenol; data of Knollmann et al. [128] are obtained at 0.5 µM isoproterenol.

Our model allows for evaluation of various Ca^2+^ fluxes and their modifications by isoproterenol. Figure 19 shows simulated Ca^2+^ fluxes obtained at 1 and 5 Hz stimulations without and with 1 µM isoproterenol. Simulations show that the activation of the β_1_-adrenergic signaling system leads to a significant increase in Ca^2+^ influx through the L-type Ca^2+^ channels during action potential from 1.6 µM (4.5% of released Ca^2+^ from the SR) to 3.3 µM (5.3%) at 1 Hz and from 1.8 µM (5.5%) to 3.5 µM (7.6%) at 5 Hz during cardiac cycles. Increase in Ca^2+^ influx results in an increase in the SR Ca^2+^ load, from 919 µM to 1860 µM at 1 Hz (Fig. 19E, an increase which is comparable to the experimental findings [128], [129], [132]), resulting in a growth of Ca^2+^-induced Ca^2+^ release from 36 µM to 61 µM. Smaller increase in the SR Ca^2+^ load is obtained at 5 Hz, from 1367 µM to 1602 µM, which results in an increase of Ca^2+^ release from 32 µM to 46 µM. An increase in intracellular [Ca^2+^]_i_ transients after application of isoproterenol also increases Ca^2+^ extrusion from the cytosol by the Na^+^/Ca^2+^ exchanger from 3.1 µM (8.6%) to 4.6 µM (7.6%) at 1 Hz and from 1.9 µM (6.0%) to 3.6 µM (7.8%) at 5 Hz; however, this increase does not completely compensate for the Ca^2+^ entry into the cell, which ultimately leads to an increase in the SR Ca^2+^ load. Only 0.18 µM and 0.28 µM (1 Hz stimulation) and 0.16 µM and 0.22 µM (5 Hz stimulation) of Ca^2+^ are extruded from the cell by a slow mechanism through the sarcolemmal Ca^2+^ pump for control conditions and after application of isoproterenol, respectively, which is about 0.5% of the released Ca^2+^ in all cases. Our model also includes background Ca^2+^ influx of ∼1.7 µM (1 Hz) and ∼0.3 µM (5 Hz) due to a very large Ca^2+^ gradient across the membrane, which does not change upon stimulation of β_1_-ARs.

**Figure 20:**
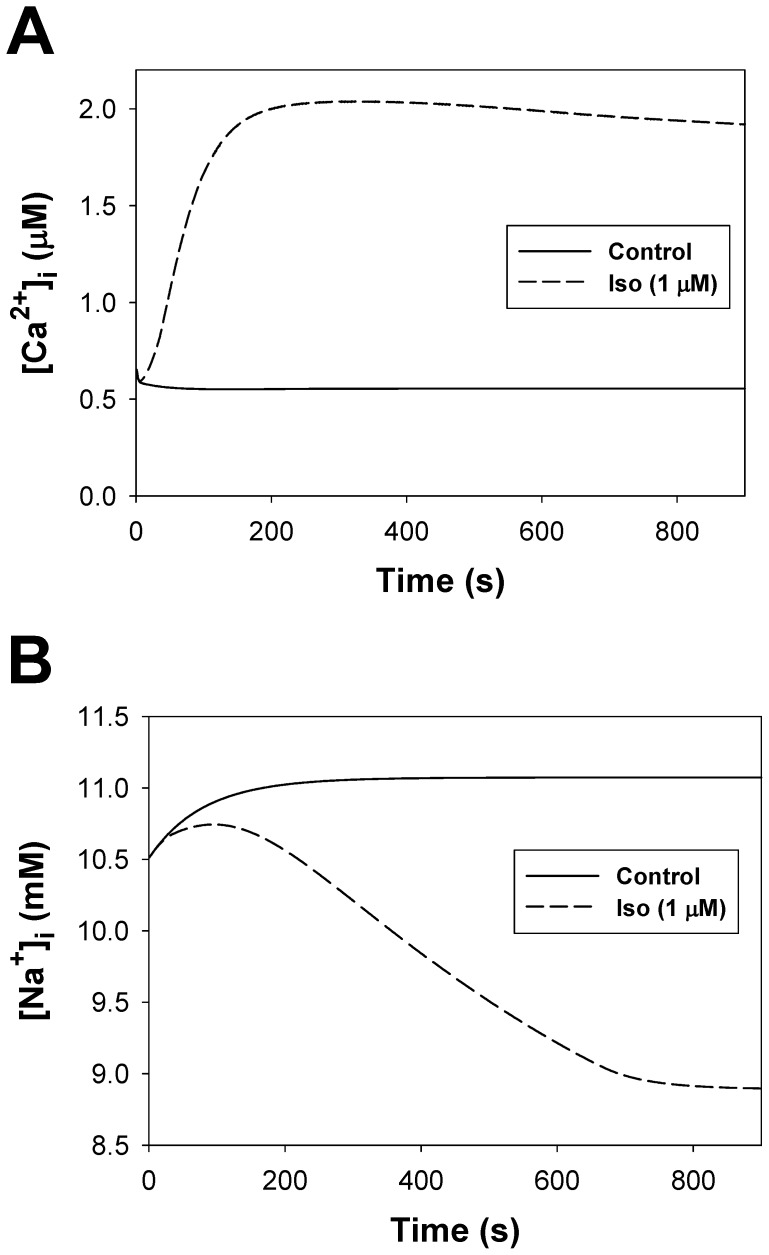
Dynamics of [Ca^2+^]_i_ transients and [Na^+^]_i_ concentrations upon stimulation of β_1_-adrenoceptors. **Panel A:** Simulated peak values of [Ca^2+^]_i_ transient as function of time for control conditions (solid line) and after 1 µM isoproterenol application at *t* = 0 s (dashed line). **Panel B:** Simulated intracellular [Na^+^]_i_ concentrations as function of time for control conditions (solid line) and after 1 µM isoproterenol application at *t* = 0 s (dashed line). In both panels, stimulation frequency is 1 Hz.

Figure 20 shows the time behavior of the peak values of intracellular [Ca^2+^]_i_ transients and intracellular [Na^+^]_i_ concentration for control conditions and after application of 1 µM isoproterenol upon stimulation with frequency 1 Hz. In control, [Ca^2+^]_i_ transients approach quasi-steady-state values after a short-time initial decay (negative staircase effect). After application of isoproterenol, [Ca^2+^]_i_ transients relatively rapidly increase in amplitude during the first ∼300 seconds, and then they slowly decay. Such behavior of [Ca^2+^]_i_ transients is similar to the experimental data obtained by Despa et al. [127]. Initial increase in [Ca^2+^]_i_ transients is due to the rapid activation of β_1_-adrenoceptors and cAMP accumulation; relatively slow decay of peak [Ca^2+^]_i_ is due to β_1_-adrenoceptors desensitization. In addition, Despa et al. [127] have shown that the stimulation of β_1_-ARs decreases intracellular [Na^+^]_i_ concentration due to an increased function of the Na^+^-K^+^ pump. Our simulation data reproduced this behavior. In control, stimulation of the model cell leads to a slight increase in [Na^+^]_i_ to saturating values (solid line in Fig. 20B). However, the behavior of [Na^+^]_i_ changes after application of isoproterenol (dashed line in Fig. 20B): after a small initial increase until peak value, [Na^+^]_i_ decreases subsequently in time until quasi-steady-state level. Simulated [Na^+^]_i_ concentration begins to decay at approximately the same time as [Ca^2+^]_i_ achieves its maximum value. Experimental data shows a plateau in concentration of [Na^+^]_i_ for a short time after application of isoproterenol, which is replaced by a decay after [Ca^2+^]_i_ achieves the maximum value [127].

**Figure 21:**
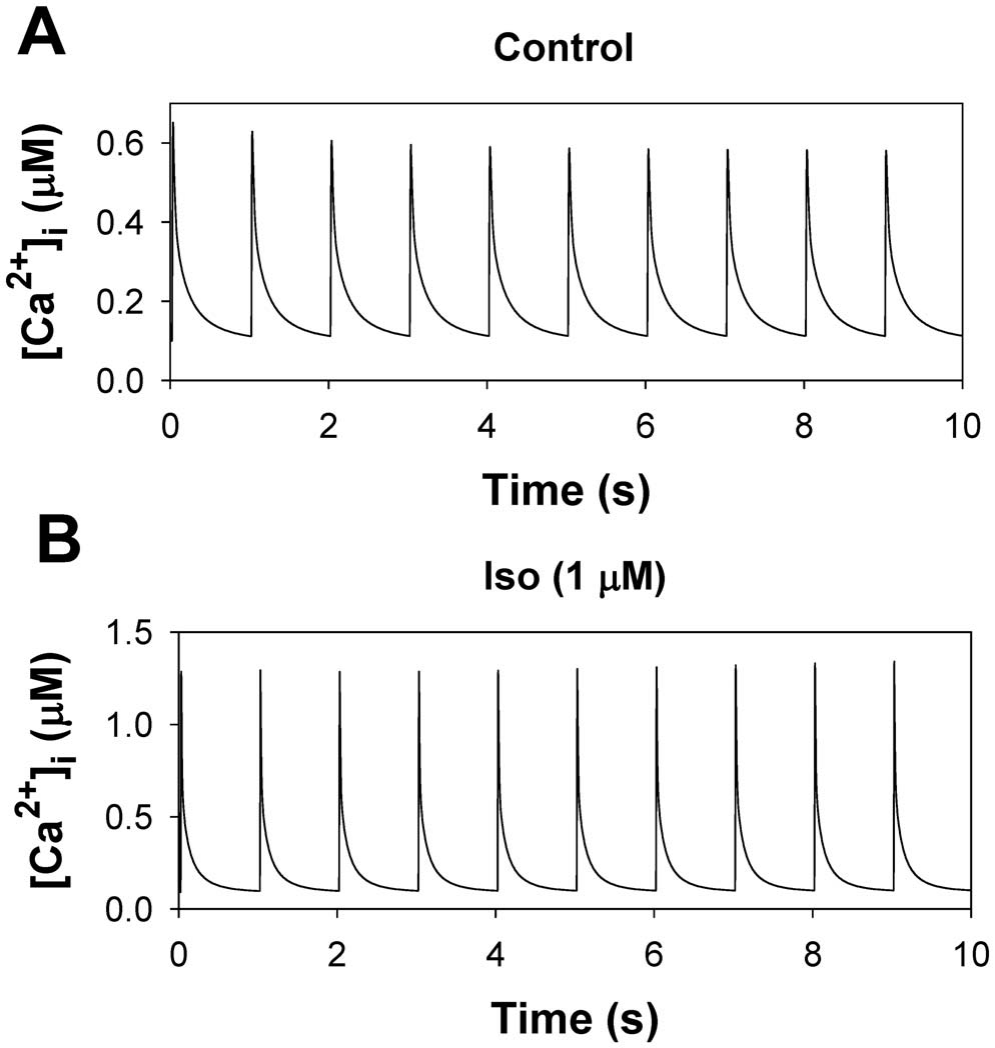
The effects of isoproterenol on negative staircase of [Ca^2+^]_i_ transients. **Panel A:** Simulated [Ca^2+^]_i_ transients as function of time for control conditions show clear negative staircase effect. **Panel B:** Simulated [Ca^2+^]_i_ transients as function of time after 1 µM isoproterenol application at *t* = 0 s does not show negative staircase effect. In both panels, stimulation frequency is 1 Hz.

Finally, experimental data demonstrates the absence of the negative staircase effect in [Ca^2+^]_i_ transients which are elicited by stimulation of a quiescent cell after application of isoproterenol [27]. The experimental data are obtained for rat ventricular myocytes, whose Ca^2+^ handling system is similar to that for mice. Our model for mouse ventricular myocytes reproduced this behavior. Figure 21A shows [Ca^2+^]_i_ transients upon stimulation of the quiescent cell with the frequency of 1 Hz in the control cell. The [Ca^2+^]_i_ decrease in time, clearly shows a negative staircase effect. In another simulation (Fig. 21B), 1 µM isoproterenol is applied to the quiescent cell for 600 seconds, and then the cell is electrically stimulated with the frequency of 1 Hz. The simulation data does not show a decrease in [Ca^2+^]_i_ transient amplitudes, thereby confirming the absence of a negative staircase effect after stimulation of the β_1_-adrenergic signaling system.

The mechanism of two different types of behavior is explained by the analysis of the input and output Ca^2+^ fluxes without and with application of isoproterenol. Our simulations of the case without isoproterenol show that the stimulation of the quiescent myocyte increases total intracellular Ca^2+^ loss and decreases the SR Ca^2+^ load, resulting in continuous decrease in [Ca^2+^]_i_ transient (Fig. 21A). If the simulated cell is first pretreated with isoproterenol in the quiescent state for 600 seconds and then the electrical stimulation is applied, the Ca^2+^ fluxes inside and outside the cell remain balanced due to the predominant increase in the L-type Ca^2+^ current. The result is that the SR Ca^2+^ load is not decreased, resulting in the absence of negative staircase effect.

### The Effects of Activation of the β_1_-adrenergic Signaling System on Na^+^ Fluxes

**Figure 22:**
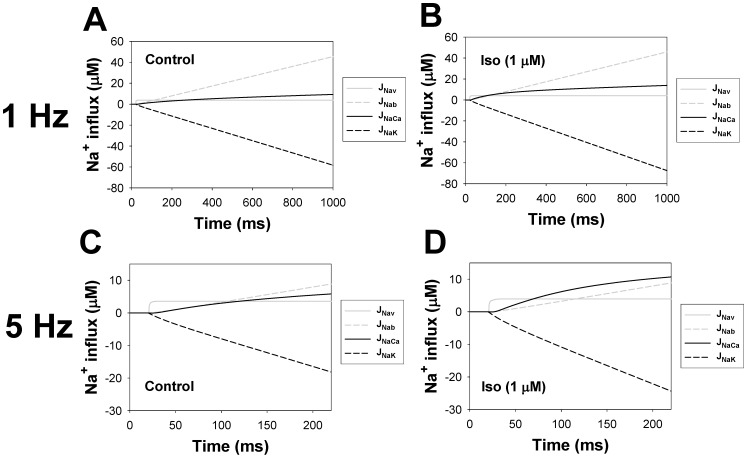
The effects of isoproterenol on integral Na^+^ fluxes. Simulated major integral Na^+^ fluxes during one cardiac cycle in the isolated ventricular cell model are shown for control conditions (**Panels A and C**) and after application of 1 µM isoproterenol (**Panels B and D**). Pacing frequencies are 1 Hz (**Panels A and B**) and 5 Hz (**Panels C and D**). Major integral Na^+^ fluxes are shown after 300 s of stimulation. In **Panels B** **and D** 1 µM isoproterenol is applied at time *t* = 0 s. Here, J_Nav_ is the Na^+^ entering the caveolae compartment through the fast Na^+^ channels; J_Nab_ is the background Na^+^ influx; J_NaCa_ is the Na^+^ influx to the cytosol through the Na^+^/Ca^2+^ exchanger; and J_NaK_ is the Na^+^ outflux through the Na^+^-K^+^ pump.

Experimental data shows the effects of the β_1_-adrenergic signaling system on Na^+^ dynamics in ventricular myocytes [100], [127]. In particular, in mouse ventricular myocytes, [Na^+^]_i_ decreases upon stimulation of β_1_-adrenergic receptors. Such behavior was reproduced by our model as well (Fig. 20B). In addition, our model is able to predict major Na^+^ fluxes into and out of the mouse ventricular myocytes. Simulated Na^+^ fluxes for control conditions and after application of 1 µM isoproterenol for stimulation frequencies of 1 and 5 Hz (I_stim_ = 80 pA/pF, τ_stim_ = 1 ms) are shown in Fig. 22. Na^+^ enters the cell through the fast Na^+^ channels, Na^+^/Ca^2+^ exchanger, and background mechanisms (large Na^+^ gradient and other exchangers and co-transporters [133]), and is pumped out of the cell by the Na^+^-K^+^ pump. Without isoproterenol and at 1 Hz stimulation, 3.8 µM of Na^+^ enter the cell during action potential through the fast Na^+^ channels (6.5% of the total Na^+^ influx), 9.3 µM of Na^+^ through the Na^+^/Ca^2+^ exchanger (16%), and 45.5 µM of Na^+^ through the background mechanism (77.5%), giving total Na^+^ influx of 58.6 µM. After application of 1 µM isoproterenol and 1 Hz stimulation, the total Na^+^ influx into the cell increases to 64.0 µM, resulting in proportional changes of Na^+^ influx through the fast Na^+^ channels (4.2 µM, or 6.5%), increased influx through the Na^+^/Ca^2+^ exchanger (13.9 µM, or 22%), and unchanged Na^+^ influx through the background mechanism (45.9 µM, or 71.5%). At the larger stimulation frequency (5 Hz), the contribution of the different Na^+^ entry mechanisms changes. Without isoproterenol, 3.6 µM of Na^+^ enter the cell during action potential through the fast Na^+^ channels (20% of the total Na^+^ influx), 5.8 µM of Na^+^ through the Na^+^/Ca^2+^ exchanger (32%), and 8.9 µM of Na^+^ through the background mechanism (48%), yielding total Na^+^ influx of 18.3 µM. Application of 1 µM isoproterenol increases the total Na^+^ influx into the cell to 24.4 µM, resulting in an increase of Na^+^ influx through the fast Na^+^ channels (3.9 µM, or 17%) and the Na^+^/Ca^2+^ exchanger (10.7 µM, or 46%), and unchanged Na^+^ influx through the background mechanism (8.9 µM, or 37%).

Thus, our simulations allow for the estimation of Na^+^ fluxes and dynamics, and their modifications by the β_1_-adrenergic signaling system. The model data shows an increased fraction of voltage-dependent Na^+^ entry into the cell at higher stimulation frequencies and the shift of the balance of the Na^+^ fluxes upon application of isoproterenol towards outside the cell due to an increased function of the Na^+^-K^+^ pump.

### The Effects of the Block of Different Populations of I_CaL_ on the Action Potential and [Ca^2+^]_i_ Transients

Experimental data shows that two different populations of the L-type Ca^2+^ channels, in caveolin-3-rich and caveolin-3-free membrane fractions, affects differently [Ca^2+^]_i_ transients and cellular contraction [70]. Such investigations were performed by a specific block of the caveolae-linked L-type Ca^2+^ channels. While the experiments show a 10% decrease in the mean values of the peak of [Ca^2+^]_i_ transients in the myocytes with blocked caveolae-located L-type Ca^2+^ channels compared to control, such decrease did not reach statistical significance. Similar small, but not significant, decrease was also observed in myocyte contraction.

**Figure 23:**
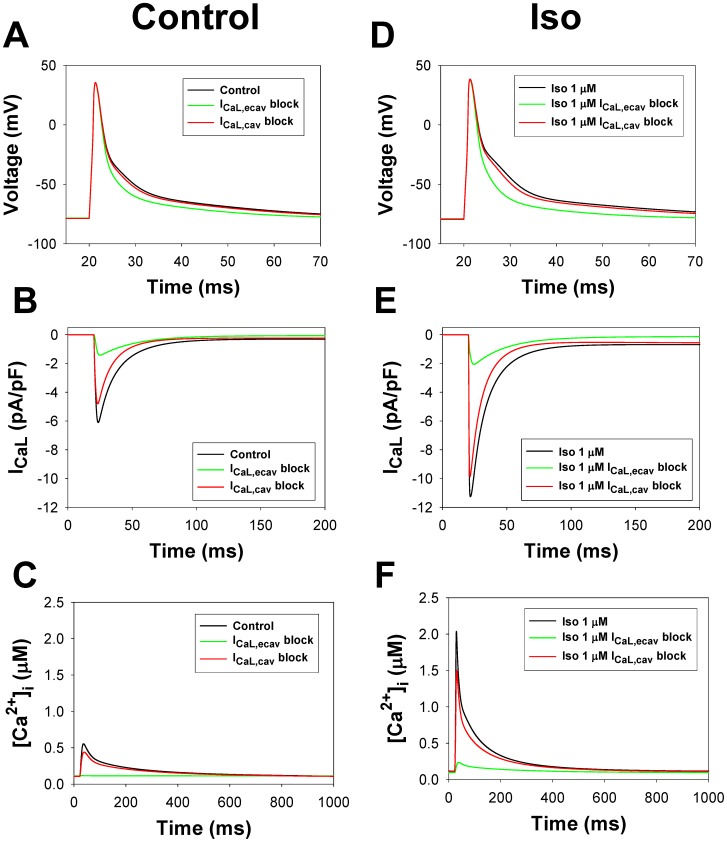
The effects of two subpopulations of the L-type Ca^2+^ current block on the action potentials and [Ca^2+^]_i_ transients. Simulated effects of two subpopulations of the L-type Ca^2+^ current block on the action potentials and [Ca^2+^]_i_ transients in the isolated ventricular cell model are shown for control conditions and after application of 1 µM isoproterenol at pacing frequency 1 Hz (I_stim_ = 80 pA/pF, τ_stim_ = 1.0 ms). **Panels A–C**: Control conditions; **Panels D–F**: Isoproterenol is applied. **Panels A and D:** Simulated action potentials for control conditions (solid black line), after I_CaL,ecav_ block (green line), and after I_CaL,cav_ block (red line). **Panels B and E:** Simulated I_CaL_ currents. **Panel C and F:** Simulated [Ca^2+^]_i_ transients. APs and ionic currents are shown after 300 s stimulation. 1 µM isoproterenol is applied at time *t* = 0 s.

We used the model to investigate the effects of the L-type Ca^2+^ channel block in different cellular compartments on the action potential and [Ca^2+^]_i_ transients in control and after application of 1 µM isoproterenol (Fig. 23). The model cells are electrically stimulated at the frequency 1 Hz. In control, we observed a little change in action potential shape when the caveolae-linked I_CaL_ (I_CaL,cav_) is blocked (APD_50_ decreased by only 2%), whereas a much larger change in the AP is observed during block of I_CaL_ in the extracaveolae compartment (I_CaL,ecav_) (APD_50_ decreased by 14%) (Fig. 23, A). The magnitude of the total I_CaL_ changed by ∼20% during the block of I_CaL,cav_, while the I_CaL_ is almost abolished by the block of I_CaL,ecav_ (Fig. 23, B). The I_CaL,cav_ block relatively slightly (by 25%) decreases [Ca^2+^]_i_ transients, similar to the experimental finding by [70], while at the I_CaL,ecav_ block such transient is not present (Fig. 23, C).

When the model cell is stimulated by isoproterenol, the specific block of I_CaL,cav_ only slightly decreased the total cellular I_CaL_ (Fig. 23, E), resulting in more pronounced shortening of APD_50_ (by 8%, Fig. 23, D) compared to control. Even larger AP shortening is obtained with the specific block of I_CaL,ecav_ (by 28%). More significant effect of I_CaL,cav_ block on [Ca^2+^]_i_ transients is predicted by simulations with isoproterenol than for control conditions (a decrease by 28%), and [Ca^2+^]_i_ transients is almost abolished with the I_CaL,ecav_ block (Fig. 23, F). Further experiments on the effects of different populations of the L-type Ca^2+^ channels on the action potential and [Ca^2+^]_i_ transients are necessary to verify model predictions.

## Discussion

In this paper, a new compartmentalized model for the β_1_-adrenergic signaling system in mouse ventricular myocytes is developed. This model is based on our previously published model for an apical cardiac cell [25], [26], which includes a comprehensive description of action potential, ionic currents, and Ca^2+^ dynamics. The new model includes biochemical and electrophysiological parts of the β_1_-adrenergic signaling pathway, which are extensively verified by the existing experimental data. The model successfully reproduces cAMP dynamics, activation of adenylyl cyclases and phosphodiesterases, protein kinase A and its targets, voltage-clamp protocols for major repolarization currents, action potential modification, and changes in the Ca^2+^ handling mechanism upon stimulation of the β_1_-adrenergic signaling system. The model allows for elucidation of the mechanisms of action potential prolongation and increase in [Ca^2+^]_i_ transients. Simulations also show the absence of the negative staircase effect in [Ca^2+^]_i_ transients upon stimulation of β_1_-adrenoceptors, which was found experimentally. The model also allows for some testable predictions, such as frequency dependences of action potential durations and [Ca^2+^]_i_ transient amplitudes. The model highlights the importance of compartmentalization of the β_1_-adrenergic signaling system in mouse ventricular myocytes by the description of the two populations of the L-type Ca^2+^ channels, in caveolae and extracaveolae compartments, and their distinct functional role in myocyte function, as well as the differential localization and function of other PKA targets.

### Compartmentalization of the β_1_-adrenergic Signaling System

The β-adrenergic signaling system plays a significant role in the function of the heart. There are two types of β-adrenergic receptors in cardiomyocytes (β_1_- and β_2_-adrenergic receptors), which are different in their cellular localization and function. Experimental data from rat ventricular myocytes by Rybin et al. [20] shows that β_1_-adrenergic receptors are distributed between caveolae, non-caveolae membrane fractions, and internal membranes, with the majority of the β_1_-adrenergic receptors being found outside of the caveolae compartment. In contrast, β_2_-adrenergic receptors (β_2_-ARs) are localized in the caveolae compartment [20]. Similar data were obtained for mouse ventricular myocytes [19]. According to the experimental finding, our model includes only 1% of β_1_-adrenergic receptors in the caveolae compartment, with the majority of them distributed between the extracaveolae and cytosolic compartments. Such localization suggests different functional consequences: selective activation of β_1_-adrenergic receptors with isoproterenol in mouse ventricular myocytes leads to a significant increase in [Ca^2+^]_i_ transient and myocyte contraction, while selective activation of β_2_-adrenergic receptors with zinterol does not have any effect on myocyte contractility [134]. Activation of β_1_-adrenergic receptors also results in phosphorylation of major cytosolic proteins, such as phospholamban and troponin I, while stimulation of β_2_-adrenergic receptors does not produce this effect [135].

In addition to differential localization of β_1_- and β_2_-adrenergic receptors, experimental data also demonstrates differential localization of signaling proteins involved in the β_1_-adrenergic signaling pathway, suggesting that even activation of one signaling system is a complex process, which proceeds differently in separate compartments. It has been found that adenylyl cyclases of type V/VI are predominantly localized in caveolae, while types IV and VII are located in non-caveolae membrane fractions and cytosol [28], [35], [36]. There is also differential distribution of three major types of phosphodiesterases in subcellular compartments (PDE2, PDE3, and PDE4 [48]), which also causes different effects on cAMP degradation [123]. Our model successfully reproduces the activities of ACs and PDEs in different compartments, and the differential effects of PDE3 and PDE4 inhibition in production of cAMP transients. For example, steady-state cAMP concentration is smaller and cAMP transient is less prolonged in the caveolae compartment than in the extracaveolae and cytosol (Fig. 15), suggesting larger activity of PDEs in the caveolae.

Finally, the major protein kinase A phosphorylation targets are also localized in different subcellular compartments. Two of them are found predominantly in the caveolae compartment (the fast Na^+^ current and the phospholemman, which regulates the Na^+^-K^+^ pump), five others (I_CaL_, I_Kto,f_, I_Kur_, I_K1_, and ryanodine receptors) are predominantly expressed in non-caveolae membrane domains, and phospholamban and troponin I are located in the cytosol (see Methods for details). It is remarkable that one of the PKA targets, the L-type Ca^2+^ channel, is expressed in both the caveolae and extracaveolae compartments, with different a physiological role in each domain. In addition to the different distribution of two major protein kinase A isoforms (PKAI and PKAII), there is also a different localization of the major dephosphorylation proteins (protein phosphatase 1 and protein phosphatase 2A). Both PP1 and PP2A are found in the caveolae and cytosolic compartments, however, only PP1 plays an important functional role in the extracaveolae. Such distribution of the targets results in their different phosphorylation kinetics and magnitude, producing a very complex interaction relationship in the effects on action potential and [Ca^2+^]_i_ transients.

Our model of the β_1_-adrenergic signaling system includes differential subcellular localization of both signaling and target proteins, and is able to reproduce both phosphorylation kinetics and concentration-dependence of protein phosphorylation by isoproterenol (see Methods). Several non-compartmentalized models were developed for β_1_-adrenergic signaling system in different species, including mice [9], [10], [11], [12]. The models were extensively verified by the available experimental data on the effects of activation of β_1_-adrenergic receptors on electrical activity and ionic homeostasis. However, as the experimental technique is improving and the new data on subcellular organization of signaling systems is accumulating, more comprehensive models are required for more precise description of the cellular functions. Such important findings include differential localization of the isoforms of the major signaling proteins in the β_1_-adrenergic signaling system: adenylyl cyclases, phosphodiesterases, and protein kinase A [10], [36], [46]. Discovery of the differential localization of the targets of PKA, such as the ionic currents and contractile proteins, requires more demands on the models of cardiac cells to include multiple subcellular compartments. One of the recent experimental findings of the two populations of the L-type Ca^2+^ channels, the major players in cardiac excitation-contraction coupling, and their differential physiological role in cellular function highlights the needs in compartmentalized models of cardiac myocytes [21], [22]. One such model was developed recently for canine ventricular myocytes and includes three signaling systems, β_1_- and β_2_-adrenergic, and CaMKII-mediated signaling systems [8]. The model described well extensive experimental data and the effects of different signaling systems on the cardiac action potential and [Ca^2+^]_i_ transients. Our model of the β_1_-adrenergic signaling system consists of some elements of the model of Heijman et al. [8], but with significant differences, as outlined above. In particular, the presented model includes different localization of ryanodine receptors and describes two populations of the L-type Ca^2+^ channels based on the most recent experimental data [21], [22]. While the major role of compartmentalization of the components in the β_1_-adrenergic signaling system is clear – separation of the effects of different signaling proteins – the particular role of the signaling molecules in the mechanisms of regulation of the target proteins still needs to be determined.

### The Effects of the β_1_-adrenergic Signaling System on Action Potential

Experimental data shows that the activation of the β_1_-adrenergic signaling system moderately prolongs action potentials duration in mouse ventricular myocytes (Table 1, [125], [126]). Our previous simulation data using the models of mouse ventricular myocytes and ventricular tissues show that the prolongation of action potential can be a pro-arrhythmic factor causing a loss of stability at the cellular level [136] or action potential block at the tissue level at fast pacing rates [137]. Our model reproduces action potential prolongation and elucidates its mechanism. We found that the major effect on action potential at 25% and 50% repolarization comes from an increase in the inward L-type Ca^2+^ current, I_CaL_, and the fast Na^+^ current, I_Na_, and a decrease in the rapidly recovering transient outward K^+^ current, I_Kto,f_, which tend to prolong action potential, and an increase in the ultra-rapidly activating current, I_Kur_, whose role is in action potential shortening (Fig. 17). At 75% and 90% repolarization, more currents, such as the Na^+^/Ca^2+^ exchanger current, the Na^+^-K^+^ pump current, the time-independent K^+^ current, I_K1_, and the Na^+^ background current, I_Nab_, are brought into play (Fig. 17). The total result of interaction is the moderate prolongation of action potential duration. Note that similar prolongation of the action potential duration upon stimulation of the β_1_-adrenergic signaling system was found in rat ventricular cells [10], however, the mechanism of the action potential prolongation and the role of major contributing currents were not investigated.

Frequency dependences of the action potential duration at different levels of repolarization show their different prolongations upon stimulation with isoproterenol (Table 1). While the prolongations of APD_50_ increase with the stimulation frequency, the prolongations of APD_75_ and APD_90_ demonstrate more complex behavior. Such differences are explained by the different major contributing currents to AP duration at different levels of repolarization without and with isoproterenol. A decrease in the prolongation magnitude of APD_90_ compared to APD_50_ upon application of isoproterenol is also observed in some experiments [125], [126]; however, data of Wang et al. [75] demonstrated similar prolongations both for APD_90_ and APD_50_. Further experiments are necessary to investigate this phenomenon.

This effect of β_1_-adrenergic receptor stimulation on action potential is different from that in larger species [8], [11]. Experimental data shows that stimulation of β_1_-ARs reduces action potential duration in rabbits and dogs wild type ventricular myocytes [11], [138]. The data of Volders et al. [138] shows that one of the major contributing factors in action potential shortening is an increase in the slow delayed-rectifier current, I_Ks_, upon activation of β_1_-ARs. The increase in I_Ks_ overcomes the tendency of action potential to prolongation due to an increase in the L-type Ca^2+^ current, I_CaL_ [138]. This result was also confirmed by simulations using models for rabbit and canine action potential which include the β_1_-adrenergic signaling system [8], [11].

Thus, our model for the β_1_-adrenergic signaling system in mouse ventricular myocytes and the models of others for canine and rabbit cardiac cells [8], [11] demonstrate different effects of β_1_-adrenoceptor stimulations on the action potential durations, elucidate the mechanisms of these effects, and reveal the different ionic currents which are responsible for the changes.

### The Effects of the β_1_-adrenergic Signaling System on Ca^2+^ Dynamics

As found experimentally, activation of the β_1_-adrenergic signaling system significantly increases the magnitude of intracellular [Ca^2+^]_i_ transients, depending on the concentration of β_1_-ARs agonist [75], [127], [128], [129], [139]. The effect is more pronounced in rodent ventricular cells where the increase can reach up to 5 times [127], and a smaller effect is observed in larger species, such as rabbit and dog [139], [140]. In addition to an increase in [Ca^2+^]_i_ transient amplitude, β_1_-AR agonists increase the rate of [Ca^2+^]_i_ decline.

There are several points of view on what is the major cause of the inotropy and lusitropy in the heart. It is clear that phosphorylation of phospholamban increases the pumping of Ca^2+^ into the SR upon stimulation of β_1_-AR and is considered a crucial regulator of cardiac function [27], [141]. However, this phospholamban phosphorylation is not the only reason for such increase. Recently Eisner et al. [142] analyzed the major contributing factor to positive cardiac inotropy upon stimulation of β_1_-ARs. They considered four proteins that are affected by adrenergic stimulation: ryanodine receptors, SERCA pump, L-type Ca^2+^ channels, and troponin. Their analysis has shown that the L-type Ca^2+^ current is a major player that leads to positive cardiac inotropy. In our model, stimulation of the β_1_-adrenergic signaling system increases I_CaL_ by about twice compared to control cells (Fig. 9 [79], [80], [81]). This increase approximately doubles Ca^2+^ influx into the myocyte (see also our simulation data in Fig. 19), while Ca^2+^ extrusion from the myocyte, predominantly by the Na^+^/Ca^2+^ exchanger, does not increase to the same degree. The resulting effect is an increase in Ca^2+^ influx into cell until a new dynamic quasi-steady-state is reached. Thus, our modeling data supports the view that the L-type Ca^2+^ current is a major player in cardiac inotropy in mouse ventricular myocytes, as also suggested by Eisner et al. [142].

Our model also supports the idea that the key contributor to cardiac lusitropy upon stimulation of β_1_-ARs is the SERCA Ca^2+^ pump. In mouse and rat ventricular myocytes, about 90% of the released Ca^2+^ is pumped back to the sarcoplasmic reticulum compared to about 70% in rabbits and larger species [27]. An estimation of Ca^2+^ influx into the SR by the SERCA pump during one cardiac cycle (1 Hz stimulation) before and after activation of the β_1_-adrenergic signaling system is 36 µM and 61 µM, respectively. This estimation correlates with about a two-fold decrease in the time constant of [Ca^2+^]_i_ relaxation (Fig. 18). While the Na^+^/Ca^2+^ exchanger also contributes to the [Ca^2+^]_i_ relaxation, its contribution in mouse ventricular myocytes is less than 10%. In larger species, the Na^+^/Ca^2+^ exchanger can make a larger contribution to the lusitropic effect, as its share is about 25–30% of the total released Ca^2+^.

There is also a long-term dispute among two groups of scientists who study ryanodine receptors (Marks group on the one hand and Valdivia and Houser group on the other) related to the physiological role of RyR phosphorylation in cardiac function [143]. Experimental data of Marks and co-authors demonstrated that enhanced phosphorylation of RyRs at S2808 in failing hearts results in an increased Ca^2+^ leak from the SR and leads to an increased arrhythmias [143], [144]. The Houser and Valdivia group have shown opposite results: they found that RyR phosphorylation site S2808 does not produce significant physiological effects neither in wild type nor in infarcted mouse hearts [145]. Our simulations shows that phosphorylation of RyRs does not change Ca^2+^ sensitivity and does not produce significant physiological effect during stimulation of the β_1_-adrenergic signaling system, the results which are in line with the data from Houser and Valdivia group.

### The Effects of the β_1_-adrenergic Signaling System on Na^+^ Fluxes

Our model of the β_1_-adrenergic signaling system in mouse ventricular myocytes is able to reproduce experimental data on a decrease in [Na^+^]_i_ concentration upon activation of β_1_-ARs (Fig. 20, [100], [127]). We also estimated a contribution of the different mechanisms of the Na^+^ entry and extrusion from the cell, experimental data for which are not available yet. Simulations at physiological frequency 5 Hz and without application of isoproterenol suggest that about 20% of Na^+^ enter the cell through the fast Na^+^ current, 32% through the Na^+^/Ca^2+^ exchanger, and 48% through the background mechanisms (large Na^+^ gradient, the Na^+^/H^+^ exchanger, the Na^+^/HCO^−^_3_ co-transporter, etc.). With application of isoproterenol, the fraction of Na^+^ entry decreases for the fast Na^+^ current to 17%, increases to 46% for the Na^+^/Ca^2+^ exchanger, and decreases to 37% for the background mechanism. These fractions are different from those estimated for the larger species, such as rabbits [133]. For rabbits, about 22% of Na^+^ enter the cell through the fast Na^+^ current (which is similar to our estimations for mice), 60% through the Na^+^/Ca^2+^ exchanger, and 18% through the background mechanisms, suggesting the Na^+^/Ca^2+^ exchanger to be the major player in the Na^+^ transport into the cell. Further experiments are necessary to verify Na^+^ fluxes structure in mouse ventricular cells without and with application of isoproterenol estimated from the presented model.

### Model Limitations

Despite that the presented model was extensively verified by the experimental data and reproduced most effects which result from stimulation of the β_1_-adrenergic signaling system, it has some limitations. One of these limitations is that some experimental data used for the model verification were obtained from different species, such as rats, canine, and rabbits. In this respect, some of the model parameters can be adjusted to fit the data obtained from mouse ventricular myocytes, when such data becomes available. A second source of the errors in model parameters is the low accuracy of biochemical experiments, which can vary by an order of magnitude (see for example data on PKA activation by cAMP). However, investigations by Soltis and Saucerman [146] are very encouraging about this issue. They have shown that the β_1_-adrenergic signaling system is robust, at least in steady-state, even when the model parameters change by 1–2 orders of magnitude. Third, not all model parameters are measured directly in the experiments, and, therefore, are adjusted numerically to fit the experimental data downstream in the signaling pathway. Finally, a fourth limitation comes from the availability of at least two other, β_2_-adrenergic and CaMKII-mediated, signaling systems that can potentially interact with the β_1_-adrenergic system. Experimental data with mice shows that the β_2_-adrenergic signaling system does not affect the β_1_-adrenergic system in control and upon application of isoproterenol [7]. This phenomenon is explained by the significantly larger concentration of the inhibitory G protein, G_i_, compared to G_s_ in mouse ventricular cells, which inhibits the stimulatory effects of the β_2_-ARs. The effects of β_2_-ARs can only be revealed upon additional application of pertussis toxin which suppresses the activity of G_i_ [7]. While the effects of the CaMKII-mediated signaling system are not taken into account in the presented model, the model of the β_1_-adrenergic signaling system still describes reasonably well most of the available experimental data on mouse ventricular myocytes. The author considers this model as the first step in the development of a more comprehensive compartmentalized model of the adrenergic signaling system in mouse ventricular myocytes.

## Supporting Information

Appendix S1 **Model equations and parameters.**
(PDF)

## References

[1] Rudy Y, Ackerman MJ, Bers DM, Clancy CE, Houser SR, et al (2008) System approach to understanding electromechanical activity in the human heart: a National Heart, Lung, and Blood Institute workshop summary. Circulation 118: 1202–1211. 18779456 10.1161/CIRCULATIONAHA.108.772715 PMC2908516

[2] Schaub MC, Hefti MA, Zaugg M (2006) Integration of calcium with the signaling network in cardiac myocytes. J Mol Cell Cardiol 41: 183–214. 16765984 10.1016/j.yjmcc.2006.04.005

[3] Wachter SB, Gilbert EM (2012) Beta-adrenergic receptors, from their discovery and characterization through their manipulation to beneficial clinical application. Cardiology 122: 104–112. 22759389 10.1159/000339271

[4] Ho D, Yan L, Iwatsubo K, Vatner DE, Vatner SF (2010) Modulation of β-adrenergic receptor signaling in heart failure and longevity: targeting adenylyl cyclase type 5. Heart Fail Rev 15: 495–512. 20658186 10.1007/s10741-010-9183-5 PMC3655553

[5] Reincober J, Tscheschner H, Pleger ST, Most P, Katus HA, et al (2012) Targeting GRK2 by gene therapy for heart failure: benefits above β-blockade. Gene Ther 19: 686–693. 22336718 10.1038/gt.2012.9

[6] Shah AM, Mann DL (2011) In search of new therapeutic targets and strategies for heart failure: recent advances in basic science. Lancet 378: 704–712. 21856484 10.1016/S0140-6736(11)60894-5 PMC3486638

[7] Xiao RP, Zhu W, Zheng M, Chakir K, Bond R, et al (2004) Subtype-specific β-adrenoceptor signaling pathways in the heart and their potential clinical implications. Trends Pharmacol Sci 25: 358–365. 15219978 10.1016/j.tips.2004.05.007

[8] Heijman J, Volders PGA, Westra RL, Rudy Y (2011) Local control of β-adrenergic stimulation: effects on ventricular myocyte electrophysiology and Ca^2+^ transient. J Mol Cell Cardiol 50: 863–871. 21345340 10.1016/j.yjmcc.2011.02.007 PMC3075371

[9] Kuzumoto M, Takeuchi A, Nakai H, Oka C, Noma A, et al (2008) Simulation analysis of intracellular Na^+^ and Cl^−^ homeostasis during β1-adrenergic stimulation of cardiac myocytes. Prog Biophys Mol Biol 96: 171–186. 17826821 10.1016/j.pbiomolbio.2007.07.005

[10] Saucerman JJ, Brunton LL, Michailova AP, McCulloch AD (2003) Modeling β-adrenergic control of cardiac myocyte contractility *in silico* . J Biol Chem 278: 47997–48003. 12972422 10.1074/jbc.M308362200

[11] Saucerman JJ, Healy SN, Belik ME, Puglisi JL, McCulloch AD (2004) Proarrhythmic consequences of a KCNQ1 AKAP-binding domain mutation: computational models of whole cells and heterogeneous tissue. Circ Res 95: 1216–1224. 15528464 10.1161/01.RES.0000150055.06226.4e

[12] Yang JH, Saucerman JJ (2012) Phospholemman is a negative feed-forward regulator of Ca^2+^ in β-adrenergic signaling, accelerating β-adrenergic inotropy. J Mol Cell Cardiol 52: 1048–1055. 22289214 10.1016/j.yjmcc.2011.12.015 PMC3327824

[13] Iancu RV, Jones SW, Harvey RD (2007) Compartmentation of cAMP signaling in cardiac myocytes: a computational study. Biophys J 92: 3317–3331. 17293406 10.1529/biophysj.106.095356 PMC1852367

[14] Iancu RV, Ramamurthy G, Warrier S, Nikolaev VO, Lohse MJ, et al (2008) Cytoplasmic cAMP concentrations in intact cardiac myocytes. Am J Physiol Cell Physiol 295: C414–C422. 18550706 10.1152/ajpcell.00038.2008 PMC2518413

[15] Maguy A, Hebert TE, Nattel S (2006) Involvement of lipid rafts and caveolae in cardiac ion channel function. Cardiovasc Res 69: 798–807. 16405931 10.1016/j.cardiores.2005.11.013

[16] Balijepalli RC, Kamp TJ (2008) Caveolae, ion channels and cardiac arrhythmias. *Prog* Biophys Mol Biol 98: 149–160. 19351512 10.1016/j.pbiomolbio.2009.01.012 PMC2836876

[17] Harvey RD, Calaghan SC (2012) Caveolae create local signalling domains through their distinct protein content, lipid profile and morphology. J Mol Cell Cardiol 52: 366–375. 21782827 10.1016/j.yjmcc.2011.07.007 PMC4120829

[18] Weiss S, Oz S, Benmocha A, Dascal N (2013) Regulation of cardiac L-type Ca^2+^ channel Ca_V_1.2 via the β-adrenergic-cAMP-protein kinase A pathway. Old dogmas, advances, and new uncertainties. Circ Res 113: 617–631. 23948586 10.1161/CIRCRESAHA.113.301781

[19] Balijepalli RC, Foell JD, Hall DD, Hell JW, Kamp TJ (2006) Localization of cardiac L-type Ca^2+^ channels to a caveolar macromolecular signaling complex is required for β_2_-adrenergic regulation. Proc Natl Acad Sci USA 103: 7500–7505. 16648270 10.1073/pnas.0503465103 PMC1564282

[20] Rybin VO, Xu X, Lisanti MP, Steinberg SF (2000) Differential targeting of β-adrenergic receptor subtypes and adenylyl cyclase to cardiomyocyte caveolae: a mechanism to functionally regulate the cAMP signaling pathway. J Biol Chem 275: 41447–41457. 11006286 10.1074/jbc.M006951200

[21] Scriven DRL, Asghari P, Schulson MN, Moore EDW (2010) Analysis of Ca_V_1.2 and ryanodine receptor clusters in rat ventricular myocytes. Biophys J 99: 3923–3929. 21156134 10.1016/j.bpj.2010.11.008 PMC3000512

[22] Best JM, Kamp TJ (2012) Different subcellular populations of L-type Ca^2+^ channels exhibit unique regulation and functional roles in cardiomyocytes. J Mol Cell Cardiol 52: 376–387. 21888911 10.1016/j.yjmcc.2011.08.014 PMC3264751

[23] Engelhardt S, Hein L, Wiesmann F, Lohse MJ (1999) Progressive hypertrophy and heart failure in β_1_-adrenergic receptor transgenic mice. Proc Natl Acad Sci USA 96: 7059–7064. 10359838 10.1073/pnas.96.12.7059 PMC22055

[24] Milano CA, Allen LF, Rockman HA, Dolber PC, McMinn TR, et al (1994) Enhanced myocardial function in transgenic mice overexpressing the β_2_-adrenergic receptor. Science 264: 582–586. 8160017 10.1126/science.8160017

[25] Bondarenko VE, Szigeti GP, Bett GCL, Kim SJ, Rasmusson RL (2004) Computer model of action potential; of mouse ventricular myocytes. Am J Physiol Heart Circ Physiol 287: H1378–H1403. 15142845 10.1152/ajpheart.00185.2003

[26] Petkova-Kirova PS, London B, Salama G, Rasmusson RL, Bondarenko VE (2012) Mathematical modeling mechanisms of arrhythmias in transgenic mouse heart overexpressing TNF-α. Am J Physiol Heart Circ Physiol 302: H934–H952. 22081697 10.1152/ajpheart.00493.2011 PMC3360583

[27] Bers DM (2001) Excitation-Contraction Coupling and Cardiac Contractile Force (2^nd^ ed.). Dordrecht, The Netherlands: Kluwer Academic. 427 p.

[28] Steinberg SF (2004) β_2_-adrenergic receptor signaling complexes in cardiomyocyte caveolae/lipid rafts. J Mol Cell Cardiol 37: 407–415. 15276011 10.1016/j.yjmcc.2004.04.018

[29] Hilal-Dandan R, Kanter JR, Brunton LL (2000) Characterization of G-protein signaling in ventricular myocytes from the adult mouse heart: differences from the rat. J Mol Cell Cardiol 32: 1211–1221. 10860764 10.1006/jmcc.2000.1156

[30] Nikolaev VO, Moshkov A, Lyon AR, Miragoli M, Noval P, et al (2010) β_2_-adrenergic receptor redistribution in heart failure changes cAMP compartmentation. Science 327: 1653–1657. 20185685 10.1126/science.1185988

[31] Lohse MJ, Hein P, Hoffmann C, Nikolaev VO, Vilardaga JP, et al (2008) Kinetics of G-protein-coupled receptor signals in intact cells. Br J Pharmacol 153: S125–S132. 18193071 10.1038/sj.bjp.0707656 PMC2268076

[32] Freedman NJ, Liggett SB, Drachman DE, Pei G, Caron MG, et al (1995) Phosphorylation and desensitization of the human β_1_-adrenergic receptor: involvement of G protein-coupled receptor kinases and cAMP-dependent protein kinase. J Biol Chem 270: 17953–17961. 7629102 10.1074/jbc.270.30.17953

[33] Göttle M, Geduhn J, König B, Gille A, Höcherl K, et al (2009) Characterization of mouse heart adenylyl cyclase. J Pharmacol Exp Ther 329: 1156–1165. 19307450 10.1124/jpet.109.150953

[34] Tang T, Gao MH, Lai NC, Firth AL, Takahashi T, et al (2008) Adenylyl cyclase type 6 deletion decreases left ventricular function via impaired calcium handling. Circulation 117: 61–69. 18071070 10.1161/CIRCULATIONAHA.107.730069

[35] Head BP, Patel HH, Roth DM, Lai NC, Niesman IR, et al (2005) G-protein-coupled receptor signaling components localize in both sarcolemmal and intracellular caveolin-3-associated microdomains in adult cardiac myocytes. J Biol Chem 280: 31036–31044. 15961389 10.1074/jbc.M502540200

[36] Ostrom RS, Insel PA (2004) The evolving role of lipid rafts and caveolae in G protein-coupled receptor signaling: implications for molecular pharmacology. Br J Pharmacol 143: 235–245. 15289291 10.1038/sj.bjp.0705930 PMC1575337

[37] Chen-Goodspeed M, Lukan AN, Dessauer CW (2005) Modeling of Gα_s_ and Gα_i_ regulation of human type V and VI adenylyl cyclase. J Biol Chem 280: 1808–1816. 15545274 10.1074/jbc.M409172200

[38] Gao B, Gilman AG (1991) Cloning and expression of a widely distributed (type IV) adenylyl cyclase. Proc Natl Acad Sci USA 88: 10178–10182. 1946437 10.1073/pnas.88.22.10178 PMC52891

[39] Gao X, Sadana R, Dessauer CW, Patel TB (2007) Conditional stimulation of type V and VI adenylyl cyclases by G protein βγ subunits. J Biol Chem 282: 294–302. 17110384 10.1074/jbc.M607522200

[40] Zimmermann G, Taussig R (1996) Protein kinase C alters the responsiveness of adenylyl cyclases to G protein α and βγ subunits. J Biol Chem 271: 27161–27166. 8900209 10.1074/jbc.271.43.27161

[41] Post SR, Hilal-Dandan R, Urasawa K, Brunton LL, Insel PA (1995) Quantification of signaling components and amplification in the β-adrenergic-receptor-adenylyl cyclase pathway in isolated adult rat ventricular myocytes. Biochem J 311: 75–80. 7575483 10.1042/bj3110075 PMC1136121

[42] Tepe NM, Liggett SB (1999) Transgenic replacement of type V adenylyl cyclase identifies a critical mechanism of β-adrenergic receptor dysfunction in the G_αq_ overexpressing mouse. FEBS Lett 458: 236–240. 10481072 10.1016/s0014-5793(99)01147-3

[43] Lemire I, Allen BG, Rindt H, Hebert TE (1998) Cardiac-specific overexpression of α_1B_AR regulates βAR activity via molecular crosstalk. J Mol Cell Cardiol 30: 1827–1839. 9769238 10.1006/jmcc.1998.0746

[44] Richter W, Xie M, Scheitrum C, Krall J, Movsesian MA, et al (2011) Conserved expression and functions of PDE4 in rodent and human heart. Basic Res Cardiol 106: 249–262. 21161247 10.1007/s00395-010-0138-8 PMC3032896

[45] Bode DC, Kanter JR, Brunton LL (1991) Cellular distribution of phosphodiesterase isoforms in rat cardiac tissue. Circ Res 68: 1070–1079. 1849058 10.1161/01.res.68.4.1070

[46] Fischmeister R, Castro LRV, Abi-Gerges A, Rochais F, Jurevičius J, et al (2006) Compartmentation of cyclic nucleotide signaling in the heart: the role of cyclic nucleotide phosphodiesterases. Circ Res 99: 816–828. 17038651 10.1161/01.RES.0000246118.98832.04

[47] Osadchii OE (2007) Myocardial phosphodiesterases and regulation of cardiac contractility in health and cardiac disease. Cardiovasc Drugs Ther 21: 171–194. 17373584 10.1007/s10557-007-6014-6

[48] Mongillo M, Tocchetti CG, Terrin A, Lissandron V, Cheung YF, et al (2006) Compartmentalized phosphodiesterase-2 activity blunts β-adrenergic cardiac inotropy via an NO/cGMP-dependent pathway. Circ Res 98: 226–234. 16357307 10.1161/01.RES.0000200178.34179.93

[49] Georget M, Mateo P, Vandecasteele G, Lipskaia L, Defer N, et al (2003) Cyclic AMP compartmentation due to increase cAMP-phosphodiesterase activity in transgenic mice with a cardiac-directed expression of the human adenylyl cyclase type 8 (AC8). FASEB J 17: 1380–1391. 12890691 10.1096/fj.02-0784com

[50] Adams SR, Harootunian AT, Buechler YJ, Taylor SS, Tsien RY (1991) Fluorescence ratio imaging of cyclic AMP in single cell. Nature 349: 694–697. 1847505 10.1038/349694a0

[51] Dostmann WRG, Taylor SS (1991) Identifying the molecular switches that determine whether (R_p_)-cAMPS functions as an antagonist or an agonist in the activation of cAMP-dependent protein kinase I. Biochemistry. 30: 8710–8716. 10.1021/bi00099a032 1653606

[52] Lin F, Owens A, Chen S, Stevens ME, Kesteven S, et al (2001) Targeted α_1A_-adrenergic receptor overexpression induces enhanced cardiac contractility but not hypertrophy. Circ Res 89: 343–350. 11509451 10.1161/hh1601.095912

[53] Roman BB, Goldspink PH, Spaite E, Urboniene D, McKinney R, et al (2004) Inhibition of PKC phosphorylation of TnI improves cardiac performance in vivo. Am J Physiol Heart Circ Physiol 286: H2089–H2095. 14726296 10.1152/ajpheart.00582.2003

[54] Beavo JA, Bechtel PJ, Krebs EG (1974) Activation of protein kinase by physiological concentrations of cyclic AMP. Proc Natl Acad Sci USA 71: 3580–3583. 4372627 10.1073/pnas.71.9.3580 PMC433818

[55] Dao KK, Teigen K, Kopperud R, Hodneland E, Schwede F, et al (2006) Epac1 and cAMP-dependent protein kinase holoenzyme have similar cAMP affinity, but their cAMP domains have distinct structural features and cyclic nucleotide recognition. J Biol Chem 281: 21500–21511. 16728394 10.1074/jbc.M603116200

[56] Hofmann F, Beavo JA, Bechtel PJ, Krebs EG (1975) Comparison of adenosine 3′: 5′-monophosphate-dependent protein kinase from rabbit skeletal and bovine heart muscle. J Biol Chem 250: 7795–7801. 170270

[57] Buxton ILO, Brunton LL (1983) Compartments of cyclic AMP and protein kinase in mammalian cardiomyocytes. J Biol Chem 258: 10233–10239. 6309796

[58] Hescheler J, Kameyama M, Trautwein W, Mieskes G, Söling HD (1987) Regulation of the cardiac calcium channel by protein phosphatases. Eur J Biochem 165: 261–266. 2439329 10.1111/j.1432-1033.1987.tb11436.x

[59] MacDougall LK, Jones LR, Cohen P (1991) Identification of the major protein phosphatases in mammalian cardiac muscle which dephosphorylate phospholamban. Eur J Biochem 196: 725–734. 1849481 10.1111/j.1432-1033.1991.tb15871.x

[60] Neumann J, Boknik P, Herzig S, Schmitz W, Scholz H, et al (1993) Evidence for physiological functions of protein phosphatases in the heart: evaluation with okadaic acid. Am J Physiol Heart Circ Physiol 265: H257–H266. 10.1152/ajpheart.1993.265.1.H257 8393625

[61] duBell WH, Gigena MS, Guatimosim S, Long X, Lederer WJ, et al (2002) Effects of PP1/PP2A inhibitor calyculin A on the E-C coupling cascade in murine ventricular myocytes. Am J Physiol Heart Circ Physiol 282: H38–H48. 11748045 10.1152/ajpheart.00536.2001

[62] Müller FU, Lewin G, Baba HA, Boknik P, Fabritz L, et al (2005) Heart-directed expression of a human cardiac isoform of cAMP-response element modulator in transgenic mice. J Biol Chem 280: 6906–6914. 15569686 10.1074/jbc.M407864200

[63] Herzig S, Neumann J (2000) Effects of serine/threonine protein phosphatases on ion channels in excitable membranes. Physiol Rev 80: 173–210. 10617768 10.1152/physrev.2000.80.1.173

[64] Carr AN, Schmidt AG, Suzuki Y, del Monte F, Sato Y, et al (2002) Type 1 phosphatase, a negative regulator of cardiac function. Mol Cell Biol 22: 4124–4135. 12024026 10.1128/MCB.22.12.4124-4135.2002 PMC133876

[65] El-Armouche A, Wittköpper K, Degenhardt F, Weinberger F, Didié M, et al (2008) Phosphatase inhibitor-1-deficient mice are protected from catecholamine-induced arrhythmias and myocardial hypertrophy. Cardiovasc Res 80: 396–406. 18689792 10.1093/cvr/cvn208

[66] Nicolaou P, Hajjar RJ, Kranias EG (2009) Role of protein phosphatase-1 inhibitor-1 in cardiac physiology and pathophysiology. J Mol Cell Cardiol 47: 365–371. 19481088 10.1016/j.yjmcc.2009.05.010 PMC2716438

[67] Neumann J, Gupta RC, Schmitz W, Scholz H, Nairn AC, et al (1991) Evidence for isoproterenol-induced phosphorylation of phosphatase inhibitor-1 in the intact heart. Circ Res 69: 1450–1457. 1659500 10.1161/01.res.69.6.1450

[68] O’Connell TD, Ni YG, Lin KM, Han H, Yan Z (2003) Isolation and culture of adult mouse cardiac myocytes for signaling studies. AfCS Research Reports 1: CM0005.

[69] Shibata EF, Brown TLY, Washburn ZW, Bai J, Revak TJ, et al. (2006) Autonomic regulation of voltage-gated cardiac ion channels. J Cardiovasc Electrophysiol (Suppl 1): S34–S42. 10.1111/j.1540-8167.2006.00387.x 16686680

[70] Makarewich CA, Correll RN, Gao H, Zhang H, Yang B, et al (2012) A caveolae-targeted L-type Ca^2+^ channel antagonist inhibits hypertrophic signaling without reducing cardiac contractility. Circ Res 110: 669–674. 22302787 10.1161/CIRCRESAHA.111.264028 PMC3324037

[71] Chu G, Luo W, Slack JP, Tilgmann C, Sweet WE, et al (1996) Compensatory mechanisms associated with the hyperdynamic function of phospholamban-deficient mouse hearts. Circ Res 79: 1064–1076. 8943945 10.1161/01.res.79.6.1064

[72] Bers DM, Stiffel VM (1993) Ratio of ryanodine to dihydropyridine receptors in cardiac and skeletal muscle and implications for E-C coupling. Am J Physiol Cell Physiol 264: C1587–C1593. 10.1152/ajpcell.1993.264.6.C1587 8333507

[73] Bracken N, ElKadri M, Hart G, Hussain M (2006) The role of constitutive PKA-mediated phosphorylation in the regulation of basal *I* _Ca_ in isolated rat cardiac myocytes. Br J Pharmacol 148: 1108–1115. 16799650 10.1038/sj.bjp.0706809 PMC1752019

[74] Tang M, Zhang X, Li Y, Guan Y, Ai X, et al (2010) Enhanced basal contractility but reduced excitation-contraction coupling efficiency and β-adrenergic reserve of hearts with increased Cav1.2 activity. Am J Physiol Heart Circ Physiol 299: H519–H528. 20543081 10.1152/ajpheart.00265.2010 PMC2930392

[75] Wang H, Kohr MJ, Wheeler DG, Ziolo MT (2008) Endothelial nitric oxide synthase decreases β-adrenergic responsiveness via inhibition of the L-type Ca^2+^ current. Am J Physiol Heart Circ Physiol 294: H1473–H1480. 18203845 10.1152/ajpheart.01249.2007 PMC2744450

[76] Tsien RW, Bean BP, Hess P, Lansman JB, Nilius B, et al (1986) Mechanisms of calcium channel modulation by β-adrenergic agents and dihydropyridine calcium agonists. J Mol Cell Cardiol 18: 691–710. 2427730 10.1016/s0022-2828(86)80941-5

[77] Hulme JT, Westenbroek RE, Scheuer T, Catterall WA (2006) Phosphorylation of serine 1928 in the distal C-terminal domain of cardiac Ca_v_1.2 channels during β1-adrenergic regulation. Proc Natl Acad Sci USA 103: 16574–16579. 17053072 10.1073/pnas.0607294103 PMC1637623

[78] Kameyama M, Hescheler J, Hofmann F, Trautwein W (1986) Modulation of Ca current during the phosphorylation cycle in the guinea pig heart. Pflügers Arch 407: 123–128. 2428003 10.1007/BF00580662

[79] Kim SJ, Yatani A, Vatner DE, Yamamoto S, Ishikawa Y, et al (1999) Differential regulation of inotropy and lusitropy in overexpressed Gsα myocytes through cAMP and Ca^2+^ channel pathways. J Clin Invest 103: 1089–1097. 10194482 10.1172/JCI4848 PMC408254

[80] Sako H, Green SA, Kranias EG, Yatani A (1997) Modulation of cardiac Ca^2+^ channels by isoproterenol studied in transgenic mice with altered SR Ca^2+^ content. Am J Physiol Cell Physiol 273: C1666–C1672. 10.1152/ajpcell.1997.273.5.C1666 9374653

[81] Mitarai S, Reed TD, Yatani A (2000) Changes in ionic currents and β-adrenergic receptor signaling in hypertrophied myocytes overexpressing Gα_q_ . Am J Physiol Heart Circ Physiol 279: H139–H148. 10899051 10.1152/ajpheart.2000.279.1.H139

[82] Yarbrough TL, Lu T, Lee HC, Shibata EF (2002) Localization of cardiac sodium channels in caveolin-rich membrane domains. Regulation of sodium current amplitude. Circ Res 90: 443–449. 11884374 10.1161/hh0402.105177

[83] Palygin OA, Pettus JM, Shibata EF (2008) Regulation of caveolar cardiac sodium current by a single G_s_α histidine residue. Am J Physiol Heart Circ Physiol 294: H1693–H1699. 18281377 10.1152/ajpheart.01337.2007

[84] Kirstein M, Eickhorn R, Kochsiek K, Langenfeld H (1996) Dose-dependent alteration of rat cardiac sodium current by isoproterenol: results from direct measurements on multicellular preparations. Pflügers Arch 431: 395–401. 8584433 10.1007/BF02207277

[85] Matsuda JJ, Lee HC, Shibata EF (1993) Acetylcholine reversal of isoproterenol-stimulated sodium currents in rabbit ventricular myocytes. Circ Res 72: 517–525. 8431982 10.1161/01.res.72.3.517

[86] Baba S, Dun W, Boyden PA (2004) Can PKA activators rescue Na^+^ channel function in epicardial border zone cells that survive in the infarcted canine heart? Cardiovasc Res 64: 260–267. 15485685 10.1016/j.cardiores.2004.06.021

[87] Ono K, Fozzard HA, Hanck DA (1993) Mechanism of cAMP-dependent modulation of cardiac sodium channel current kinetics. Circ Res 72: 807–815. 8383015 10.1161/01.res.72.4.807

[88] Zhou J, Yi J, Hu NN, George AL Jr, Murray KT (2000) Activation of protein kinase A modulates trafficking of the human cardiac sodium channel in *Xenopus* oocytes. Circ Res 87: 33–38. 10884369 10.1161/01.res.87.1.33

[89] Rook MB, Evers MM, Vos MA, Bierhuizen MFA (2012) Biology of cardiac sodium channel Na_v_1.5 expression. Cardiovasc Res 93: 12–23. 21937582 10.1093/cvr/cvr252

[90] Clancy CE, Rudy Y (2002) Na^+^ channel mutation that causes both Brugada and long-QT syndrome phenotypes: a simulation study of mechanism. Circulation 105: 1208–1213. 11889015 10.1161/hc1002.105183 PMC1997279

[91] Jayasinghe ID, Cannell MB, Soeller C (2009) Organization of ryanodine receptors, transverse tubules, and sodium-calcium exchanger in rat myocytes. Biophys J 97: 2664–2673. 19917219 10.1016/j.bpj.2009.08.036 PMC2776253

[92] Wong J, Baddeley D, Bushong EA, Yu Z, Ellisman MH, et al (2013) Nanoscale distribution of ryanodine receptors and caveolin-3 in mouse ventricular myocytes: Dilation of t-tubules near junctions. Biophys J 104: L22–L24. 23746531 10.1016/j.bpj.2013.02.059 PMC3672889

[93] Xiao B, Tian X, Xie W, Jones PP, Cai S, et al (2007) Functional consequence of protein kinase A-dependent phosphorylation of the cardiac ryanodine receptor: sensitization of store overload-induced Ca^2+^ release. J Biol Chem 282: 30256–30264. 17693412 10.1074/jbc.M703510200

[94] Xiao B, Zhong G, Obayashi M, Yang D, Chen K, et al (2006) Ser-2030, but not Ser-2808, is the major phosphorylation site in cardiac ryanodine receptors responding to protein kinase A activation upon β-adrenergic stimulation in normal and failing hearts. Biochem J 396: 7–16. 16483256 10.1042/BJ20060116 PMC1449991

[95] Takasago T, Imagawa T, Furukawa KI, Ogurusu T, Shigekawa M (1991) Regulation of the cardiac ryanodine receptor by protein kinase-dependent phosphorylation. J Biochem 109: 163–170. 1849885 10.1093/oxfordjournals.jbchem.a123339

[96] Xiao J, Tian X, Jones PP, Bolstad J, Kong H, et al (2007) Removal of FKBP12.6 does not alter the conductance and activation of the cardiac ryanodine receptor or the susceptibility to stress-induced ventricular arrhythmias. J Biol Chem 282: 34828–34838. 17921453 10.1074/jbc.M707423200 PMC2760432

[97] Liu L, Askari A (2006) β-Subunit of cardiac Na^+^-K^+^-ATPase dictates the concentration of the functional enzyme in caveolae. Am J Physiol Cell Physiol 291: C569–C578. 16624992 10.1152/ajpcell.00002.2006

[98] Fuller W, Eaton P, Medina RA, Bell J, Shattock MJ (2001) Differential centrifugation separates cardiac sarcolemmal and endosomal membranes from Langendorff-perfused rat hearts. Anal Biochem 293: 216–223. 11399035 10.1006/abio.2001.5127

[99] Fuller W, Tulloch LB, Shattock MJ, Calaghan SC, Howie J, et al (2013) Regulation of the cardiac sodium pump. Cell Mol Life Sci 70: 1357–1380. 22955490 10.1007/s00018-012-1134-y PMC3607738

[100] Despa S, Bossuyt J, Han F, Ginsburg KS, Jia LG, et al (2005) Phospholemman-phosphorylation mediates the β-adrenergic effects on Na/K pump function in cardiac myocytes. Circ Res 97: 252–259. 16002746 10.1161/01.RES.0000176532.97731.e5

[101] Gao J, Mathias RT, Cohen IS, Baldo GJ (1992) Isoprenaline, Ca^2+^ and the Na^+^-K^+^ pump in guinea-pig ventricular myocytes. J Physiol 449: 689–704. 1326051 10.1113/jphysiol.1992.sp019109 PMC1176102

[102] Abi-Char J, Maguy A, Coulombe A, Balse E, Ratajczak P, et al (2007) Membrane cholesterol modulates Kv1.5 potassium channel distribution and function in rat cardiomyocytes. J Physiol 582: 1205–1217. 17525113 10.1113/jphysiol.2007.134809 PMC2075263

[103] Ravens U, Wettwer E (2011) Ultra-rapid delayed rectifier channels: molecular basis and therapeutic implications. Cardiovasc Res 89: 776–785. 21159668 10.1093/cvr/cvq398

[104] Folco EJ, Liu GX, Koren G (2004) Caveolin-3 and SAP97 form a scaffolding protein complex that regulates the voltage-gated potassium channel Kv1.5. Am J Physiol Heart Circ Physiol 287: H681–H690. 15277200 10.1152/ajpheart.00152.2004

[105] Martens JR, Sakamoto N, Sullivan SA, Grobaski TD, Tamkun MM (2001) Isoform-specific localization of voltage-gated K^+^ channels to distinct lipid raft populations: targeting of Kv1.5 to caveolae. J Biol Chem 276: 8409–8414. 11115511 10.1074/jbc.M009948200

[106] Martínez-Mármol R, Villalonga N, Solé L, Vicente R, Tamkun MM, et al (2008) Multiple Kv1.5 targeting to membrane surface microdomains. J Cell Physiol 217: 667–673. 18668522 10.1002/jcp.21538 PMC2577364

[107] Li GR, Feng J, Wang Z, Fermini B, Nattel S (1996) Adrenergic modulation of ultrarapid delayed rectifier K^+^ current in human atrial myocytes. Circ Res 78: 903–915. 8620611 10.1161/01.res.78.5.903

[108] Yue L, Feng J, Wang Z, Nattel S (1999) Adrenergic control of the ultrarapid delayed rectifier current in canine atrial myocytes. J Physiol 516: 385–398. 10087339 10.1111/j.1469-7793.1999.0385v.x PMC2269270

[109] González de la Fuente M, Barana A, Gόmez R, Amorόs I, Dolz-Gaitόn P, et al (2013) Chronic atrial fibrillation up-regulates β1-adrenoceptors affecting repolarizing currents and action potential duration. Cardiovasc Res 97: 379–388. 23060133 10.1093/cvr/cvs313

[110] Hoffman DA, Johnson D (1998) Downregulation of transient K^+^ channels in dendrites of hippocampal CA1 pyramidal neurons by activation of PKA and PKC. J Neurosci 18: 3521–3528. 9570783 10.1523/JNEUROSCI.18-10-03521.1998 PMC6793167

[111] Zhang M, Fei XW, He YL, Yang G, Mei YA (2009) Bradykinin inhibits the transient outward K^+^ current in mouse Schwann cells via the cAMP/PKA pathway. Am J Physiol Cell Physiol 296: C1364–C1372. 19339513 10.1152/ajpcell.00014.2009

[112] Anumonwo JMB, Lopatin AN (2010) Cardiac strong inward rectifier potassium channels. J Mol Cell Cardiol 48: 45–54. 19703462 10.1016/j.yjmcc.2009.08.013 PMC2813336

[113] Vaidyanathan R, Taffet SM, Vikstrom KL, Anumonwo JMB (2010) Regulation of cardiac inward rectifier potassium current (I_K1_) by synapse-associated protein-97. J Biol Chem 285: 28000–28009. 20530486 10.1074/jbc.M110.110858 PMC2934665

[114] Simmerman HKB, Jones LR (1998) Phospholamban: protein structure, mechanism of action, and role in cardiac function. Physiol Rev 78: 921–947. 9790566 10.1152/physrev.1998.78.4.921

[115] Wolska BM (2009) Calcineurin and cardiac function: is more or less better for the heart? Am J Physiol Heart Circ Physiol 297: H1576–H1577. 19749162 10.1152/ajpheart.00833.2009

[116] Karczewski P, Bartel S, Krause EG (1990) Differential sensitivity to isoprenaline of troponin I and phospholamban phosphorylation in isolated rat hearts. Biochem J 266: 115–122. 2155603 10.1042/bj2660115 PMC1131103

[117] Kuschel M, Karczewski P, Hempel P, Schlegel WP, Krause EG, et al (1999) Ser^16^ prevails over Thr^17^ phospholamban phosphorylation in the β-adrenergic regulation of cardiac relaxation. Am J Physiol Heart Circ Physiol 276: H1625–H1633. 10.1152/ajpheart.1999.276.5.H1625 10330247

[118] Li L, Desantiago J, Chu G, Kranias EG, Bers DM (2000) Phosphorylation of phospholamban and troponin I in β-adrenergic-induced acceleration of cardiac relaxation. Am J Physiol Heart Circ Physiol 278: H769–H779. 10710345 10.1152/ajpheart.2000.278.3.H769

[119] Lindemann JP, Jones LR, Hathaway DR, Henry BG, Watanabe AM (1983) β-adrenergic stimulation of phospholamban phosphorylation and Ca^2+^-ATPase activity in guinea pig ventricles. J Biol Chem 258: 464–471. 6217205

[120] Sulakhe PV, Vo XT (1995) Regulation of phospholamban and troponin-I phosphorylation in the intact rat cardiomyocytes by adrenergic and cholinergic stimuli: roles of cyclic nucleotides, calcium, protein kinases and phosphatases and depolarization. Mol Cell Biochem 149/150: 103–126. 10.1007/BF01076569 8569720

[121] Robertson SP, Johnson JD, Holroyde MJ, Kranias EG, Potter JD, et al (1982) The effect of troponin I phosphorylation on the Ca^2+^-binding properties of the Ca^2+^-regulatory site of bovine cardiac troponin. J Biol Chem 257: 260–263. 7053370

[122] Bondarenko VE, Bett GCL, Rasmusson RL (2004) A model of graded calcium release and L-type Ca^2+^ channel inactivation in cardiac muscle. Am J Physiol Heart Circ Physiol 286: H1154–H1169. 14630639 10.1152/ajpheart.00168.2003

[123] Leroy J, Abi-Gerges A, Nikolaev VO, Richter W, Lechêne P, et al (2008) Spatiotemporal dynamics of β-adrenergic cAMP signals and L-type Ca^2+^ channel regulation in adult rat ventricular myocytes: role of phosphodiesterases. Circ Res 102: 1091–1100. 18369156 10.1161/CIRCRESAHA.107.167817

[124] Mongillo M, McSorley T, Evellin S, Sood A, Lissandron V, et al (2004) Fluorescence resonance energy transfer-based analysis of cAMP dynamics in live neonatal rat cardiac myocytes reveals distinct functions of compartmentalized phosphodiesterases. Circ Res 95: 67–75. 15178638 10.1161/01.RES.0000134629.84732.11

[125] Tong XY, Porter LM, Liu GX, Dhar-Chowdhury P, Srivastava S, et al (2006) Consequences of cardiac myocyte-specific ablation of K_ATP_ channels in transgenic mice expressing dominant negative Kir6 subunits. Am J Physiol Heart Circ Physiol 291: H543–H551. 16501027 10.1152/ajpheart.00051.2006 PMC2950019

[126] Wu Y, Temple J, Zhang R, Dzhura I, Zhang W, et al (2002) Calmodulin kinase II and arrhythmias in a mouse model of cardiac hypertrophy. Circulation 106: 1288–1293. 12208807 10.1161/01.cir.0000027583.73268.e7

[127] Despa S, Tucker AL, Bers DM (2008) Phospholemman-mediated activation of Na/K-ATPase limits [Na]_i_ and inotropic state during β-adrenergic stimulation in mouse ventricular myocytes. Circulation 117: 1849–1855. 18362230 10.1161/CIRCULATIONAHA.107.754051 PMC2854005

[128] Knollmann BC, Kirchhof P, Sirenko SG, Degen H, Greene AE, et al (2003) Familial hypertrophic cardiomyopathy-linked mutant troponin T causes stress-induced ventricular tachycardia and Ca^2+^-dependent action potential remodeling. Circ Res 92: 428–436. 12600890 10.1161/01.RES.0000059562.91384.1A

[129] Liu N, Denegri M, Ruan Y, Avelino-Cruz JE, Perissi A, et al (2011) Short communication: Flecainide exerts an antiarrhythmic effect in a mouse model of catecholaminergic polymorphic ventricular tachycardia by increasing the threshold for triggered activity. Circ Res 109: 291–295. 21680895 10.1161/CIRCRESAHA.111.247338

[130] Song J, Gao E, Wang JF, Zhang XQ, Chan TO, et al (2012) Constitutive overexpression of phosphomimetic phospholemman S68E mutant results in arrhythmias, early mortality, and heart failure: potential involvement of Na^+^/Ca^2+^ exchanger. Am J Physiol Heart Circ Physiol 302: H770–H781. 22081699 10.1152/ajpheart.00733.2011 PMC3353790

[131] Benkusky NA, Weber CS, Scherman JA, Farrell EF, Hacker TA, et al (2007) Intact β-adrenergic response and unmodified progression toward heart failure in mice with genetic ablation of a major protein kinase A phosphorylation site in the cardiac ryanodine receptor. Circ Res 101: 819–829. 17717301 10.1161/CIRCRESAHA.107.153007

[132] Fernández-Velasco M, Rueda A, Rizzi N, Benitah JP, Colombi B, et al (2009) Increased Ca^2+^ sensitivity of the ryanodine receptor mutant RyR2^R4496C^ underlies catecholaminergic polymorphic ventricular tachycardia. Circ Res 104: 201–209. 19096022 10.1161/CIRCRESAHA.108.177493 PMC2796688

[133] Despa S, Bers DM (2013) Na^+^ transport in the normal and failing heart – Remember the balance. J Mol Cell Cardiol 61: 2–10. 23608603 10.1016/j.yjmcc.2013.04.011 PMC3720717

[134] Xiao RP, Avdonin P, Zhou YY, Cheng H, Akhter SA, et al (1999) Coupling of β_2_-adrenoceptor to G_i_ proteins and its physiological relevance in murine cardiac myocytes. Circ Res 84: 43–52. 9915773 10.1161/01.res.84.1.43

[135] Bers DM (2002) Cardiac excitation-contraction coupling. Nature 415: 198–205. 11805843 10.1038/415198a

[136] Bondarenko VE, Rasmusson RL (2010) Transmural heterogeneity of repolarization and Ca^2+^ handling in a model of mouse ventricular tissue. Am J Physiol Heart Circ Physiol 299: H454–H469. 20525874 10.1152/ajpheart.00907.2009 PMC2931428

[137] Bondarenko VE, Rasmusson RL (2007) Simulations of propagated mouse ventricular action potentials: effects of molecular heterogeneity. Am J Physiol Heart Circ Physiol 293: H1816–H1832. 17586617 10.1152/ajpheart.00471.2007

[138] Volders PGA, Stengl M, van Opstal JM, Gerlach U, Spätjens RLHMG, et al (2003) Probing the contribution of *I* _Ks_ to canine ventricular repolarization: key role for β-adrenergic receptor stimulation. Circulation 107: 2753–2760. 12756150 10.1161/01.CIR.0000068344.54010.B3

[139] Pogwizd SM, Schlotthauer K, Li L, Yuan W, Bers DM (2001) Arrhythmogenesis and contractile dysfunction in heart failure: roles of sodium-calcium exchange, inward rectifier potassium current, and residual β-adrenergic responsiveness. Circ Res 88: 1159–1167. 11397782 10.1161/hh1101.091193

[140] Yamada KA, Corr PB (1992) Effects of β-adrenergic receptor activation on intracellular calcium and membrane potential in adult cardiac myocytes. J Cardiovasc Electrophysiol 3: 209–224.

[141] MacLennan DH, Kranias EG (2003) Phospholamban: a crucial regulator of cardiac contractility. Nat Rev Mol Cell Biol 4: 566–577. 12838339 10.1038/nrm1151

[142] Eisner D, Bode E, Venetucci L, Trafford A (2013) Calcium flux balance in the heart. J Mol Cell Cardiol 58: 110–117. 23220128 10.1016/j.yjmcc.2012.11.017

[143] Bers DM (2012) Ryanodine receptor S2808 phosphorylation in heart failure. Smoking gun or red herring. Circ Res 110: 796–799. 22427320 10.1161/CIRCRESAHA.112.265579

[144] Marx SO, Reiken S, Hisamatsu Y, Jayaraman T, Burkhoff D, et al (2000) PKA phosphorylation dissociates FKBP12.6 from the calcium release channel (ryanodine receptor): defective regulation in failing hearts. Cell 101: 365–376. 10830164 10.1016/s0092-8674(00)80847-8

[145] Zhang H, Makarewich CA, Kubo H, Wang W, Duran JM, et al (2012) Hyperphosphorylation of the cardiac ryanodine receptor at serine 2808 is not involved in cardiac dysfunction after myocardial infarction. Circ Res 110: 831–840. 22302785 10.1161/CIRCRESAHA.111.255158 PMC3322671

[146] Soltis AR, Saucerman JJ (2011) Robustness portraits of diverse biological networks concerved despite order-of-magnitude parameter uncertainty. Bioinformatics 27: 2888–2894. 21880701 10.1093/bioinformatics/btr496 PMC3187657

[147] Rochais F, Abi-Gerges A, Horner K, Lefebvre F, Cooper DMF, et al (2006) A specific pattern of phosphodiesterases controls the cAMP signals generated by different G_s_-coupled receptors in adult rat ventricular myocytes. Circ Res 98: 1081–1088. 16556871 10.1161/01.RES.0000218493.09370.8e PMC2099453

[148] Hohl CM, Li Q (1991) Compartmentation of cAMP in adult canine ventricular myocytes: relation to single-cell free Ca^2+^ transients. Circ Res 69: 1369–1379. 1682066 10.1161/01.res.69.5.1369

