# Supplementary material for: A Compartmentalized Mathematical Model of the β1-Adrenergic Signaling System in Mouse Ventricular Myocytes
Source: PLoS One. 2014 Feb 21;9(2):e89113. doi: 10.1371/journal.pone.0089113 (PMC3931689; doi:10.1371/journal.pone.0089113)
Supplement: Appendix S1 — Model equations and parameters. (PDF) [file pone.0089113.s001.pdf]

APPENDIX S1. MODEL EQUATIONS AND PARAMETERS.

BIOCHEMICAL PART

Cell compartments

Parameter	Definition	Value	Reference
A_{cap}	Capacitive membrane area	1.534×10^{-4} cm^2	Bondarenko et al. [25]
V^{cell}	Cell volume	38.00×10^{-6} μl	Bondarenko et al. [25]
V^{cyt}	Cytosolic volume	25.84×10^{-6} μl	Bondarenko et al. [25]
V_{JSR}	Junctional SR volume	0.12×10^{-6} μl	Bondarenko et al. [25]
V_{NSR}	Network SR volume	2.098×10^{-6} μl	Bondarenko et al. [25]
V_{ss}	Subspace volume	1.485×10^{-9} μl	Bondarenko et al. [25]
V^{cav}	Caveolar volume	$0.02\times V_{cell}$	Heijman et al. [8]
V^{ecav}	Extracaveolar volume	$0.04\times V_{cell}$	Heijman et al. [8]

The protein P concentrations in the cell ($[P]^{cell}$), caveolae, extracaveolae, and cytosol

$$[P]^{cav} = f_P^{cav}\cdot[P]^{cell}\cdot\frac{V^{cell}}{V^{cav}} \quad (A.1)$$

$$[P]^{ecav} = f_P^{ecav}\cdot[P]^{cell}\cdot\frac{V^{cell}}{V^{ecav}} \quad (A.2)$$

$$[P]^{cyt} = (1-f_P^{cav}-f_P^{ecav})\cdot[P]^{cell}\cdot\frac{V^{cell}}{V^{cyt}} \quad (A.3)$$

β_1-adrenergic receptor module

Parameter	Definition	Value	Reference
$[L]$	Ligand concentration	0…100 μM	
$[R_{\beta1}]_{tot}$	Total β_1-adrenoceptor concentration	0.0103 μM	Hilal-Dandan et al. [29]
$f_{\beta1}^{cav}$	Fraction of β_1-adrenoceptors located in caveolae	0.01	Rybin et al. [20] Balijepali et al. [19]
$f_{\beta1}^{ecav}$	Fraction of β_1-adrenoceptors located in extracaveolae	0.5	Rybin et al. [20] Balijepali et al. [19]
$f_{\beta1}^{cyt}$	Fraction of β_1-adrenoceptors located in cytosol	$f_{\beta1}^{cyt} = 1-f_{\beta1}^{cav}-f_{\beta1}^{ecav}$	Rybin et al. [20] Balijepali et al. [19]
$[G_s]_{tot}$	Total concentration of G_s protein	2.054 μM	Post et al. [41]
f_{Gs}^{cav}	Fraction of G_s protein located in caveolae	0.4	Rybin et al. [20]
f_{Gs}^{ecav}	Fraction of G_s protein located in extracaveolae	0.4	Rybin et al. [20]

f_{Gs}^{cyt}	Fraction of G_s protein located in cytosol	$f_{Gs}^{cyt} = 1 - f_{Gs}^{cav} - f_{Gs}^{ecav}$	
$K_{\beta 1,L}$	Low affinity constant of β_1-adrenoceptor for isoproterenol	0.567 μM	Heijman et al. [8]
$K_{\beta 1,H}$	High affinity constant of β_1-adrenoceptor for isoproterenol	0.0617 μM	Heijman et al. [8]
$K_{\beta 1,C}$	Affinity constant of β_1-adrenoceptor for G_s protein	2.86 μM	This paper
k_{PKA+}	Rate of PKA phosphorylation of β_1-adrenoceptor	0.00081 μM^{-1} s^{-1}	Freedman et al. [32]
k_{PKA-}	Rate of PKA dephosphorylation of β_1-adrenoceptor	0.0002025 s^{-1}	This paper
k_{GRK2+}	Rate of GRK2 phosphorylation of β_1-adrenoceptor	0.000243 s^{-1}	This paper
k_{GRK2-}	Rate of GRK2 dephosphorylation of β_1-adrenoceptor	k_{PKA-}	This paper
$k_{act1,Gs}$	Activation rate for G_s by high affinity complex	4.9 s^{-1}	Heijman et al. [8]
$k_{act2,Gs}$	Activation rate for G_s by low affinity complex	0.26 s^{-1}	Heijman et al. [8]
$k_{hyd,Gs}$	Hydrolysis rate of $G_{s\alpha\text{-}GTP}$	0.8 s^{-1}	Saucerman et al. [10]
$k_{reas,Gs}$	Re-association rate for G_s	1200 μM^{-1} s^{-1}	Saucerman et al. [10]

Caveolae

$$[R_{\beta 1}]_{tot}^{cav} = f_{\beta 1}^{cav} \cdot [R_{\beta 1}]_{tot} \cdot \frac{V_{cell}}{V_{cav}} \tag{A.4}$$

$$[G_s]_{\alpha\beta\gamma}^{cav} = f_{Gs}^{cav} \cdot [G_s]_{tot} \cdot \frac{V_{cell}}{V_{cav}} - [G_s]_{\alpha,GTP}^{cav} - [G_s]_{\alpha,GDP}^{cav} \tag{A.5}$$

$$[R_{\beta 1}]_{np,tot}^{cav} = [R_{\beta 1}]_{tot}^{cav} - [R_{\beta 1}]_{PKA,tot}^{cav} - [R_{\beta 1}]_{GRK2,tot}^{cav} \tag{A.6}$$

$$a_{\beta 1}^{cav} = \frac{1}{K_{\beta 1,L}} \cdot \left(K_{\beta 1,L} + [L]\right) \cdot \left(K_{\beta 1,H} + [L]\right) \tag{A.7}$$

$$b_{\beta 1}^{cav} = [G_s]_{\alpha\beta\gamma}^{cav} \cdot \left(K_{\beta 1,H} + [L]\right) - [R_{\beta 1}]_{np,tot}^{cav} \cdot \left(K_{\beta 1,H} + [L]\right) + K_{\beta 1,C} \cdot K_{\beta 1,H} \left(1 + \frac{[L]}{K_{\beta 1,L}}\right) \tag{A.8}$$

$$c_{\beta 1}^{cav} = -[R_{\beta 1}]_{np,tot}^{cav} \cdot K_{\beta 1,C} \cdot K_{\beta 1,H} \tag{A.9}$$

$$[R_{\beta 1}]_{np,f}^{cav} = \frac{-b_{\beta 1}^{cav} + \sqrt{[b_{\beta 1}^{cav}]^2 - 4 \cdot a_{\beta 1}^{cav} \cdot c_{\beta 1}^{cav}}}{2 \cdot a_{\beta 1}^{cav}} \tag{A.10}$$

$$[G_s]_f^{cav} = \frac{[G_s]_{\alpha\beta\gamma}^{cav}}{1+[R_{\beta 1}]_{np,f}^{cav}\left(\frac{1}{K_{\beta 1,C}}+\frac{[L]}{K_{\beta 1,C}\cdot K_{\beta 1,H}}\right)} \quad \text{(A.11)}$$

$$[LR_{\beta 1}]_{np}^{cav} = \frac{[L]\cdot[R_{\beta 1}]_{np,f}^{cav}}{K_{\beta 1,L}} \quad \text{(A.12)}$$

$$[R_{\beta 1}G_s]_{np}^{cav} = \frac{[R_{\beta 1}]_{np,f}^{cav}\cdot[G_s]_f^{cav}}{K_{\beta 1,C}} \quad \text{(A.13)}$$

$$[LR_{\beta 1}G_s]_{np}^{cav} = \frac{[L]\cdot[R_{\beta 1}]_{np,f}^{cav}\cdot[G_s]_f^{cav}}{K_{\beta 1,C}\cdot K_{\beta 1,H}} \quad \text{(A.14)}$$

$$\frac{d[R_{\beta 1}]_{PKA,tot}^{cav}}{dt} = k_{PKA+}\cdot[C]^{cav}\cdot[R_{\beta 1}]_{np,tot}^{cav} - k_{PKA-}\cdot[R_{\beta 1}]_{PKA,tot}^{cav} \quad \text{(A.15)}$$

$$\frac{d[R_{\beta 1}]_{GRK2,tot}^{cav}}{dt} = k_{GRK2+}\cdot\left([LR_{\beta 1}]_{np}^{cav}+[LR_{\beta 1}G_s]_{np}^{cav}\right) - k_{GRK2-}\cdot[R_{\beta 1}]_{GRK2,tot}^{cav} \quad \text{(A.16)}$$

$$\frac{d[G_s]_{\alpha,GTP}^{cav}}{dt} = k_{act2,Gs}\cdot[R_{\beta 1}G_s]_{np}^{cav} + k_{act1,Gs}\cdot[LR_{\beta 1}G_s]_{np}^{cav} - k_{hyd,Gs}\cdot[G_s]_{\alpha,GTP}^{cav} \quad \text{(A.17)}$$

$$\frac{d[G_s]_{\beta\gamma}^{cav}}{dt} = k_{act2,Gs}\cdot[R_{\beta 1}G_s]_{np}^{cav} + k_{act1,Gs}\cdot[LR_{\beta 1}G_s]_{np}^{cav} - k_{reas,Gs}\cdot[G_s]_{\beta\gamma}^{cav}\cdot[G_s]_{\alpha,GDP}^{cav} \quad \text{(A.18)}$$

$$\frac{d[G_s]_{\alpha,GDP}^{cav}}{dt} = k_{hyd,Gs}\cdot[G_s]_{\alpha,GTP}^{cav} - k_{reas,Gs}\cdot[G_s]_{\beta\gamma}^{cav}\cdot[G_s]_{\alpha,GDP}^{cav} \quad \text{(A.19)}$$

Extracaveolae

$$[R_{\beta 1}]_{tot}^{ecav} = \mathrm{f}_{\beta 1}^{ecav}\cdot[R_{\beta 1}]_{tot}\cdot\frac{V_{cell}}{V_{ecav}} \quad \text{(A.20)}$$

$$[G_s]_{\alpha\beta\gamma}^{ecav} = \mathrm{f}_{Gs}^{ecav}\cdot[G_s]_{tot}\cdot\frac{V_{cell}}{V_{ecav}} - [G_s]_{\alpha,GTP}^{ecav} - [G_s]_{\alpha,GDP}^{ecav} \quad \text{(A.21)}$$

$$[R_{\beta 1}]_{np,tot}^{ecav} = [R_{\beta 1}]_{tot}^{ecav} - [R_{\beta 1}]_{PKA,tot}^{ecav} - [R_{\beta 1}]_{GRK2,tot}^{ecav} \quad \text{(A.22)}$$

$$a_{\beta 1}^{ecav} = \frac{1}{K_{\beta 1,L}}\cdot\left(K_{\beta 1,L}+[L]\right)\cdot\left(K_{\beta 1,H}+[L]\right) \quad \text{(A.23)}$$

$$b_{\beta 1}^{ecav} = [G_s]_{\alpha\beta\gamma}^{ecav}\cdot\left(K_{\beta 1,H}+[L]\right) - [R_{\beta 1}]_{np,tot}^{ecav}\cdot\left(K_{\beta 1,H}+[L]\right) + K_{\beta 1,C}\cdot K_{\beta 1,H}\left(1+\frac{[L]}{K_{\beta 1,L}}\right) \quad \text{(A.24)}$$

$$c_{\beta 1}^{ecav} = -[R_{\beta 1}]_{np,tot}^{ecav} \cdot K_{\beta 1,C} \cdot K_{\beta 1,H} \quad \text{(A.25)}$$

$$[R_{\beta 1}]_{np,f}^{ecav} = \frac{-b_{\beta 1}^{ecav} + \sqrt{[b_{\beta 1}^{ecav}]^2 - 4 \cdot a_{\beta 1}^{ecav} \cdot c_{\beta 1}^{ecav}}}{2 \cdot a_{\beta 1}^{ecav}} \quad \text{(A.26)}$$

$$[G_s]_f^{ecav} = \frac{[G_s]_{\alpha\beta\gamma}^{ecav}}{1 + [R_{\beta 1}]_{np,f}^{ecav}\left(\frac{1}{K_{\beta 1,C}} + \frac{[L]}{K_{\beta 1,C} \cdot K_{\beta 1,H}}\right)} \quad \text{(A.27)}$$

$$[LR_{\beta 1}]_{np}^{ecav} = \frac{[L] \cdot [R_{\beta 1}]_{np,f}^{ecav}}{K_{\beta 1,L}} \quad \text{(A.28)}$$

$$[R_{\beta 1}G_s]_{np}^{ecav} = \frac{[R_{\beta 1}]_{np,f}^{ecav} \cdot [G_s]_f^{ecav}}{K_{\beta 1,C}} \quad \text{(A.29)}$$

$$[LR_{\beta 1}G_s]_{np}^{ecav} = \frac{[L] \cdot [R_{\beta 1}]_{np,f}^{ecav} \cdot [G_s]_f^{ecav}}{K_{\beta 1,C} \cdot K_{\beta 1,H}} \quad \text{(A.30)}$$

$$\frac{d[R_{\beta 1}]_{PKA,tot}^{ecav}}{dt} = k_{PKA+} \cdot [C]^{ecav} \cdot [R_{\beta 1}]_{np,tot}^{ecav} - k_{PKA-} \cdot [R_{\beta 1}]_{PKA,tot}^{ecav} \quad \text{(A.31)}$$

$$\frac{d[R_{\beta 1}]_{GRK2,tot}^{ecav}}{dt} = k_{GRK2+} \cdot \left([LR_{\beta 1}]_{np}^{ecav} + [LR_{\beta 1}G_s]_{np}^{ecav}\right) - k_{GRK2-} \cdot [R_{\beta 1}]_{GRK2,tot}^{ecav} \quad \text{(A.32)}$$

$$\frac{d[G_s]_{\alpha,GTP}^{ecav}}{dt} = k_{act2,Gs} \cdot [R_{\beta 1}G_s]_{np}^{ecav} + k_{act1,Gs} \cdot [LR_{\beta 1}G_s]_{np}^{ecav} - k_{hyd,Gs} \cdot [G_s]_{\alpha,GTP}^{ecav} \quad \text{(A.33)}$$

$$\frac{d[G_s]_{\beta\gamma}^{ecav}}{dt} = k_{act2,Gs} \cdot [R_{\beta 1}G_s]_{np}^{ecav} + k_{act1,Gs} \cdot [LR_{\beta 1}G_s]_{np}^{ecav} - k_{reas,Gs} \cdot [G_s]_{\beta\gamma}^{ecav} \cdot [G_s]_{\alpha,GDP}^{ecav} \quad \text{(A.34)}$$

$$\frac{d[G_s]_{\alpha,GDP}^{ecav}}{dt} = k_{hyd,Gs} \cdot [G_s]_{\alpha,GTP}^{ecav} - k_{reas,Gs} \cdot [G_s]_{\beta\gamma}^{ecav} \cdot [G_s]_{\alpha,GDP}^{ecav} \quad \text{(A.35)}$$

Cytosol

$$[R_{\beta 1}]_{tot}^{cyt} = \mathrm{f}_{\beta 1}^{cyt} \cdot [R_{\beta 1}]_{tot} \cdot \frac{V_{cell}}{V_{cyt}} \quad \text{(A.36)}$$

$$[G_s]_{\alpha\beta\gamma}^{cyt} = \mathrm{f}_{Gs}^{cyt} \cdot [G_s]_{tot} \cdot \frac{V_{cell}}{V_{cyt}} - [G_s]_{\alpha,GTP}^{cyt} - [G_s]_{\alpha,GDP}^{cyt} \quad \text{(A.37)}$$

$$[R_{\beta 1}]_{np,tot}^{cyt} = [R_{\beta 1}]_{tot}^{cyt} - [R_{\beta 1}]_{PKA,tot}^{cyt} - [R_{\beta 1}]_{GRK2,tot}^{cyt} \quad \text{(A.38)}$$

$$a_{\beta 1}^{cyt} = \frac{1}{K_{\beta 1,L}} \cdot \left(K_{\beta 1,L} + [L]\right) \cdot \left(K_{\beta 1,H} + [L]\right) \tag{A.39}$$

$$b_{\beta 1}^{cyt} = [G_s]_{\alpha\beta\gamma}^{cyt} \cdot \left(K_{\beta 1,H} + [L]\right) - [R_{\beta 1}]_{np,tot}^{cyt} \cdot \left(K_{\beta 1,H} + [L]\right) + K_{\beta 1,C} \cdot K_{\beta 1,H} \left(1 + \frac{[L]}{K_{\beta 1,L}}\right) \tag{A.40}$$

$$c_{\beta 1}^{cyt} = -[R_{\beta 1}]_{np,tot}^{cyt} \cdot K_{\beta 1,C} \cdot K_{\beta 1,H} \tag{A.41}$$

$$[R_{\beta 1}]_{np,f}^{cyt} = \frac{-b_{\beta 1}^{cyt} + \sqrt{[b_{\beta 1}^{cyt}]^2 - 4 \cdot a_{\beta 1}^{cyt} \cdot c_{\beta 1}^{cyt}}}{2 \cdot a_{\beta 1}^{cyt}} \tag{A.42}$$

$$[G_s]_f^{cyt} = \frac{[G_s]_{\alpha\beta\gamma}^{cyt}}{1 + [R_{\beta 1}]_{np,f}^{cyt} \left(\frac{1}{K_{\beta 1,C}} + \frac{[L]}{K_{\beta 1,C} \cdot K_{\beta 1,H}}\right)} \tag{A.43}$$

$$[LR_{\beta 1}]_{np}^{cyt} = \frac{[L] \cdot [R_{\beta 1}]_{np,f}^{cyt}}{K_{\beta 1,L}} \tag{A.44}$$

$$[R_{\beta 1}G_s]_{np}^{cyt} = \frac{[R_{\beta 1}]_{np,f}^{cyt} \cdot [G_s]_f^{cyt}}{K_{\beta 1,C}} \tag{A.45}$$

$$[LR_{\beta 1}G_s]_{np}^{cyt} = \frac{[L] \cdot [R_{\beta 1}]_{np,f}^{cyt} \cdot [G_s]_f^{cyt}}{K_{\beta 1,C} \cdot K_{\beta 1,H}} \tag{A.46}$$

$$\frac{d[R_{\beta 1}]_{PKA,tot}^{cyt}}{dt} = k_{PKA+} \cdot [C]^{cyt} \cdot [R_{\beta 1}]_{np,tot}^{cyt} - k_{PKA-} \cdot [R_{\beta 1}]_{PKA,tot}^{cyt} \tag{A.47}$$

$$\frac{d[R_{\beta 1}]_{GRK2,tot}^{cyt}}{dt} = k_{GRK2+} \cdot \left([LR_{\beta 1}]_{np}^{cyt} + [LR_{\beta 1}G_s]_{np}^{cyt}\right) - k_{GRK2-} \cdot [R_{\beta 1}]_{GRK2,tot}^{cyt} \tag{A.48}$$

$$\frac{d[G_s]_{\alpha,GTP}^{cyt}}{dt} = k_{act2,Gs} \cdot [R_{\beta 1}G_s]_{np}^{cyt} + k_{act1,Gs} \cdot [LR_{\beta 1}G_s]_{np}^{cyt} - k_{hyd,Gs} \cdot [G_s]_{\alpha,GTP}^{cyt} \tag{A.49}$$

$$\frac{d[G_s]_{\beta\gamma}^{cyt}}{dt} = k_{act2,Gs} \cdot [R_{\beta 1}G_s]_{np}^{cyt} + k_{act1,Gs} \cdot [LR_{\beta 1}G_s]_{np}^{cyt} - k_{reas,Gs} \cdot [G_s]_{\beta\gamma}^{cyt} \cdot [G_s]_{\alpha,GDP}^{cyt} \tag{A.50}$$

$$\frac{d[G_s]_{\alpha,GDP}^{cyt}}{dt} = k_{hyd,Gs} \cdot [G_s]_{\alpha,GTP}^{cyt} - k_{reas,Gs} \cdot [G_s]_{\beta\gamma}^{cyt} \cdot [G_s]_{\alpha,GDP}^{cyt} \tag{A.51}$$

Adenylyl cyclase module

Parameter	Definition	Value	Reference
$K_{m,ATP}$	Adenylyl cyclase affinity for ATP	340 μM	This paper
$[ATP]$	ATP concentration	5000 μM	Heijman et al. [8]
$[AC]_{tot}$	Total cellular AC concentration	0.02622 μM	Post et al. [41]
$\mathrm{f}_{AC56,AC47}$	Fraction of AC that is of type 5 or 6	0.74	Heijman et al. [8]
f_{AC56}^{cav}	Fraction of AC5/6 located in caveolae	0.0875	Heijman et al. [8]
f_{AC47}^{ecav}	Fraction of AC4/7 located in extracaveolae space	0.1648	Heijman et al. [8]
$K_{m,Gs\alpha}^{AC56}$	AC5/6 affinity for $G_{s\alpha}$	0.0852 μM	Heijman et al. [8]
$h_{AC56,Gs\alpha}$	Hill coefficient for AC5/6 activation by $G_{s\alpha}$	1.357	Heijman et al. [8]
$V_{G\beta\gamma}^{AC56}$	Maximum amplification of AC5/6 by $G_{s\beta\gamma}$	1.430	Gao et al. [39]
$K_{m,Gs\beta\gamma}^{AC56}$	Affinity constant for $G_{s\beta\gamma}$ modulation of AC5/6	0.003793 μM	Gao et al. [39]
$h_{AC56,Gs\beta\gamma}$	Hill coefficient for $G_{s\beta\gamma}$ modulation of AC5/6	1.0842	Gao et al. [39]
$AC56_{basal}$	Basal AC5/6 activity	0.0377	Heijman et al. [8]
AF_{56}	Amplification factor for AC5/6	51.1335 s^{-1}	This paper
$K_{m,Gs\alpha}^{AC47}$	AC4/7 affinity for $G_{s\alpha}$	0.05008 μM	Zimmermann and Taussig [40]
$h_{AC47,Gs\alpha}$	Hill coefficient for AC4/7 activation by $G_{s\alpha}$	1.1657	Zimmermann and Taussig [40]
$V_{G\beta\gamma}^{AC47}$	Maximum amplification of AC4/7 by $G_{s\beta\gamma}$	1.3500	Zimmermann and Taussig [40]
$K_{m,Gs\beta\gamma}^{AC47}$	Affinity constant for $G_{s\beta\gamma}$ modulation of AC4/7	0.004466 μM	Zimmermann and Taussig [40]
$h_{AC47,Gs\beta\gamma}$	Hill coefficient for $G_{s\beta\gamma}$ modulation of AC4/7	0.8700	Zimmermann and Taussig [40]
$AC47_{basal}$	Basal AC4/7 activity	0.04725	This paper
AF_{47}	Amplification factor for AC4/7	9.283 s^{-1}	This paper

Caveolae

$$[AC56]^{cav} = \mathrm{f}_{AC56}^{cav} \cdot \mathrm{f}_{AC56,AC47} \cdot [AC]_{tot} \cdot \frac{V_{cell}}{V_{cav}} \tag{A.52}$$

$$k_{AC56}^{cav} = AF_{56} \cdot \left(AC56_{basal} + \frac{\left([G_s]_{\alpha,GTP}^{cav}\right)^{h_{AC56,Gs\alpha}}}{K_{m,Gs\alpha}^{AC56} + \left([G_s]_{\alpha,GTP}^{cav}\right)^{h_{AC56,Gs\alpha}}} \right) \cdot \left(1 + \frac{V_{G\beta\gamma}^{AC56} \cdot \left([G_s]_{\beta\gamma}^{cav}\right)^{h_{AC56,Gs\beta\gamma}}}{K_{m,Gs\beta\gamma}^{AC56} + \left([G_s]_{\beta\gamma}^{cav}\right)^{h_{AC56,Gs\beta\gamma}}} \right) \tag{A.53}$$

$$\frac{d[cAMP]_{AC56}^{cav}}{dt} = k_{AC56}^{cav} \cdot \frac{[AC56]^{cav} \cdot [ATP]}{K_{m,ATP} + [ATP]} \tag{A.54}$$

Extracaveolae

$$[AC47]^{ecav} = \mathrm{f}_{AC47}^{ecav} \cdot (1 - \mathrm{f}_{AC56,AC47}) \cdot [AC]_{tot} \cdot \frac{V_{cell}}{V_{ecav}} \tag{A.55}$$

$$k_{AC47}^{ecav} = AF_{47} \cdot \left(AC47_{basal} + \frac{\left([G_s]_{\alpha,GTP}^{ecav}\right)^{h_{AC47,Gs\alpha}}}{K_{m,Gs\alpha}^{AC47} + \left([G_s]_{\alpha,GTP}^{ecav}\right)^{h_{AC47,Gs\alpha}}} \right) \cdot \left(1 + \frac{V_{G\beta\gamma}^{AC47} \cdot \left([G_s]_{\beta\gamma}^{ecav}\right)^{h_{AC47,Gs\beta\gamma}}}{K_{m,Gs\beta\gamma}^{AC47} + \left([G_s]_{\beta\gamma}^{ecav}\right)^{h_{AC47,Gs\beta\gamma}}} \right) \tag{A.56}$$

$$\frac{d[cAMP]_{AC47}^{ecav}}{dt} = k_{AC47}^{ecav} \cdot \frac{[AC47]^{ecav} \cdot [ATP]}{K_{m,ATP} + [ATP]} \tag{A.57}$$

Cytosol

$$[AC56]^{cyt} = (1 - \mathrm{f}_{AC56}^{cav}) \cdot \mathrm{f}_{AC56,AC47} \cdot [AC]_{tot} \cdot \frac{V_{cell}}{V_{cyt}} \tag{A.58}$$

$$[AC47]^{cyt} = (1 - \mathrm{f}_{AC47}^{ecav}) \cdot (1 - \mathrm{f}_{AC56,AC47}) \cdot [AC]_{tot} \cdot \frac{V_{cell}}{V_{cyt}} \tag{A.59}$$

$$k_{AC56}^{cyt} = AF_{56} \cdot \left(AC56_{basal} + \frac{\left([G_s]_{\alpha,GTP}^{cyt}\right)^{h_{AC56,Gs\alpha}}}{K_{m,Gs\alpha}^{AC56} + \left([G_s]_{\alpha,GTP}^{cyt}\right)^{h_{AC56,Gs\alpha}}} \right) \cdot \left(1 + \frac{V_{G\beta\gamma}^{AC56} \cdot \left([G_s]_{\beta\gamma}^{cyt}\right)^{h_{AC56,Gs\beta\gamma}}}{K_{m,Gs\beta\gamma}^{AC56} + \left([G_s]_{\beta\gamma}^{cyt}\right)^{h_{AC56,Gs\beta\gamma}}} \right) \tag{A.60}$$

$$\frac{d[cAMP]_{AC56}^{cyt}}{dt} = k_{AC56}^{cyt} \cdot \frac{[AC56]^{cyt} \cdot [ATP]}{K_{m,ATP} + [ATP]} \tag{A.61}$$

$$k_{AC47}^{cyt} = AF_{47} \cdot \left(AC47_{basal} + \frac{\left([G_s]_{\alpha,GTP}^{cyt}\right)^{h_{AC47,Gs\alpha}}}{K_{m,Gs\alpha}^{AC47} + \left([G_s]_{\alpha,GTP}^{cyt}\right)^{h_{AC47,Gs\alpha}}} \right) \cdot \left(1 + \frac{V_{G\beta\gamma}^{AC47} \cdot \left([G_s]_{\beta\gamma}^{cyt}\right)^{h_{AC47,Gs\beta\gamma}}}{K_{m,Gs\beta\gamma}^{AC47} + \left([G_s]_{\beta\gamma}^{cyt}\right)^{h_{AC47,Gs\beta\gamma}}} \right) \tag{A.62}$$

$$\frac{d[cAMP]_{AC47}^{cyt}}{dt} = k_{AC47}^{cyt} \cdot \frac{[AC47]^{cyt} \cdot [ATP]}{K_{m,ATP} + [ATP]} \tag{A.63}$$

Phosphodiesterase module

Parameter	Definition	Value	Reference
$[IBMX]$	Concentration of IBMX	0…100 μM	
$h_{IBMX,PDE2}$	Hill coefficient for inhibition of PDE2 by IBMX	1.000	This paper
K_{PDE2}^{IBMX}	Affinity of IBMX for PDE2	29.50 μM	This paper
$h_{IBMX,PDE3}$	Hill coefficient for inhibition of PDE3 by IBMX	1.000	This paper
K_{PDE3}^{IBMX}	Affinity of IBMX for PDE3	5.100 μM	This paper
$h_{IBMX,PDE4}$	Hill coefficient for inhibition of PDE4 by IBMX	1.000	This paper
K_{PDE4}^{IBMX}	Affinity of IBMX for PDE4	16.200 μM	This paper
$k_{f,PDEp}$	Rate of phosphorylation of PDE3/4 by PKA	0.0196 μM^{-1} s^{-1}	Heijman et al. [8]
$k_{b,PDEp}$	Rate of dephosphorylation of PDE3/4 by PKA	0.0102 s^{-1}	Heijman et al. [8]
$\Delta_{k,PDE3/4}$	Increase in PDE3/4 activity after phosphorylation	3.0	Heijman et al. [8]
k_{PDE2}	Rate of cAMP hydrolysis by PDE2	20 s^{-1}	Iancu et al. [14]
$K_{m,PDE2}$	Affinity of PDE2 for cAMP	33 μM	Bode et al. [45]
k_{PDE3}	Rate of cAMP hydrolysis by PDE3	2.5 s^{-1}	Heijman et al. [8]
$K_{m,PDE3}$	Affinity of PDE3 for cAMP	0.44 μM	Bode et al. [45]
k_{PDE4}	Rate of cAMP hydrolysis by PDE4	3.5 s^{-1}	This paper
$K_{m,PDE4}$	Affinity of PDE4 for cAMP	1.4 μM	This paper
$f_{PDE,part}$	Fraction of total PDE located in the particulate fraction	0.2	Osadchii [47]
$r_{part,PDE2,PDE3}$	Ratio of PDE2 and PDE3 activities in particulate fraction	0.570	Mongillo et al. [48]
$r_{part,PDE3,PDE4}$	Ratio of PDE3 and PDE4 activities in particulate fraction	0.748	Mongillo et al. [48]
$[PDE2]_{tot}$	Total cellular concentration of PDE2	0.034610 μM	This paper
$[PDE3]_{tot}$	Total cellular concentration of PDE3	0.010346 μM	This paper
$[PDE4]_{tot}$	Total cellular concentration of PDE4	0.026687 μM	This paper
f_{PDE2}^{cav}	Fraction of PDE2 located in caveolae	0.06608	This paper
f_{PDE2}^{ecav}	Fraction of PDE2 located in extracaveolae	$2 \cdot f_{PDE2}^{cav}$	This paper
f_{PDE2}^{cyt}	Fraction of PDE2 located in cytosol	$1 - f_{PDE2}^{cav} - f_{PDE2}^{ecav}$	This paper
f_{PDE3}^{cav}	Fraction of PDE3 located in caveolae	0.29814	This paper
f_{PDE3}^{ecav}	Fraction of PDE3 located in extracaveolae	0.0	This paper
f_{PDE3}^{cyt}	Fraction of PDE3 located in cytosol	$1 - f_{PDE3}^{cav} - f_{PDE3}^{ecav}$	This paper
f_{PDE4}^{cav}	Fraction of PDE4 located in caveolae	0.05366	This paper

f_{PDE4}^{ecav}	Fraction of PDE4 located in extracaveolae	$2 \cdot f_{PDE4}^{cav}$	This paper
f_{PDE4}^{cyt}	Fraction of PDE4 located in cytosol	$1 - f_{PDE4}^{cav} - f_{PDE4}^{ecav}$	This paper

Caveolae

$$[PDE2]_{tot}^{cav} = \left(1 - \frac{[IBMX]^{h_{IBMX,PDE2}}}{K_{m,PDE2}^{IBMX} + [IBMX]^{h_{IBMX,PDE2}}}\right) \cdot f_{PDE2}^{cav} \cdot [PDE2]_{tot} \cdot \frac{V^{cell}}{V^{cav}} \quad \text{(A.64)}$$

$$[PDE3]_{tot}^{cav} = \left(1 - \frac{[IBMX]^{h_{IBMX,PDE3}}}{K_{m,PDE3}^{IBMX} + [IBMX]^{h_{IBMX,PDE3}}}\right) \cdot f_{PDE3}^{cav} \cdot [PDE3]_{tot} \cdot \frac{V^{cell}}{V^{cav}} \quad \text{(A.65)}$$

$$[PDE4]_{tot}^{cav} = \left(1 - \frac{[IBMX]^{h_{IBMX,PDE4}}}{K_{m,PDE4}^{IBMX} + [IBMX]^{h_{IBMX,PDE4}}}\right) \cdot f_{PDE4}^{cav} \cdot [PDE4]_{tot} \cdot \frac{V^{cell}}{V^{cav}} \quad \text{(A.66)}$$

$$\frac{d[PDE3]_p^{cav}}{dt} = k_{f,PDEp} \cdot [C]^{cav} \cdot ([PDE3]_{tot}^{cav} - [PDE3]_p^{cav}) - k_{b,PDEp} \cdot [PDE3]_p^{cav} \quad \text{(A.67)}$$

$$\frac{d[PDE4]_p^{cav}}{dt} = k_{f,PDEp} \cdot [C]^{cav} \cdot ([PDE4]_{tot}^{cav} - [PDE4]_p^{cav}) - k_{b,PDEp} \cdot [PDE4]_p^{cav} \quad \text{(A.68)}$$

$$\frac{d[cAMP]_{PDE2}^{cav}}{dt} = \frac{k_{PDE2} \cdot [PDE2]_{tot}^{cav} \cdot [cAMP]^{cav}}{K_{m,PDE2} + [cAMP]^{cav}} \quad \text{(A.69)}$$

$$\frac{d[cAMP]_{PDE3}^{cav}}{dt} = \frac{k_{PDE3} \cdot ([PDE3]_{tot}^{cav} - [PDE3]_p^{cav}) \cdot [cAMP]^{cav} + \Delta_{k,PDE3/4} \cdot k_{PDE3} \cdot [PDE3]_p^{cav} \cdot [cAMP]^{cav}}{K_{m,PDE3} + [cAMP]^{cav}} \quad \text{(A.70)}$$

$$\frac{d[cAMP]_{PDE4}^{cav}}{dt} = \frac{k_{PDE4} \cdot ([PDE4]_{tot}^{cav} - [PDE4]_p^{cav}) \cdot [cAMP]^{cav} + \Delta_{k,PDE3/4} \cdot k_{PDE4} \cdot [PDE4]_p^{cav} \cdot [cAMP]^{cav}}{K_{m,PDE4} + [cAMP]^{cav}} \quad \text{(A.71)}$$

Extracaveolae

$$[PDE2]_{tot}^{ecav} = \left(1 - \frac{[IBMX]^{h_{IBMX,PDE2}}}{K_{m,PDE2}^{IBMX} + [IBMX]^{h_{IBMX,PDE2}}}\right) \cdot f_{PDE2}^{ecav} \cdot [PDE2]_{tot} \cdot \frac{V^{cell}}{V^{ecav}} \quad \text{(A.72)}$$

$$[PDE4]_{tot}^{ecav} = \left(1 - \frac{[IBMX]^{h_{IBMX,PDE4}}}{K_{m,PDE4}^{IBMX} + [IBMX]^{h_{IBMX,PDE4}}}\right) \cdot f_{PDE4}^{ecav} \cdot [PDE4]_{tot} \cdot \frac{V^{cell}}{V^{ecav}} \quad \text{(A.73)}$$

$$\frac{d[PDE4]_p^{ecav}}{dt} = k_{f,PDEp} \cdot [C]^{ecav} \cdot ([PDE4]_{tot}^{ecav} - [PDE4]_p^{ecav}) - k_{b,PDEp} \cdot [PDE4]_p^{ecav} \quad \text{(A.74)}$$

$$\frac{d[cAMP]_{PDE2}^{ecav}}{dt} = \frac{k_{PDE2} \cdot [PDE2]_{tot}^{ecav} \cdot [cAMP]^{ecav}}{K_{m,PDE2} + [cAMP]^{ecav}} \quad \text{(A.75)}$$

$$\frac{d[cAMP]_{PDE4}^{ecav}}{dt} = \frac{k_{PDE4} \cdot ([PDE4]_{tot}^{ecav} - [PDE4]_p^{ecav}) \cdot [cAMP]^{ecav} + \Delta_{k,PDE3/4} \cdot k_{PDE4} \cdot [PDE4]_p^{ecav} \cdot [cAMP]^{ecav}}{K_{m,PDE4} + [cAMP]^{ecav}} \quad \text{(A.76)}$$

Cytosol

$$[PDE2]_{tot}^{cyt} = \left(1 - \frac{[IBMX]^{h_{IBMX,PDE2}}}{K_{m,PDE2}^{IBMX} + [IBMX]^{h_{IBMX,PDE2}}}\right) \cdot \mathrm{f}_{PDE2}^{cyt} \cdot [PDE2]_{tot} \cdot \frac{V^{cell}}{V^{cyt}} \tag{A.77}$$

$$[PDE3]_{tot}^{cyt} = \left(1 - \frac{[IBMX]^{h_{IBMX,PDE3}}}{K_{m,PDE3}^{IBMX} + [IBMX]^{h_{IBMX,PDE3}}}\right) \cdot \mathrm{f}_{PDE3}^{cyt} \cdot [PDE3]_{tot} \cdot \frac{V^{cell}}{V^{cyt}} \tag{A.78}$$

$$[PDE4]_{tot}^{cyt} = \left(1 - \frac{[IBMX]^{h_{IBMX,PDE4}}}{K_{m,PDE4}^{IBMX} + [IBMX]^{h_{IBMX,PDE4}}}\right) \cdot \mathrm{f}_{PDE4}^{cyt} \cdot [PDE4]_{tot} \cdot \frac{V^{cell}}{V^{cyt}} \tag{A.79}$$

$$\frac{d[PDE3]_p^{cyt}}{dt} = k_{f,PDEp} \cdot [C]^{cyt} \cdot ([PDE3]_{tot}^{cyt} - [PDE3]_p^{cyt}) - k_{b,PDEp} \cdot [PDE3]_p^{cyt} \tag{A.80}$$

$$\frac{d[PDE4]_p^{cyt}}{dt} = k_{f,PDEp} \cdot [C]^{cyt} \cdot ([PDE4]_{tot}^{cyt} - [PDE4]_p^{cyt}) - k_{b,PDEp} \cdot [PDE4]_p^{cyt} \tag{A.81}$$

$$\frac{d[cAMP]_{PDE2}^{cyt}}{dt} = \frac{k_{PDE2} \cdot [PDE2]_{tot}^{cyt} \cdot [cAMP]^{cyt}}{K_{m,PDE2} + [cAMP]^{cyt}} \tag{A.82}$$

$$\frac{d[cAMP]_{PDE3}^{cyt}}{dt} = \frac{k_{PDE3} \cdot ([PDE3]_{tot}^{cyt} - [PDE3]_p^{cyt}) \cdot [cAMP]^{cyt} + \Delta_{k,PDE3/4} \cdot k_{PDE3} \cdot [PDE3]_p^{cyt} \cdot [cAMP]^{cyt}}{K_{m,PDE3} + [cAMP]^{cyt}} \tag{A.83}$$

$$\frac{d[cAMP]_{PDE4}^{cyt}}{dt} = \frac{k_{PDE4} \cdot ([PDE4]_{tot}^{cyt} - [PDE4]_p^{cyt}) \cdot [cAMP]^{cyt} + \Delta_{k,PDE3/4} \cdot k_{PDE4} \cdot [PDE4]_p^{cyt} \cdot [cAMP]^{cyt}}{K_{m,PDE4} + [cAMP]^{cyt}} \tag{A.84}$$

cAMP-PKA module

Parameter	Definition	Value	Reference
$[PKA]_{tot}$	Total cellular concentration of PKA holoenzyme	0.5176 µM	Corbin et al., J Biol Chem 252: 3854-3861, 1977 [149]
f_{PKA}^{cav}	Fraction of PKA located in caveolae	0.08	This paper
f_{PKA}^{ecav}	Fraction of PKA located in extracaveolae	0.20	This paper
f_{PKA}^{cyt}	Fraction of PKA located in cytosol	$1 - \mathrm{f}_{PKA}^{cav} - \mathrm{f}_{PKA}^{ecav}$	
$[PKI]_{tot}$	Total cellular concentration of PKA inhibitor	$2 \cdot 0.2 \cdot [PKA]_{tot}$	Beavo et al. [54]
f_{PKI}^{cav}	Fraction of PKI located in caveolae	f_{PKA}^{cav}	
f_{PKI}^{ecav}	Fraction of PKI located in extracaveolae	f_{PKA}^{ecav}	
f_{PKI}^{cyt}	Fraction of PKI located in cytosol	f_{PKA}^{cyt}	
$k_{PKAI,f1}$	Forward rate for binding of the first cAMP to PKA	5.6 µM^{-1} s^{-1}	This paper

$K_{PKAI,1}$	Equilibrium value for the binding of the first cAMP to PKA	2.9 μM	Dao et al. [55]
$k_{PKAI,f2}$	Forward rate for binding of the second cAMP to PKA	$k_{PKAI,f1}$	This paper
$K_{PKAI,2}$	Equilibrium value for binding of the second cAMP to PKA	2.9 μM	Dao et al. [55]
$k_{PKAI,f3}$	Forward rate for dissociation of C subunit	2.6 s^{-1}	This paper
$K_{PKAI,3}$	Equilibrium value for dissociation of C subunit	1.3 μM	This paper
$k_{PKI,f}$	Forward rate for inhibition of C subunit by PKI	50 μM^{-1} s^{-1}	Heijman et al. [8]
K_{PKI}	Equilibrium value for inhibition of C subunit by PKI	$2.6 \cdot 10^{-4}$ μM	This paper
$k_{PKAII,f1}$	Forward rate for binding of the first cAMP to PKA	$k_{PKAI,f1}$	Heijman et al. [8]
$K_{PKAII,1}$	Equilibrium value for the binding of the first cAMP to PKA	2.5 μM	Heijman et al. [8]
$k_{PKAII,f2}$	Forward rate for binding of the second cAMP to PKA	$k_{PKAI,f1}$	Heijman et al. [8]
$K_{PKAII,2}$	Equilibrium value for binding of the second cAMP to PKA	2.5 μM	Heijman et al. [8]
$k_{PKAII,f3}$	Forward rate for dissociation of C subunit	$k_{PKAI,f3}$	This paper
$K_{PKAII,3}$	Equilibrium value for dissociation of C subunit	$K_{PKAI,3}$	This paper

Caveolae

$$[PKA]^{cav} = \mathrm{f}_{PKA}^{cav} \cdot [PKA]_{tot} \cdot \frac{V^{cell}}{V^{cav}} \quad \text{(A.85)}$$

$$[RC]_f^{cav} = 2 \cdot [PKA]^{cav} - [ARC]^{cav} - [A_2RC]^{cav} - [A_2R]^{cav} \quad \text{(A.86)}$$

$$[PKI]_f^{cav} = \mathrm{f}_{PKI}^{cav} \cdot [PKI]_{tot} \cdot \frac{V^{cell}}{V^{cav}} - [PKIC]^{cav} \quad \text{(A.87)}$$

$$k_{PKAII,b1} = k_{PKAII,f1} \cdot K_{PKAII,1} \quad \text{(A.88)}$$

$$k_{PKAII,b2} = k_{PKAII,f2} \cdot K_{PKAII,2} \quad \text{(A.89)}$$

$$k_{PKAII,b3} = k_{PKAII,f3} / K_{PKAII,3} \quad \text{(A.90)}$$

$$k_{PKI,b} = k_{PKI,f} \cdot K_{PKI} \quad \text{(A.91)}$$

$$\frac{d[cAMP]_{PKA}^{cav}}{dt} = -k_{PKAII,f1} \cdot [RC]_f^{cav} \cdot [cAMP]^{cav} + k_{PKAII,b1} \cdot [ARC]^{cav} - k_{PKAII,f2} \cdot [ARC]^{cav} \cdot [cAMP]^{cav}$$
$$+ k_{PKAII,b2} \cdot [A_2RC]^{cav} \quad \text{(A.92)}$$

$$\frac{d[ARC]^{cav}}{dt} = k_{PKAII,f1} \cdot [RC]_f^{cav} \cdot [cAMP]^{cav} - k_{PKAII,b1} \cdot [ARC]^{cav} - k_{PKAII,f2} \cdot [ARC]^{cav} \cdot [cAMP]^{cav}$$
$$+k_{PKAII,b2} \cdot [A_2RC]^{cav} \quad \text{(A.93)}$$

$$\frac{d[A_2RC]^{cav}}{dt} = k_{PKAII,f2} \cdot [ARC]^{cav} \cdot [cAMP]^{cav} - (k_{PKAII,b2} + k_{PKAII,f3}) \cdot [A_2RC]^{cav}$$
$$+k_{PKAII,b3} \cdot [A_2R]^{cav} \cdot [C]^{cav} \quad \text{(A.94)}$$

$$\frac{d[A_2R]^{cav}}{dt} = k_{PKAII,f3} \cdot [A_2RC]^{cav} - k_{PKAII,b3} \cdot [A_2R]^{cav} \cdot [C]^{cav} \quad \text{(A.95)}$$

$$\frac{d[C]^{cav}}{dt} = k_{PKAII,f3} \cdot [A_2RC]^{cav} - k_{PKAII,b3} \cdot [A_2R]^{cav} \cdot [C]^{cav} + k_{PKI,b} \cdot [PKIC]^{cav} - k_{PKI,f} \cdot [PKI]_f^{cav} \cdot [C]^{cav} \quad \text{(A.96)}$$

$$\frac{d[PKIC]^{cav}}{dt} = -k_{PKI,b} \cdot [PKIC]^{cav} + k_{PKI,f} \cdot [PKI]_f^{cav} \cdot [C]^{cav} \quad \text{(A.97)}$$

Extracaveolae

$$[PKA]^{ecav} = \mathrm{f}_{PKA}^{ecav} \cdot [PKA]_{tot} \cdot \frac{V^{cell}}{V^{ecav}} \quad \text{(A.98)}$$

$$[RC]_f^{ecav} = 2 \cdot [PKA]^{ecav} - [ARC]^{ecav} - [A_2RC]^{ecav} - [A_2R]^{ecav} \quad \text{(A.99)}$$

$$[PKI]_f^{ecav} = \mathrm{f}_{PKI}^{ecav} \cdot [PKI]_{tot} \cdot \frac{V^{cell}}{V^{ecav}} - [PKIC]^{ecav} \quad \text{(A.100)}$$

$$k_{PKAII,b1} = k_{PKAII,f1} \cdot K_{PKAII,1} \quad \text{(A.101)}$$

$$k_{PKAII,b2} = k_{PKAII,f2} \cdot K_{PKAII,2} \quad \text{(A.102)}$$

$$k_{PKAII,b3} = k_{PKAII,f3} / K_{PKAII,3} \quad \text{(A.103)}$$

$$k_{PKI,b} = k_{PKI,f} \cdot K_{PKI} \quad \text{(A.104)}$$

$$\frac{d[cAMP]_{PKA}^{ecav}}{dt} = -k_{PKAII,f1} \cdot [RC]_f^{ecav} \cdot [cAMP]^{ecav} + k_{PKAII,b1} \cdot [ARC]^{ecav} - k_{PKAII,f2} \cdot [ARC]^{ecav} \cdot [cAMP]^{ecav}$$
$$+k_{PKAII,b2} \cdot [A_2RC]^{ecav} \quad \text{(A.105)}$$

$$\frac{d[ARC]^{ecav}}{dt} = k_{PKAII,f1} \cdot [RC]_f^{ecav} \cdot [cAMP]^{ecav} - k_{PKAII,b1} \cdot [ARC]^{ecav} - k_{PKAII,f2} \cdot [ARC]^{ecav} \cdot [cAMP]^{ecav}$$
$$+k_{PKAII,b2} \cdot [A_2RC]^{ecav} \quad \text{(A.106)}$$

$$\frac{d[A_2RC]^{ecav}}{dt} = k_{PKAII,f2} \cdot [ARC]^{ecav} \cdot [cAMP]^{ecav} - (k_{PKAII,b2} + k_{PKAII,f3}) \cdot [A_2RC]^{ecav}$$
$$+k_{PKAII,b3} \cdot [A_2R]^{ecav} \cdot [C]^{ecav} \quad \text{(A.107)}$$

$$\frac{d[A_2R]^{ecav}}{dt} = k_{PKAII,f3} \cdot [A_2RC]^{ecav} - k_{PKAII,b3} \cdot [A_2R]^{ecav} \cdot [C]^{ecav} \quad \text{(A.108)}$$

$$\frac{d[C]^{ecav}}{dt} = k_{PKAII,f3} \cdot [A_2RC]^{ecav} - k_{PKAII,b3} \cdot [A_2R]^{ecav} \cdot [C]^{ecav} + k_{PKI,b} \cdot [PKIC]^{ecav} - k_{PKI,f} \cdot [PKI]_f^{ecav} \cdot [C]^{ecav} \quad \text{(A.109)}$$

$$\frac{d[PKIC]^{ecav}}{dt} = -k_{PKI,b} \cdot [PKIC]^{ecav} + k_{PKI,f} \cdot [PKI]_f^{ecav} \cdot [C]^{ecav} \quad \text{(A.110)}$$

Cytosol

$$[PKA]^{cyt} = \mathrm{f}_{PKA}^{cyt} \cdot [PKA]_{tot} \cdot \frac{V^{cell}}{V^{cyt}} \quad \text{(A.111)}$$

$$[RC]_f^{cyt} = 2 \cdot [PKA]^{cyt} - [ARC]^{cyt} - [A_2RC]^{cyt} - [A_2R]^{cyt} \quad \text{(A.112)}$$

$$[PKI]_f^{cyt} = \mathrm{f}_{PKI}^{cyt} \cdot [PKI]_{tot} \cdot \frac{V^{cell}}{V^{cyt}} - [PKIC]^{cyt} \quad \text{(A.113)}$$

$$k_{PKAI,b1} = k_{PKAI,f1} \cdot K_{PKAI,1} \quad \text{(A.114)}$$

$$k_{PKAI,b2} = k_{PKAI,f2} \cdot K_{PKAI,2} \quad \text{(A.115)}$$

$$k_{PKAI,b3} = k_{PKAI,f3} / K_{PKAI,3} \quad \text{(A.116)}$$

$$k_{PKI,b} = k_{PKI,f} \cdot K_{PKI} \quad \text{(A.117)}$$

$$\frac{d[cAMP]_{PKA}^{cyt}}{dt} = -k_{PKAI,f1} \cdot [RC]_f^{cyt} \cdot [cAMP]^{cyt} + k_{PKAI,b1} \cdot [ARC]^{cyt} - k_{PKAI,f2} \cdot [ARC]^{cyt} \cdot [cAMP]^{cyt}$$
$$+ k_{PKAI,b2} \cdot [A_2RC]^{cyt} \quad \text{(A.118)}$$

$$\frac{d[ARC]^{cyt}}{dt} = k_{PKAI,f1} \cdot [RC]_f^{cyt} \cdot [cAMP]^{cyt} - k_{PKAI,b1} \cdot [ARC]^{cyt} - k_{PKAI,f2} \cdot [ARC]^{cyt} \cdot [cAMP]^{cyt}$$
$$+ k_{PKAI,b2} \cdot [A_2RC]^{cyt} \quad \text{(A.119)}$$

$$\frac{d[A_2RC]^{cyt}}{dt} = k_{PKAI,f2} \cdot [ARC]^{cyt} \cdot [cAMP]^{cyt} - (k_{PKAI,b2} + k_{PKAI,f3}) \cdot [A_2RC]^{cyt} + k_{PKAI,b3} \cdot [A_2R]^{cyt} \cdot [C]^{cyt} \quad \text{(A.120)}$$

$$\frac{d[A_2R]^{cyt}}{dt} = k_{PKAI,f3} \cdot [A_2RC]^{cyt} - k_{PKAI,b3} \cdot [A_2R]^{cyt} \cdot [C]^{cyt} \quad \text{(A.121)}$$

$$\frac{d[C]^{cyt}}{dt} = k_{PKAI,f3} \cdot [A_2RC]^{cyt} - k_{PKAI,b3} \cdot [A_2R]^{cyt} \cdot [C]^{cyt} + k_{PKI,b} \cdot [PKIC]^{cyt} - k_{PKI,f} \cdot [PKI]_f^{cyt} \cdot [C]^{cyt} \quad \text{(A.122)}$$

$$\frac{d[PKIC]^{cyt}}{dt} = -k_{PKI,b} \cdot [PKIC]^{cyt} + k_{PKI,f} \cdot [PKI]_f^{cyt} \cdot [C]^{cyt} \quad \text{(A.123)}$$

Protein phosphatases and inhibitor-1 module

Parameter	Definition	Value	Reference
$[PP1]_{tot}^{cyt}$	Total concentration of PP1 in the cytosolic compartment	0.2 μM	Heijman et al. [8]
$[PP2A]^{cyt}$	Cytosolic concentration of PP2A	0.0607843 μM	This paper
$[PP1]^{cav}$	Concentration of PP1 in the caveolae compartment	0.1 μM	This paper
$[PP2A]^{cav}$	Concentration of PP2A in the caveolae compartment	0.1 μM	This paper
$[PP]^{cav}$	Total phosphatase concentration in caveolae compartment $[PP1]^{cav}+[PP2A]^{cav}$	0.2 μM	This paper
$[PP1]^{ecav}$	Concentration of PP1 in the extracaveolae compartment	0.1 μM	Heijman et al. [8]
$[Inhib1]_{tot}^{cyt}$	Total concentration of inhibitor 1 in the cytosolic compartment	0.08543 μM	El-Armouche et al. [65]
K_{inhib1}	Affinity for PP1 – Inhibitor 1 binding	$1.0\cdot 10^{-3}$ μM	Saucerman et al. [10]
k_{PKA_Inhib1}	Rate of phosphorylation of inhibitor 1 by PKA	1080.0 $\mu M^{-1}\ s^{-1}$	This paper
K_{mPKA_Inhib1}	Affinity of inhibitor 1 for PKA catalytic subunit	1.5 μM	This paper
k_{PP2A_Inhib1}	Rate of dephosphorylation of inhibitor 1	50.67 $\mu M^{-1}\ s^{-1}$	This paper
K_{mPP2A_Inhib1}	Affinity for PP2A – Inhibitor 1 binding	$1.0\cdot 10^{-3}$ μM	This paper

$$[Inhib1]_f^{cyt} = [Inhib1]_{tot}^{cyt} - [Inhib1]_{p,tot}^{cyt} \quad \text{(A.124)}$$

$$a_{Inhib1} = 1.0 \quad \text{(A.125)}$$

$$b_{Inhib1} = K_{Inhib1} + [PP1]_{tot}^{cyt} - [Inhib1]_{p,tot}^{cyt} \quad \text{(A.126)}$$

$$c_{Inhib1} = -[Inhib1]_{p,tot}^{cyt} \cdot K_{Inhib1} \quad \text{(A.127)}$$

$$[Inhib1]_p^{cyt} = \frac{-b_{Inhib1} + \sqrt{[b_{Inhib1}]^2 - 4\cdot a_{Inhib1}\cdot c_{Inhib1}}}{2\cdot a_{Inhib1}} \quad \text{(A.128)}$$

$$[PP1]_f^{cyt} = \frac{[PP1]_{tot}^{cyt}\cdot K_{Inhib1}}{K_{Inhib1} + [Inhib1]_p^{cyt}} \quad \text{(A.129)}$$

$$\frac{d[Inhib1]_{p,tot}^{cyt}}{dt} = \frac{k_{PKA\_Inhib1}\cdot [C]^{cyt}\cdot [Inhib1]_f^{cyt}}{K_{mPKA\_Inhib1} + [Inhib1]_f^{cyt}} - \frac{k_{PP2A\_Inhib1}\cdot [PP2A]^{cyt}\cdot [Inhib1]_{p,tot}^{cyt}}{K_{mPP2A\_Inhib1} + [Inhib1]_{p,tot}^{cyt}} \quad \text{(A.130)}$$

cAMP fluxes

Parameter	Definition	Value	Reference
$J_{cav/ecav}$	Rate of cAMP diffusion between caveolae and extracaveolae compartments	$5.000 \cdot 10^{-9}$ μL s^{-1}	Iancu et al. [14]
$J_{cav/cyt}$	Rate of cAMP diffusion between caveolae and cytosolic compartments	$7.500 \cdot 10^{-8}$ μL s^{-1}	Iancu et al. [14]
$J_{ecav/cyt}$	Rate of cAMP diffusion between extracaveolae and cytosolic compartments	$9.000 \cdot 10^{-9}$ μL s^{-1}	Iancu et al. [14]

$$\frac{d[cAMP]^{cav}}{dt} = \frac{d[cAMP]^{cav}_{PKA}}{dt} + \frac{d[cAMP]^{cav}_{AC56}}{dt} - \frac{d[cAMP]^{cav}_{PDE2}}{dt} - \frac{d[cAMP]^{cav}_{PDE3}}{dt} - \frac{d[cAMP]^{cav}_{PDE4}}{dt}$$
$$-J_{cav/ecav} \cdot \frac{[cAMP]^{cav} - [cAMP]^{ecav}}{V_{cav}} - J_{cav/cyt} \cdot \frac{[cAMP]^{cav} - [cAMP]^{cyt}}{V_{cav}} \quad \text{(A.131)}$$

$$\frac{d[cAMP]^{ecav}}{dt} = \frac{d[cAMP]^{ecav}_{PKA}}{dt} + \frac{d[cAMP]^{ecav}_{AC47}}{dt} - \frac{d[cAMP]^{ecav}_{PDE2}}{dt} - \frac{d[cAMP]^{ecav}_{PDE4}}{dt}$$
$$-J_{cav/ecav} \cdot \frac{[cAMP]^{ecav} - [cAMP]^{cav}}{V_{ecav}} - J_{ecav/cyt} \cdot \frac{[cAMP]^{ecav} - [cAMP]^{cyt}}{V_{ecav}} \quad \text{(A.132)}$$

$$\frac{d[cAMP]^{cyt}}{dt} = \frac{d[cAMP]^{cyt}_{PKA}}{dt} + \frac{d[cAMP]^{cyt}_{AC56}}{dt} + \frac{d[cAMP]^{cyt}_{AC47}}{dt} - \frac{d[cAMP]^{cyt}_{PDE2}}{dt} - \frac{d[cAMP]^{cyt}_{PDE3}}{dt} - \frac{d[cAMP]^{cyt}_{PDE4}}{dt}$$
$$-J_{cav/cyt} \cdot \frac{[cAMP]^{cyt} - [cAMP]^{cav}}{V_{cyt}} - J_{ecav/cyt} \cdot \frac{[cAMP]^{cyt} - [cAMP]^{ecav}}{V_{cyt}} \quad \text{(A.133)}$$

ELECTROPHYSIOLOGICAL PART. PKA SUBSTRATES

L-type Ca^{2+} current module

Parameter	Definition	Value	Reference
$[I_{CaL}]_{tot}$	Total cellular concentration of the L-type Ca^{2+} channels	0.0273 μM	Chu et al. [71]<br>Bers and Stiffel [72]
f_{ICaL}^{cav}	Fraction of the L-type Ca^{2+} channels located in caveolae	0.2	Scriven et al. [21]
f_{ICaL}^{ecav}	Fraction of the L-type Ca^{2+} channels located in extracaveolae	$1-f_{ICaL}^{cav}$	Scriven et al. [21]
G_{CaL}	Specific maximum conductivity for L-type Ca^{2+} channel (non-phosphorylated)	0.3772 mS/μF	This paper
G_{CaLp}	Specific maximum conductivity for L-type Ca^{2+} channel (phosphorylated)	0.7875 mS/μF	This paper
E_{CaL}	Reversal potential for L-type Ca^{2+} channel	52.0 mV	Petkova-Kirova et al. [26]
$K_{pc,\max}$	Maximum rate constant for Ca^{2+}-induced inactivation	233.24 s^{-1}	This paper
$K_{pc,half}$	Half-saturation constant for Ca^{2+}-induced inactivation	10.0 μM	This paper
K_{pcf}	Forward voltage-insensitive rate constant for inactivation	40,000 s^{-1}	This paper
K_{pcb}	Backward voltage-insensitive rate constant for inactivation	2.4 s^{-1}	This paper
k_{co}	Forward voltage-insensitive rate constant for activation (non-phosphorylated)	1,000 s^{-1}	This paper
k_{cop}	Forward voltage-insensitive rate constant for activation (phosphorylated)	4,000 s^{-1}	This paper
k_{oc}	Backward voltage-insensitive rate constant for activation	1,000 s^{-1}	This paper
$I_{CaL,\max}$	Normalization constant for L-type Ca^{2+} current	7.0 pA/pF	Bondarenko et al. [25]
k_{ICaL_PKA}	Phosphorylation rate of the L-type Ca^{2+} channel by PKA	$1.74 \cdot 10^{-2}$ s^{-1}	This paper
k_{ICaL_PP}	Dephosphorylation rate of the L-type Ca^{2+} channel by PP1 and PP2A	$2.325 \cdot 10^{-4}$ s^{-1}	This paper
K_{ICaL_PKA}	Affinity of the L-type Ca^{2+} channel for PKA	0.5 μM	This paper
K_{ICaL_PP}	Affinity of the L-type Ca^{2+} channel for PP1 and PP2A	0.2 μM	This paper

$$I_{CaL} = I_{ICaL}^{cav} + I_{ICaL}^{ecav} \quad \text{(A.134)}$$

$$\alpha = 0.4e^{(V+15.0)/15.0} \quad \text{(A.135)}$$

$$\alpha_p = 0.4e^{(V+15.0+20.0)/15.0} \quad \text{(A.136)}$$

$$\beta = 0.13e^{-(V+15.0)/18.0} \tag{A.137}$$

Caveolae

$$[I_{CaL}]_{tot}^{cav} = \mathrm{f}_{ICaL}^{cav}[I_{CaL}]_{tot}\frac{V_{cell}}{V_{cav}} \tag{A.138}$$

$$I_{CaL}^{cav} = \mathrm{f}_{ICaL}^{cav}\cdot(G_{CaL}\cdot O^{cav} + G_{CaLp}\cdot O_p^{cav})(V - E_{CaL}) \tag{A.139}$$

$$\gamma^{cav} = \frac{K_{pc,\max}[Ca^{2+}]_i}{K_{pc,half} + [Ca^{2+}]_i} \tag{A.140}$$

$$\begin{aligned}\frac{dO^{cav}}{dt} &= k_{co}C_P^{cav} - k_{oc}O^{cav} + K_{pcb}I_1^{cav} - \gamma^{cav}O^{cav} + 0.001(\alpha I_2^{cav} - K_{pcf}O^{cav})\\ &- \frac{k_{ICaL\_PKA}[C]^{cav}O^{cav}}{K_{ICaL\_PKA} + [I_{CaL}]_{tot}^{cav}O^{cav}} + \frac{\alpha}{\alpha_p}\frac{k_{ICaL\_PP}[PP]^{cav}O_p^{cav}}{K_{ICaL\_PP} + [I_{CaL}]_{tot}^{cav}O_p^{cav}}\end{aligned} \tag{A.141}$$

$$\begin{aligned}C_1^{cav} &= 1 - (O^{cav} + C_2^{cav} + C_3^{cav} + C_4^{cav} + C_P^{cav} + I_1^{cav} + I_2^{cav} + I_3^{cav}\\ &+ O_p^{cav} + C_{1p}^{cav} + C_{2p}^{cav} + C_{3p}^{cav} + C_{4p}^{cav} + C_{Pp}^{cav} + I_{1p}^{cav} + I_{2p}^{cav} + I_{3p}^{cav})\end{aligned} \tag{A.142}$$

$$\begin{aligned}\frac{dC_2^{cav}}{dt} &= 4\alpha C_1^{cav} - \beta C_2^{cav} + 2\beta C_3^{cav} - 3\alpha C_2^{cav}\\ &- \frac{k_{ICaL\_PKA}[C]^{cav}C_2^{cav}}{K_{ICaL\_PKA} + [I_{CaL}]_{tot}^{cav}C_2^{cav}} + \frac{\alpha_p^2 k_{cop}}{\alpha^2 k_{co}}\frac{k_{ICaL\_PP}[PP]^{cav}C_{2p}^{cav}}{K_{ICaL\_PP} + [I_{CaL}]_{tot}^{cav}C_{2p}^{cav}}\end{aligned} \tag{A.143}$$

$$\begin{aligned}\frac{dC_3^{cav}}{dt} &= 3\alpha C_2^{cav} - 2\beta C_3^{cav} + 3\beta C_4^{cav} - 2\alpha C_3^{cav}\\ &- \frac{k_{ICaL\_PKA}[C]^{cav}C_3^{cav}}{K_{ICaL\_PKA} + [I_{CaL}]_{tot}^{cav}C_3^{cav}} + \frac{\alpha_p k_{cop}}{\alpha k_{co}}\frac{k_{ICaL\_PP}[PP]^{cav}C_{3p}^{cav}}{K_{ICaL\_PP} + [I_{CaL}]_{tot}^{cav}C_{3p}^{cav}}\end{aligned} \tag{A.144}$$

$$\frac{dC_4^{cav}}{dt} = 2\alpha C_3^{cav} - 3\beta C_4^{cav} + 4\beta C_P^{cav} - \alpha C_4^{cav} + 0.01(4K_{pcb}\beta I_1^{cav} - \frac{k_{co}}{k_{oc}}\alpha\gamma^{cav}C_4^{cav})$$
$$+ 0.002(4\beta I_2^{cav} - \frac{k_{co}}{k_{oc}}K_{pcf}C_4^{cav}) + 4\beta K_{pcb}I_3^{cav} - \frac{k_{co}}{k_{oc}}\gamma^{cav}K_{pcf}C_4^{cav}$$
$$- \frac{k_{ICaL\_PKA}[C]^{cav}C_4^{cav}}{K_{ICaL\_PKA} + [I_{CaL}]_{tot}^{cav}C_4^{cav}} + \frac{k_{cop}}{k_{co}}\frac{k_{ICaL\_PP}[PP]^{cav}C_{4p}^{cav}}{K_{ICaL\_PP} + [I_{CaL}]_{tot}^{cav}C_{4p}^{cav}} \tag{A.145}$$

$$\frac{dC_P^{cav}}{dt} = \alpha C_4^{cav} - 4\beta C_P^{cav} + k_{oc}O^{cav} - k_{co}C_P^{cav}$$
$$- \frac{k_{ICaL\_PKA}[C]^{cav}C_P^{cav}}{K_{ICaL\_PKA} + [I_{CaL}]_{tot}^{cav}C_P^{cav}} + \frac{\alpha k_{cop}}{\alpha_p k_{co}}\frac{k_{ICaL\_PP}[PP]^{cav}C_{Pp}^{cav}}{K_{ICaL\_PP} + [I_{CaL}]_{tot}^{cav}C_{Pp}^{cav}} \tag{A.146}$$

$$\frac{dI_1^{cav}}{dt} = \gamma^{cav}O^{cav} - K_{pcb}I_1^{cav} + 0.001(\alpha I_3^{cav} - K_{pcf}I_1^{cav}) + 0.01(\frac{k_{co}}{k_{oc}}\alpha\gamma^{cav}C_4^{cav} - 4\beta K_{pcb}I_1^{cav})$$
$$- \frac{k_{ICaL\_PKA}[C]^{cav}I_1^{cav}}{K_{ICaL\_PKA} + [I_{CaL}]_{tot}^{cav}I_1^{cav}} + \frac{\alpha}{\alpha_p}\frac{k_{ICaL\_PP}[PP]^{cav}I_{1p}^{cav}}{K_{ICaL\_PP} + [I_{CaL}]_{tot}^{cav}I_{1p}^{cav}} \tag{A.147}$$

$$\frac{dI_2^{cav}}{dt} = 0.001(K_{pcf}O^{cav} - \alpha I_2^{cav}) + K_{pcb}I_3^{cav} - \gamma^{cav}I_2^{cav} + 0.002(\frac{k_{co}}{k_{oc}}K_{pcf}C_4^{cav} - 4\beta I_2^{cav})$$
$$- \frac{k_{ICaL\_PKA}[C]^{cav}I_2^{cav}}{K_{ICaL\_PKA} + [I_{CaL}]_{tot}^{cav}I_2^{cav}} + \frac{k_{ICaL\_PP}[PP]^{cav}I_{2p}^{cav}}{K_{ICaL\_PP} + [I_{CaL}]_{tot}^{cav}I_{2p}^{cav}} \tag{A.148}$$

$$\frac{dI_3^{cav}}{dt} = 0.001(K_{pcf}I_1^{cav} - \alpha I_3^{cav}) + \gamma^{cav}I_2^{cav} - K_{pcb}I_3^{cav} + \frac{k_{co}}{k_{oc}}\gamma^{cav}K_{pcf}C_4^{cav} - 4\beta K_{pcb}I_3^{cav}$$
$$- \frac{k_{ICaL\_PKA}[C]^{cav}I_3^{cav}}{K_{ICaL\_PKA} + [I_{CaL}]_{tot}^{cav}I_3^{cav}} + \frac{k_{ICaL\_PP}[PP]^{cav}I_{3p}^{cav}}{K_{ICaL\_PP} + [I_{CaL}]_{tot}^{cav}I_{3p}^{cav}} \tag{A.149}$$

$$\frac{dO_p^{cav}}{dt} = k_{cop}C_{Pp}^{cav} - k_{oc}O_p^{cav} + K_{pcb}I_{1p}^{cav} - \gamma^{cav}O_p^{cav} + 0.001(\alpha_p I_{2p}^{cav} - K_{pcf}O_p^{cav})$$
$$+ \frac{k_{ICaL\_PKA}[C]^{cav}O^{cav}}{K_{ICaL\_PKA} + [I_{CaL}]_{tot}^{cav}O^{cav}} - \frac{\alpha}{\alpha_p}\frac{k_{ICaL\_PP}[PP]^{cav}O_p^{cav}}{K_{ICaL\_PP} + [I_{CaL}]_{tot}^{cav}O_p^{cav}} \tag{A.150}$$

$$\frac{dC_{1p}^{cav}}{dt} = \beta C_{2p}^{cav} - 4\alpha_p C_{1p}^{cav} + \frac{k_{ICaL\_PKA}[C]^{cav}C_1^{cav}}{K_{ICaL\_PKA} + [I_{CaL}]_{tot}^{cav}C_1^{cav}} - \frac{\alpha_p^3 k_{cop}}{\alpha^3 k_{co}}\frac{k_{ICaL\_PP}[PP]^{cav}C_{1p}^{cav}}{K_{ICaL\_PP} + [I_{CaL}]_{tot}^{cav}C_{1p}^{cav}} \tag{A.151}$$

$$\frac{dC_{2p}^{cav}}{dt} = 4\alpha_p C_{1p}^{cav} - \beta C_{2p}^{cav} + 2\beta C_{3p}^{cav} - 3\alpha_p C_{2p}^{cav} + \frac{k_{ICaL\_PKA}[C]^{cav} C_2^{cav}}{K_{ICaL\_PKA} + [I_{CaL}]_{tot}^{cav} C_2^{cav}} - \frac{\alpha_p^2 k_{cop}}{\alpha^2 k_{co}} \frac{k_{ICaL\_PP}[PP]^{cav} C_{2p}^{cav}}{K_{ICaL\_PP} + [I_{CaL}]_{tot}^{cav} C_{2p}^{cav}} \quad (A.152)$$

$$\frac{dC_{3p}^{cav}}{dt} = 3\alpha_p C_{2p}^{cav} - 2\beta C_{3p}^{cav} + 3\beta C_{4p}^{cav} - 2\alpha_p C_{3p}^{cav} + \frac{k_{ICaL\_PKA}[C]^{cav} C_3^{cav}}{K_{ICaL\_PKA} + [I_{CaL}]_{tot}^{cav} C_3^{cav}} - \frac{\alpha_p k_{cop}}{\alpha k_{co}} \frac{k_{ICaL\_PP}[PP]^{cav} C_{3p}^{cav}}{K_{ICaL\_PP} + [I_{CaL}]_{tot}^{cav} C_{3p}^{cav}} \quad (A.153)$$

$$\frac{dC_{4p}^{cav}}{dt} = 2\alpha_p C_{3p}^{cav} - 3\beta C_{4p}^{cav} + 4\beta C_{Pp}^{cav} - \alpha_p C_{4p}^{cav} + 0.01(4K_{pcb}\beta I_{1p}^{cav} - \frac{k_{cop}}{k_{oc}}\alpha\gamma^{cav} C_{4p}^{cav}) + 0.002(4\beta I_{2p}^{cav} - \frac{k_{cop}}{k_{oc}} K_{pcf} C_{4p}^{cav}) + 4\beta K_{pcb} I_{3p}^{cav} - \frac{k_{cop}}{k_{oc}}\gamma^{cav} K_{pcf} C_{4p}^{cav} + \frac{k_{ICaL\_PKA}[C]^{cav} C_4^{cav}}{K_{ICaL\_PKA} + [I_{CaL}]_{tot}^{cav} C_4^{cav}} - \frac{k_{cop}}{k_{co}} \frac{k_{ICaL\_PP}[PP]^{cav} C_{4p}^{cav}}{K_{ICaL\_PP} + [I_{CaL}]_{tot}^{cav} C_{4p}^{cav}} \quad (A.154)$$

$$\frac{dC_{Pp}^{cav}}{dt} = \alpha_p C_{4p}^{cav} - 4\beta C_{Pp}^{cav} + k_{oc} O_p^{cav} - k_{cop} C_{Pp}^{cav} + \frac{k_{ICaL\_PKA}[C]^{cav} C_P^{cav}}{K_{ICaL\_PKA} + [I_{CaL}]_{tot}^{cav} C_P^{cav}} - \frac{\alpha k_{cop}}{\alpha_p k_{co}} \frac{k_{ICaL\_PP}[PP]^{cav} C_{Pp}^{cav}}{K_{ICaL\_PP} + [I_{CaL}]_{tot}^{cav} C_{Pp}^{cav}} \quad (A.155)$$

$$\frac{dI_{1p}^{cav}}{dt} = \gamma^{cav} O_p^{cav} - K_{pcb} I_{1p}^{cav} + 0.001(\alpha_p I_{3p}^{cav} - K_{pcf} I_{1p}^{cav}) + 0.01(\frac{k_{cop}}{k_{oc}}\alpha_p\gamma^{cav} C_{4p}^{cav} - 4\beta K_{pcb} I_{1p}^{cav}) + \frac{k_{ICaL\_PKA}[C]^{cav} I_1^{cav}}{K_{ICaL\_PKA} + [I_{CaL}]_{tot}^{cav} I_1^{cav}} - \frac{\alpha}{\alpha_p} \frac{k_{ICaL\_PP}[PP]^{cav} I_{1p}^{cav}}{K_{ICaL\_PP} + [I_{CaL}]_{tot}^{cav} I_{1p}^{cav}} \quad (A.156)$$

$$\frac{dI_{2p}^{cav}}{dt} = 0.001(K_{pcf} O_p^{cav} - \alpha_p I_{2p}^{cav}) + K_{pcb} I_{3p}^{cav} - \gamma^{cav} I_{2p}^{cav} + 0.002(\frac{k_{cop}}{k_{oc}} K_{pcf} C_{4p}^{cav} - 4\beta I_{2p}^{cav}) + \frac{k_{ICaL\_PKA}[C]^{cav} I_2^{cav}}{K_{ICaL\_PKA} + [I_{CaL}]_{tot}^{cav} I_2^{cav}} - \frac{k_{ICaL\_PP}[PP]^{cav} I_{2p}^{cav}}{K_{ICaL\_PP} + [I_{CaL}]_{tot}^{cav} I_{2p}^{cav}} \quad (A.157)$$

$$\frac{dI_{3p}^{cav}}{dt} = 0.001(K_{pcf}I_{1p}^{cav} - \alpha_p I_{3p}^{cav}) + \gamma^{cav} I_{2p}^{cav} - K_{pcb}I_{3p}^{cav} + \frac{k_{cop}}{k_{oc}}\gamma^{cav}K_{pcf}C_{4p}^{cav} - 4\beta K_{pcb}I_{3p}^{cav}$$
$$+ \frac{k_{ICaL\_PKA}[C]^{cav} I_3^{cav}}{K_{ICaL\_PKA} + [I_{CaL}]_{tot}^{cav} I_3^{cav}} - \frac{k_{ICaL\_PP}[PP]^{cav} I_{3p}^{cav}}{K_{ICaL\_PP} + [I_{CaL}]_{tot}^{cav} I_{3p}^{cav}} \quad \text{(A.158)}$$

Extracaveolae

$$[I_{CaL}]_{tot}^{ecav} = \mathrm{f}_{ICaL}^{ecav}[I_{CaL}]_{tot}\frac{V_{cell}}{V_{ecav}} \quad \text{(A.159)}$$

$$I_{CaL}^{ecav} = \mathrm{f}_{ICaL}^{ecav} \cdot (G_{CaL} \cdot O^{ecav} + G_{CaLp} \cdot O_p^{ecav})(V - E_{CaL}) \quad \text{(A.160)}$$

$$\gamma^{ecav} = \frac{K_{pc,\max}[Ca^{2+}]_{ss}}{K_{pc,half} + [Ca^{2+}]_{ss}} \quad \text{(A.161)}$$

$$\frac{dO^{ecav}}{dt} = k_{co}C_P^{ecav} - k_{oc}O^{ecav} + K_{pcb}I_1^{ecav} - \gamma^{ecav}O^{ecav} + 0.001(\alpha I_2^{ecav} - K_{pcf}O^{ecav})$$
$$- \frac{k_{ICaL\_PKA}[C]^{ecav} O^{ecav}}{K_{ICaL\_PKA} + [I_{CaL}]_{tot}^{ecav} O^{ecav}} + \frac{\alpha}{\alpha_p}\frac{k_{ICaL\_PP}[PP1]^{ecav} O_p^{ecav}}{K_{ICaL\_PP} + [I_{CaL}]_{tot}^{ecav} O_p^{ecav}} \quad \text{(A.162)}$$

$$C_1^{ecav} = 1 - (O^{ecav} + C_2^{ecav} + C_3^{ecav} + C_4^{ecav} + C_P^{ecav} + I_1^{ecav} + I_2^{ecav} + I_3^{ecav}$$
$$+ O_p^{ecav} + C_{1p}^{ecav} + C_{2p}^{ecav} + C_{3p}^{ecav} + C_{4p}^{ecav} + C_{Pp}^{ecav} + I_{1p}^{ecav} + I_{2p}^{ecav} + I_{3p}^{ecav}) \quad \text{(A.163)}$$

$$\frac{dC_2^{ecav}}{dt} = 4\alpha C_1^{ecav} - \beta C_2^{ecav} + 2\beta C_3^{ecav} - 3\alpha C_2^{ecav}$$
$$- \frac{k_{ICaL\_PKA}[C]^{ecav} C_2^{ecav}}{K_{ICaL\_PKA} + [I_{CaL}]_{tot}^{ecav} C_2^{ecav}} + \frac{\alpha_p^2 k_{cop}}{\alpha^2 k_{co}}\frac{k_{ICaL\_PP}[PP1]^{ecav} C_{2p}^{ecav}}{K_{ICaL\_PP} + [I_{CaL}]_{tot}^{ecav} C_{2p}^{ecav}} \quad \text{(A.164)}$$

$$\frac{dC_3^{ecav}}{dt} = 3\alpha C_2^{ecav} - 2\beta C_3^{ecav} + 3\beta C_4^{ecav} - 2\alpha C_3^{ecav}$$
$$- \frac{k_{ICaL\_PKA}[C]^{ecav} C_3^{ecav}}{K_{ICaL\_PKA} + [I_{CaL}]_{tot}^{ecav} C_3^{ecav}} + \frac{\alpha_p k_{cop}}{\alpha k_{co}}\frac{k_{ICaL\_PP}[PP1]^{ecav} C_{3p}^{ecav}}{K_{ICaL\_PP} + [I_{CaL}]_{tot}^{ecav} C_{3p}^{ecav}} \quad \text{(A.165)}$$

$$\frac{dC_4^{ecav}}{dt} = 2\alpha C_3^{ecav} - 3\beta C_4^{ecav} + 4\beta C_P^{ecav} - \alpha C_4^{ecav} + 0.01(4K_{pcb}\beta I_1^{ecav} - \frac{k_{co}}{k_{oc}}\alpha\gamma^{ecav}C_4^{ecav}) + 0.002(4\beta I_2^{ecav} - \frac{k_{co}}{k_{oc}}K_{pcf}C_4^{ecav}) + 4\beta K_{pcb}I_3^{ecav} - \frac{k_{co}}{k_{oc}}\gamma^{ecav}K_{pcf}C_4^{ecav} - \frac{k_{ICaL\_PKA}[C]^{ecav}C_4^{ecav}}{K_{ICaL\_PKA} + [I_{CaL}]_{tot}^{ecav}C_4^{ecav}} + \frac{k_{cop}}{k_{co}}\frac{k_{ICaL\_PP}[PP1]^{ecav}C_{4p}^{ecav}}{K_{ICaL\_PP} + [I_{CaL}]_{tot}^{ecav}C_{4p}^{ecav}} \quad \text{(A.166)}$$

$$\frac{dC_P^{ecav}}{dt} = \alpha C_4^{ecav} - 4\beta C_P^{ecav} + k_{oc}O^{ecav} - k_{co}C_P^{ecav} - \frac{k_{ICaL\_PKA}[C]^{ecav}C_P^{ecav}}{K_{ICaL\_PKA} + [I_{CaL}]_{tot}^{ecav}C_P^{ecav}} + \frac{\alpha k_{cop}}{\alpha_p k_{co}}\frac{k_{ICaL\_PP}[PP1]^{ecav}C_{Pp}^{ecav}}{K_{ICaL\_PP} + [I_{CaL}]_{tot}^{ecav}C_{Pp}^{ecav}} \quad \text{(A.167)}$$

$$\frac{dI_1^{ecav}}{dt} = \gamma^{ecav}O^{ecav} - K_{pcb}I_1^{ecav} + 0.001(\alpha I_3^{ecav} - K_{pcf}I_1^{ecav}) + 0.01(\frac{k_{co}}{k_{oc}}\alpha\gamma^{ecav}C_4^{ecav} - 4\beta K_{pcb}I_1^{ecav}) - \frac{k_{ICaL\_PKA}[C]^{ecav}I_1^{ecav}}{K_{ICaL\_PKA} + [I_{CaL}]_{tot}^{ecav}I_1^{ecav}} + \frac{\alpha}{\alpha_p}\frac{k_{ICaL\_PP}[PP1]^{ecav}I_{1p}^{ecav}}{K_{ICaL\_PP} + [I_{CaL}]_{tot}^{ecav}I_{1p}^{ecav}} \quad \text{(A.168)}$$

$$\frac{dI_2^{ecav}}{dt} = 0.001(K_{pcf}O^{ecav} - \alpha I_2^{ecav}) + K_{pcb}I_3^{ecav} - \gamma^{ecav}I_2^{ecav} + 0.002(\frac{k_{co}}{k_{oc}}K_{pcf}C_4^{ecav} - 4\beta I_2^{ecav}) - \frac{k_{ICaL\_PKA}[C]^{ecav}I_2^{ecav}}{K_{ICaL\_PKA} + [I_{CaL}]_{tot}^{ecav}I_2^{ecav}} + \frac{k_{ICaL\_PP}[PP1]^{ecav}I_{2p}^{ecav}}{K_{ICaL\_PP} + [I_{CaL}]_{tot}^{ecav}I_{2p}^{ecav}} \quad \text{(A.169)}$$

$$\frac{dI_3^{ecav}}{dt} = 0.001(K_{pcf}I_1^{ecav} - \alpha I_3^{ecav}) + \gamma^{ecav}I_2^{ecav} - K_{pcb}I_3^{ecav} + \frac{k_{co}}{k_{oc}}\gamma^{ecav}K_{pcf}C_4^{ecav} - 4\beta K_{pcb}I_3^{ecav} - \frac{k_{ICaL\_PKA}[C]^{ecav}I_3^{ecav}}{K_{ICaL\_PKA} + [I_{CaL}]_{tot}^{ecav}I_3^{ecav}} + \frac{k_{ICaL\_PP}[PP1]^{ecav}I_{3p}^{ecav}}{K_{ICaL\_PP} + [I_{CaL}]_{tot}^{ecav}I_{3p}^{ecav}} \quad \text{(A.170)}$$

$$\frac{dO_p^{ecav}}{dt} = k_{cop}C_{Pp}^{ecav} - k_{oc}O_p^{ecav} + K_{pcb}I_{1p}^{ecav} - \gamma^{ecav}O_p^{ecav} + 0.001(\alpha_p I_{2p}^{ecav} - K_{pcf}O_p^{ecav}) + \frac{k_{ICaL\_PKA}[C]^{ecav}O^{ecav}}{K_{ICaL\_PKA} + [I_{CaL}]_{tot}^{ecav}O^{ecav}} - \frac{\alpha}{\alpha_p}\frac{k_{ICaL\_PP}[PP1]^{ecav}O_p^{ecav}}{K_{ICaL\_PP} + [I_{CaL}]_{tot}^{ecav}O_p^{ecav}} \quad \text{(A.171)}$$

$$\frac{dC_{1p}^{ecav}}{dt} = \beta C_{2p}^{ecav} - 4\alpha_p C_{1p}^{ecav} + \frac{k_{ICaL\_PKA}[C]^{ecav}C_1^{ecav}}{K_{ICaL\_PKA} + [I_{CaL}]_{tot}^{ecav}C_1^{ecav}} - \frac{\alpha_p^3 k_{cop}}{\alpha^3 k_{co}}\frac{k_{ICaL\_PP}[PP1]^{ecav}C_{1p}^{ecav}}{K_{ICaL\_PP} + [I_{CaL}]_{tot}^{ecav}C_{1p}^{ecav}} \quad \text{(A.172)}$$

$$\frac{dC_{2p}^{ecav}}{dt} = 4\alpha_p C_{1p}^{ecav} - \beta C_{2p}^{ecav} + 2\beta C_{3p}^{ecav} - 3\alpha_p C_{2p}^{ecav} + \frac{k_{ICaL\_PKA}[C]^{ecav} C_2^{ecav}}{K_{ICaL\_PKA} + [I_{CaL}]_{tot}^{ecav} C_2^{ecav}} - \frac{\alpha_p^2 k_{cop}}{\alpha^2 k_{co}} \frac{k_{ICaL\_PP}[PP1]^{ecav} C_{2p}^{ecav}}{K_{ICaL\_PP} + [I_{CaL}]_{tot}^{ecav} C_{2p}^{ecav}} \quad (A.173)$$

$$\frac{dC_{3p}^{ecav}}{dt} = 3\alpha_p C_{2p}^{ecav} - 2\beta C_{3p}^{ecav} + 3\beta C_{4p}^{ecav} - 2\alpha_p C_{3p}^{ecav} + \frac{k_{ICaL\_PKA}[C]^{ecav} C_3^{ecav}}{K_{ICaL\_PKA} + [I_{CaL}]_{tot}^{ecav} C_3^{ecav}} - \frac{\alpha_p k_{cop}}{\alpha k_{co}} \frac{k_{ICaL\_PP}[PP1]^{ecav} C_{3p}^{ecav}}{K_{ICaL\_PP} + [I_{CaL}]_{tot}^{ecav} C_{3p}^{ecav}} \quad (A.174)$$

$$\frac{dC_{4p}^{ecav}}{dt} = 2\alpha_p C_{3p}^{ecav} - 3\beta C_{4p}^{ecav} + 4\beta C_{Pp}^{ecav} - \alpha_p C_{4p}^{ecav} + 0.01(4K_{pcb}\beta I_{1p}^{ecav} - \frac{k_{cop}}{k_{oc}} \alpha \gamma^{ecav} C_{4p}^{ecav}) + 0.002(4\beta I_{2p}^{ecav} - \frac{k_{cop}}{k_{oc}} K_{pcf} C_{4p}^{ecav}) + 4\beta K_{pcb} I_{3p}^{ecav} - \frac{k_{cop}}{k_{oc}} \gamma^{ecav} K_{pcf} C_{4p}^{ecav} + \frac{k_{ICaL\_PKA}[C]^{ecav} C_4^{ecav}}{K_{ICaL\_PKA} + [I_{CaL}]_{tot}^{ecav} C_4^{ecav}} - \frac{k_{cop}}{k_{co}} \frac{k_{ICaL\_PP}[PP1]^{ecav} C_{4p}^{ecav}}{K_{ICaL\_PP} + [I_{CaL}]_{tot}^{ecav} C_{4p}^{ecav}} \quad (A.175)$$

$$\frac{dC_{Pp}^{ecav}}{dt} = \alpha_p C_{4p}^{ecav} - 4\beta C_{Pp}^{ecav} + k_{oc} O_p^{ecav} - k_{cop} C_{Pp}^{ecav} + \frac{k_{ICaL\_PKA}[C]^{ecav} C_P^{ecav}}{K_{ICaL\_PKA} + [I_{CaL}]_{tot}^{ecav} C_P^{ecav}} - \frac{\alpha k_{cop}}{\alpha_p k_{co}} \frac{k_{ICaL\_PP}[PP1]^{ecav} C_{Pp}^{ecav}}{K_{ICaL\_PP} + [I_{CaL}]_{tot}^{ecav} C_{Pp}^{ecav}} \quad (A.176)$$

$$\frac{dI_{1p}^{ecav}}{dt} = \gamma^{ecav} O_p^{ecav} - K_{pcb} I_{1p}^{ecav} + 0.001(\alpha_p I_{3p}^{ecav} - K_{pcf} I_{1p}^{ecav}) + 0.01(\frac{k_{cop}}{k_{oc}} \alpha_p \gamma^{ecav} C_{4p}^{ecav} - 4\beta K_{pcb} I_{1p}^{ecav}) + \frac{k_{ICaL\_PKA}[C]^{ecav} I_1^{ecav}}{K_{ICaL\_PKA} + [I_{CaL}]_{tot}^{ecav} I_1^{ecav}} - \frac{\alpha}{\alpha_p} \frac{k_{ICaL\_PP}[PP1]^{ecav} I_{1p}^{ecav}}{K_{ICaL\_PP} + [I_{CaL}]_{tot}^{ecav} I_{1p}^{ecav}} \quad (A.177)$$

$$\frac{dI_{2p}^{ecav}}{dt} = 0.001(K_{pcf} O_p^{ecav} - \alpha_p I_{2p}^{ecav}) + K_{pcb} I_{3p}^{ecav} - \gamma^{ecav} I_{2p}^{ecav} + 0.002(\frac{k_{cop}}{k_{oc}} K_{pcf} C_{4p}^{ecav} - 4\beta I_{2p}^{ecav}) + \frac{k_{ICaL\_PKA}[C]^{ecav} I_2^{ecav}}{K_{ICaL\_PKA} + [I_{CaL}]_{tot}^{ecav} I_2^{ecav}} - \frac{k_{ICaL\_PP}[PP1]^{ecav} I_{2p}^{ecav}}{K_{ICaL\_PP} + [I_{CaL}]_{tot}^{ecav} I_{2p}^{ecav}} \quad (A.178)$$

$$\frac{dI_{3p}^{ecav}}{dt} = 0.001(K_{pcf} I_{1p}^{ecav} - \alpha_p I_{3p}^{ecav}) + \gamma^{ecav} I_{2p}^{ecav} - K_{pcb} I_{3p}^{ecav} + \frac{k_{cop}}{k_{oc}} \gamma^{ecav} K_{pcf} C_{4p}^{ecav} - 4\beta K_{pcb} I_{3p}^{ecav}$$
$$+ \frac{k_{ICaL\_PKA}[C]^{ecav} I_3^{ecav}}{K_{ICaL\_PKA} + [I_{CaL}]_{tot}^{ecav} I_3^{ecav}} - \frac{k_{ICaL\_PP}[PP1]^{ecav} I_{3p}^{ecav}}{K_{ICaL\_PP} + [I_{CaL}]_{tot}^{ecav} I_{3p}^{ecav}} \quad \text{(A.179)}$$

Fast Na+ current module

Parameter	Definition	Value	Reference
G_{Na}	Specific maximum conductivity for the fast Na^+ channel (non-phosphorylated)	14.4 mS/μF	This paper
G_{Nap}	Specific maximum conductivity for the fast Na^+ channel (phosphorylated)	18.0 mS/μF	This paper
k_{INa_PKA}	Phosphorylation-traffiking rate of the fast Na^+ channel by PKA	$6.8400 \cdot 10^{-3}$ μM^{-1} s^{-1}	This paper
k_{INa_PP}	Dephosphorylation rate of the fast Na^+ channel by PP1 and PP2A	$1.9804 \cdot 10^{-2}$ μM^{-1} s^{-1}	This paper
K_{INa_PKA}	Affinity of the fast Na^+ channel for PKA	$5.49415 \cdot 10^{-3}$	This paper
K_{INa_PP}	Affinity of the fast Na^+ channel for PP1 and PP2A	0.393025	This paper

$$E_{Na} = \frac{RT}{F} \ln\left(\frac{0.9[Na^+]_o + 0.1[K^+]_o}{0.9[Na^+]_i + 0.1[K^+]_i} \right) \quad \text{(A.180)}$$

$$I_{Na} = (G_{Na} \cdot O_{Na} + G_{Nap} \cdot O_{Nap})(V - E_{Na}) \quad \text{(A.181)}$$

$$C_{Na3} = 1 - (O_{Na} + C_{Na2} + C_{Na1} + IF_{Na} + I1_{Na} + I2_{Na} + IC_{Na2} + IC_{Na3}$$
$$+ O_{Nap} + C_{Na3p} + C_{Na2p} + C_{Na1p} + IF_{Nap} + I1_{Nap} + I2_{Nap} + IC_{Na2p} + IC_{Na3p}) \quad \text{(A.182)}$$

$$\frac{dC_{Na2}}{dt} = \alpha_{Na11} C_{Na3} - \beta_{Na11} C_{Na2} + \beta_{Na12} C_{Na1} - \alpha_{Na12} C_{Na2} + \alpha_{Na3} IC_{Na2} - \beta_{Na3} C_{Na2}$$
$$- \frac{k_{INa\_PKA}[C]^{cav} C_{Na2}}{K_{INa\_PKA} + C_{Na2}} + \frac{k_{INa\_PP}[PP]^{cav} C_{Na2p}}{K_{INa\_PP} + C_{Na2p}} \quad \text{(A.183)}$$

$$\frac{dC_{Na1}}{dt} = \alpha_{Na12}C_{Na2} - \beta_{Na12}C_{Na1} + \beta_{Na13}O_{Na} - \alpha_{Na13}C_{Na1} + \alpha_{Na3}IF_{Na} - \beta_{Na3}C_{Na1} - \frac{k_{INa\_PKA}[C]^{cav}C_{Na1}}{K_{INa\_PKA} + C_{Na1}} + \frac{k_{INa\_PP}[PP]^{cav}C_{Na1p}}{K_{INa\_PP} + C_{Na1p}} \tag{A.184}$$

$$\frac{dO_{Na}}{dt} = \alpha_{Na13}C_{Na1} - \beta_{Na13}O_{Na} + \beta_{Na2}IF_{Na} - \alpha_{Na2}O_{Na} - \frac{k_{INa\_PKA}[C]^{cav}O_{Na}}{K_{INa\_PKA} + O_{Na}} + \frac{k_{INa\_PP}[PP]^{cav}O_{Nap}}{K_{INa\_PP} + O_{Nap}} \tag{A.185}$$

$$\frac{dIF_{Na}}{dt} = \alpha_{Na2}O_{Na} - \beta_{Na2}IF_{Na} + \beta_{Na3}C_{Na1} - \alpha_{Na3}IF_{Na} + \beta_{Na4}I1_{Na} - \alpha_{Na4}IF_{Na} + \alpha_{Na12}IC_{Na2} - \beta_{Na12}IF_{Na} - \frac{k_{INa\_PKA}[C]^{cav}IF_{Na}}{K_{INa\_PKA} + IF_{Na}} + \frac{k_{INa\_PP}[PP]^{cav}IF_{Nap}}{K_{INa\_PP} + IF_{Nap}} \tag{A.186}$$

$$\frac{dI1_{Na}}{dt} = \alpha_{Na4}IF_{Na} - \beta_{Na4}I1_{Na} + \beta_{Na5}I2_{Na} - \alpha_{Na5}I1_{Na} - \frac{k_{INa\_PKA}[C]^{cav}I1_{Na}}{K_{INa\_PKA} + I1_{Na}} + \frac{k_{INa\_PP}[PP]^{cav}I1_{Nap}}{K_{INa\_PP} + I1_{Nap}} \tag{A.187}$$

$$\frac{dI2_{Na}}{dt} = \alpha_{Na5}I1_{Na} - \beta_{Na5}I2_{Na} - \frac{k_{INa\_PKA}[C]^{cav}I2_{Na}}{K_{INa\_PKA} + I2_{Na}} + \frac{k_{INa\_PP}[PP]^{cav}I2_{Nap}}{K_{INa\_PP} + I2_{Nap}} \tag{A.188}$$

$$\frac{dIC_{Na2}}{dt} = \alpha_{Na11}IC_{Na3} - \beta_{Na11}IC_{Na2} + \beta_{Na12}IF_{Na} - \alpha_{Na12}IC_{Na2} + \beta_{Na3}C_{Na2} - \alpha_{Na3}IC_{Na2} - \frac{k_{INa\_PKA}[C]^{cav}IC_{Na2}}{K_{INa\_PKA} + IC_{Na2}} + \frac{k_{INa\_PP}[PP]^{cav}IC_{Na2p}}{K_{INa\_PP} + IC_{Na2p}} \tag{A.189}$$

$$\frac{dIC_{Na3}}{dt} = \beta_{Na11}IC_{Na2} - \alpha_{Na11}IC_{Na3} + \beta_{Na3}C_{Na3} - \alpha_{Na3}IC_{Na3} - \frac{k_{INa\_PKA}[C]^{cav}IC_{Na3}}{K_{INa\_PKA} + IC_{Na3}} + \frac{k_{INa\_PP}[PP]^{cav}IC_{Na3p}}{K_{INa\_PP} + IC_{Na3p}} \tag{A.190}$$

$$\frac{dC_{Na3p}}{dt} = \beta_{Na11}C_{Na2p} - \alpha_{Na11}C_{Na3p} + \alpha_{Na3}IC_{Na3p} - \beta_{Na3}C_{Na3p} + \frac{k_{INa\_PKA}[C]^{cav}C_{Na3}}{K_{INa\_PKA} + C_{Na3}} - \frac{k_{INa\_PP}[PP]^{cav}C_{Na3p}}{K_{INa\_PP} + C_{Na3p}} \tag{A.191}$$

$$\frac{dC_{Na2p}}{dt} = \alpha_{Na11}C_{Na3p} - \beta_{Na11}C_{Na2p} + \beta_{Na12}C_{Na1p} - \alpha_{Na12}C_{Na2p} + \alpha_{Na3}IC_{Na2p} - \beta_{Na3}C_{Na2p} + \frac{k_{INa\_PKA}[C]^{cav}C_{Na2}}{K_{INa\_PKA} + C_{Na2}} - \frac{k_{INa\_PP}[PP]^{cav}C_{Na2p}}{K_{INa\_PP} + C_{Na2p}} \tag{A.192}$$

$$\frac{dC_{Na1p}}{dt} = \alpha_{Na12}C_{Na2p} - \beta_{Na12}C_{Na1p} + \beta_{Na13}O_{Nap} - \alpha_{Na13}C_{Na1p} + \alpha_{Na3}IF_{Nap} - \beta_{Na3}C_{Na1p} + \frac{k_{INa\_PKA}[C]^{cav}C_{Na1}}{K_{INa\_PKA} + C_{Na1}} - \frac{k_{INa\_PP}[PP]^{cav}C_{Na1p}}{K_{INa\_PP} + C_{Na1p}} \tag{A.193}$$

$$\frac{dO_{Nap}}{dt} = \alpha_{Na13}C_{Na1p} - \beta_{Na13}O_{Nap} + \beta_{Na2}IF_{Nap} - \alpha_{Na2}O_{Nap} + \frac{k_{INa\_PKA}[C]^{cav}O_{Na}}{K_{INa\_PKA} + O_{Na}} - \frac{k_{INa\_PP}[PP]^{cav}O_{Nap}}{K_{INa\_PP} + O_{Nap}} \tag{A.194}$$

$$\frac{dIF_{Nap}}{dt} = \alpha_{Na2}O_{Nap} - \beta_{Na2}IF_{Nap} + \beta_{Na3}C_{Na1p} - \alpha_{Na3}IF_{Nap} + \beta_{Na4}I1_{Nap} - \alpha_{Na4}IF_{Nap} + \alpha_{Na12}IC_{Na2p} - \beta_{Na12}IF_{Nap} + \frac{k_{INa\_PKA}[C]^{cav}IF_{Na}}{K_{INa\_PKA} + IF_{Na}} - \frac{k_{INa\_PP}[PP]^{cav}IF_{Nap}}{K_{INa\_PP} + IF_{Nap}} \tag{A.195}$$

$$\frac{dI1_{Nap}}{dt} = \alpha_{Na4}IF_{Nap} - \beta_{Na4}I1_{Nap} + \beta_{Na5}I2_{Nap} - \alpha_{Na5}I1_{Nap} + \frac{k_{INa\_PKA}[C]^{cav}I1_{Na}}{K_{INa\_PKA} + I1_{Na}} - \frac{k_{INa\_PP}[PP]^{cav}I1_{Nap}}{K_{INa\_PP} + I1_{Nap}} \tag{A.196}$$

$$\frac{dI2_{Nap}}{dt} = \alpha_{Na5}I1_{Nap} - \beta_{Na5}I2_{Nap} + \frac{k_{INa\_PKA}[C]^{cav}I2_{Na}}{K_{INa\_PKA} + I2_{Na}} - \frac{k_{INa\_PP}[PP]^{cav}I2_{Nap}}{K_{INa\_PP} + I2_{Nap}} \tag{A.197}$$

$$\frac{dIC_{Na2p}}{dt} = \alpha_{Na11} IC_{Na3p} - \beta_{Na11} IC_{Na2p} + \beta_{Na12} IF_{Nap} - \alpha_{Na12} IC_{Na2p} + \beta_{Na3} C_{Na2p} - \alpha_{Na3} IC_{Na2p}$$
$$+ \frac{k_{INa\_PKA}[C]^{cav} IC_{Na2}}{K_{INa\_PKA} + IC_{Na2}} - \frac{k_{INa\_PP}[PP]^{cav} IC_{Na2p}}{K_{INa\_PP} + IC_{Na2p}} \tag{A.198}$$

$$\frac{dIC_{Na3p}}{dt} = \beta_{Na11} IC_{Na2p} - \alpha_{Na11} IC_{Na3p} + \beta_{Na3} C_{Na3p} - \alpha_{Na3} IC_{Na3p}$$
$$+ \frac{k_{INa\_PKA}[C]^{cav} IC_{Na3}}{K_{INa\_PKA} + IC_{Na3}} - \frac{k_{INa\_PP}[PP]^{cav} IC_{Na3p}}{K_{INa\_PP} + IC_{Na3p}} \tag{A.199}$$

$$\alpha_{Na11} = \frac{3.802}{0.1027 e^{-(V-2.5)/17.0} + 0.20 e^{-(V-2.5)/150.0}} \tag{A.200}$$

$$\alpha_{Na12} = \frac{3.802}{0.1027 e^{-(V-2.5)/15.0} + 0.23 e^{-(V-2.5)/150.0}} \tag{A.201}$$

$$\alpha_{Na13} = \frac{3.802}{0.1027 e^{-(V-2.5)/12.0} + 0.25 e^{-(V-2.5)/150.0}} \tag{A.202}$$

$$\beta_{Na11} = 0.1917 e^{-(V-2.5)/20.3} \tag{A.203}$$

$$\beta_{Na12} = 0.20 e^{-(V-7.5)/20.3} \tag{A.204}$$

$$\beta_{Na13} = 0.22 e^{-(V-12.5)/20.3} \tag{A.205}$$

$$\alpha_{Na3} = 7.0 \times 10^{-7} e^{-(V+7.0)/7.7} \tag{A.206}$$

$$\beta_{Na3} = 0.0084 + 0.00002(V + 7.0) \tag{A.207}$$

$$\alpha_{Na2} = \frac{1.0}{0.188495 e^{-(V+7.0)/16.6} + 0.393956} \tag{A.208}$$

$$\beta_{Na2} = \alpha_{Na13} \alpha_{Na2} \alpha_{Na3} / (\beta_{Na13} \beta_{Na3}) \tag{A.209}$$

$$\alpha_{Na4} = \alpha_{Na2} / 100.0 \tag{A.210}$$

$$\beta_{Na4} = \alpha_{Na3} \tag{A.211}$$

$$\alpha_{Na5} = \alpha_{Na2} / 95000 \tag{A.212}$$

$$\beta_{Na5} = \alpha_{Na3} / 50.0 \tag{A.213}$$

Ryanodine receptor module

Parameter	Definition	Value	Reference
$[RyR]_{tot}$	Total cellular concentration of ryanodine receptors	0.1993 μM	Chu et al. [71]
v_1	Maximum RyR channel Ca^{2+} permeability	4,500 s^{-1}	Bondarenko et al. [25]
n	RyR Ca^{2+} cooperativity parameter $P_{C1} – P_{O1}$	4	Bondarenko et al. [25]
m	RyR Ca^{2+} cooperativity parameter $P_{O1} – P_{O2}$	3	Bondarenko et al. [25]
k_a^+	RyR $P_{C1} – P_{O1}$ rate constant	6.075 μM^{-4} s^{-1}	Bondarenko et al. [25]
k_a^-	RyR $P_{O1} – P_{C1}$ rate constant	71.25 s^{-1}	Bondarenko et al. [25]
k_b^+	RyR $P_{O1} – P_{O2}$ rate constant	4.05 μM^{-3} s^{-1}	Bondarenko et al. [25]
k_b^-	RyR $P_{O2} – P_{O1}$ rate constant	965.0 s^{-1}	Bondarenko et al. [25]
k_c^+	RyR $P_{O1} – P_{C2}$ rate constant	9.0 s^{-1}	Bondarenko et al. [25]
k_c^-	RyR $P_{C2} – P_{O1}$ rate constant	0.8 s^{-1}	Bondarenko et al. [25]
k_{ap}^+	RyR $P_{C1p} – P_{O1p}$ rate constant	$5k_a^+$	This paper
k_{ap}^-	RyR $P_{O1p} – P_{C1p}$ rate constant	$3k_a^-$	This paper
k_{bp}^+	RyR $P_{O1p} – P_{O2p}$ rate constant	$5k_b^+$	This paper
k_{bp}^-	RyR $P_{O2p} – P_{O1p}$ rate constant	$3k_b^-$	This paper
k_{cp}^+	RyR $P_{O1p} – P_{C2p}$ rate constant	$50k_c^+$	This paper
k_{cp}^-	RyR $P_{C2p} – P_{O1p}$ rate constant	$30k_c^-$	This paper
f_{RyR}	Allosteric factor for RyR	0.001	This paper
k_{RyR_PKA}	Phosphorylation rate of ryanodine receptors by PKA	$5.775 \cdot 10^{-2}$ μM^{-1} s^{-1}	This paper

k_{RyR_PP}	Dephosphorylation rate of ryanodine receptors by PP1	0.28875 μM^{-1} s^{-1}	This paper
K_{RyR_PKA}	Affinity of ryanodine receptors for PKA	0.5 μM	This paper
K_{RyR_PP}	Affinity of ryanodine receptors for PP1	0.05 μM	This paper

$$[RyR]^{ecav} = [RyR]_{tot} \frac{V_{cell}}{V_{ecav}} \quad \text{(A.214)}$$

$$P_{C1} = 1 - (P_{C2} + P_{O1} + P_{O2} + P_{C1p} + P_{C2p} + P_{O1p} + P_{O2p}) \quad \text{(A.215)}$$

$$\begin{aligned}\frac{dP_{O1}}{dt} &= k_a^+ [Ca^{2+}]_{ss}^n P_{C1} - k_a^- P_{O1} - k_b^+ [Ca^{2+}]_{ss}^m P_{O1} + k_b^- P_{O2} - k_c^+ P_{O1} + k_c^- P_{C2} \\ &- \frac{f_{RyR} k_{RyR\_PKA} [C]^{ecav} P_{O1}}{K_{RyR\_PKA} + [RyR]^{ecav} P_{O1}} + \frac{k_a^+ k_{ap}^-}{k_{ap}^+ k_a^-} \frac{f_{RyR} k_{RyR\_PP} [PP1]^{ecav} P_{O1p}}{K_{RyR\_PP} + [RyR]^{ecav} P_{O1p}}\end{aligned} \quad \text{(A.216)}$$

$$\begin{aligned}\frac{dP_{O2}}{dt} &= k_b^+ [Ca^{2+}]_{ss}^m P_{O1} - k_b^- P_{O2} \\ &- \frac{f_{RyR} k_{RyR\_PKA} [C]^{ecav} P_{O2}}{K_{RyR\_PKA} + [RyR]^{ecav} P_{O2}} + \frac{k_a^+ k_{ap}^- k_b^+ k_{bp}^-}{k_{ap}^+ k_a^- k_{bp}^+ k_b^-} \frac{f_{RyR} k_{RyR\_PP} [PP1]^{ecav} P_{O2p}}{K_{RyR\_PP} + [RyR]^{ecav} P_{O2p}}\end{aligned} \quad \text{(A.217)}$$

$$\begin{aligned}\frac{dP_{C2}}{dt} &= k_c^+ P_{O1} - k_c^- P_{C2} \\ &- \frac{f_{RyR} k_{RyR\_PKA} [C]^{ecav} P_{C2}}{K_{RyR\_PKA} + [RyR]^{ecav} P_{C2}} + \frac{k_a^+ k_{ap}^- k_c^+ k_{cp}^-}{k_{ap}^+ k_a^- k_{cp}^+ k_c^-} \frac{f_{RyR} k_{RyR\_PP} [PP1]^{ecav} P_{C2p}}{K_{RyR\_PP} + [RyR]^{ecav} P_{C2p}}\end{aligned} \quad \text{(A.218)}$$

$$\begin{aligned}\frac{dP_{C1p}}{dt} &= -k_{ap}^+ [Ca^{2+}]_{ss}^n P_{C1p} + k_{ap}^- P_{O1p} \\ &+ \frac{k_{RyR\_PKA} [C]^{ecav} P_{C1}}{K_{RyR\_PKA} + [RyR]^{ecav} P_{C1}} - \frac{k_{RyR\_PP} [PP1]^{ecav} P_{C1p}}{K_{RyR\_PP} + [RyR]^{ecav} P_{C1p}}\end{aligned} \quad \text{(A.219)}$$

$$\begin{aligned}\frac{dP_{O1p}}{dt} &= k_{ap}^+ [Ca^{2+}]_{ss}^n P_{C1p} - k_{ap}^- P_{O1p} - k_{bp}^+ [Ca^{2+}]_{ss}^m P_{O1p} + k_{bp}^- P_{O2p} - k_{cp}^+ P_{O1p} + k_{cp}^- P_{C2p} \\ &+ \frac{f_{RyR} k_{RyR\_PKA} [C]^{ecav} P_{O1}}{K_{RyR\_PKA} + [RyR]^{ecav} P_{O1}} - \frac{k_a^+ k_{ap}^-}{k_{ap}^+ k_a^-} \frac{f_{RyR} k_{RyR\_PP} [PP1]^{ecav} P_{O1p}}{K_{RyR\_PP} + [RyR]^{ecav} P_{O1p}}\end{aligned} \quad \text{(A.220)}$$

$$\frac{dP_{O2p}}{dt} = k_{bp}^{+}[Ca^{2+}]_{ss}^{m} P_{O1p} - k_{bp}^{-} P_{O2p} + \frac{f_{RyR} k_{RyR\_PKA} [C]^{ecav} P_{O2}}{K_{RyR\_PKA} + [RyR]^{ecav} P_{O2}} - \frac{k_a^{+} k_{ap}^{-} k_b^{+} k_{bp}^{-}}{k_{ap}^{+} k_a^{-} k_{bp}^{+} k_b^{-}} \frac{f_{RyR} k_{RyR\_PP} [PP1]^{ecav} P_{O2p}}{K_{RyR\_PP} + [RyR]^{ecav} P_{O2p}} \quad (A.221)$$

$$\frac{dP_{C2p}}{dt} = k_{cp}^{+} P_{O1p} - k_{cp}^{-} P_{C2p} + \frac{f_{RyR} k_{RyR\_PKA} [C]^{ecav} P_{C2}}{K_{RyR\_PKA} + [RyR]^{ecav} P_{C2}} - \frac{k_a^{+} k_{ap}^{-} k_c^{+} k_{cp}^{-}}{k_{ap}^{+} k_a^{-} k_{cp}^{+} k_c^{-}} \frac{f_{RyR} k_{RyR\_PP} [PP1]^{ecav} P_{C2p}}{K_{RyR\_PP} + [RyR]^{ecav} P_{C2p}} \quad (A.222)$$

Na^+-K^+ pump module

Parameter	Definition	Value	Reference
$I_{NaK}^{\max}$	Maximum Na^+-K^+ pump current	4.0 pA/pF	This paper
$K_{m,Nai}^{np}$	Na^+ half-saturation constant for Na^+-K^+ pump current (non-phosphorylated PLM)	18,800 μM	Despa et al. [100]
$K_{m,Nai}^{p}$	Na^+ half-saturation constant for Na^+-K^+ pump current (phosphorylated PLM)	13,600 μM	Despa et al. [100]
$K_{m,Ko}$	K+ half-saturation constant Na^+-K^+ pump current	1,500 μM	Bondarenko et al. [25]
k_{PLM_PKA}	Rate of PLM phosphorylation by PKA	$3.053 \cdot 10^{-3}$ μM^{-1} s^{-1}	This paper
K_{PLM_PKA}	Relative affinity for PLM phosphorylation by PKA	0.0011001	Heijman et al. [8]
k_{PLM_PP}	Rate of PLM dephosphorylation by PP1 and PP2A	$1.8491 \cdot 10^{-2}$ μM^{-1} s^{-1}	This paper
K_{PLM_PP}	Relative affinity for PLM dephosphorylation by PP1 and PP2A	5.7392	Heijman et al. [8]

$$\frac{df_{PLM,p}^{cav}}{dt} = \frac{k_{PLM\_PKA}[C]^{cav}(1 - f_{PLM,p}^{cav})}{K_{PLM\_PKA} + (1 - f_{PLM,p}^{cav})} - \frac{k_{PLM\_PP}[PP]^{cav} f_{PLM,p}^{cav}}{K_{PLM\_PP} + f_{PLM,p}^{cav}} \quad (A.223)$$

$$I_{NaK} = I_{NaK}^{\max} f_{NaK} \frac{1}{1 + (K_{m,Nai} / [Na^+]_i)^3} \frac{[K^+]_0}{[K^+]_0 + K_{m,Ko}} \quad (A.224)$$

$$f_{NaK} = \frac{1}{1 + 0.1245 e^{-0.1VF/RT} + 0.0365 \sigma e^{-VF/RT}} \quad (A.225)$$

$$\sigma = \frac{1}{7}(e^{[Na^+]_0/67300} - 1) \tag{A.226}$$

$$K_{m,Nai} = K_{m,Nai}^{np}(1 - f_{PLM,p}^{cav}) + K_{m,Nai}^{p} f_{PLM,p}^{cav} \tag{A.227}$$

Ultra-rapidly activating delayed rectifier K^+ current module

Parameter	Definition	Value	Reference
G_{Kur}	Specific maximum conductivity for the ultra-rapidly activating delayed rectifier K^+ current (non-phosphorylated)	0.3424 pA/pF	Petkova-Kirova et al. [26]
G_{Kurp}	Specific maximum conductivity for the ultra-rapidly activating delayed rectifier K^+ current (phosphorylated)	0.53307 pA/pF	This paper
k_{IKur_PKA}	Rate of I_{Kur} phosphorylation by PKA	$6.9537 \cdot 10^{-3}$ μM^{-1} s^{-1}	This paper
K_{IKur_PKA}	Relative affinity for I_{Kur} phosphorylation by PKA	0.138115	This paper
k_{IKur_PP}	Rate of I_{Kur} dephosphorylation by PP1	$3.170 \cdot 10^{-2}$ μM^{-1} s^{-1}	This paper
K_{IKur_PP}	Relative affinity for I_{Kur} dephosphorylation by PP1	0.23310	This paper

$$E_K = \frac{RT}{F}\ln\left(\frac{[K^+]_o}{[K^+]_i}\right) \tag{A.228}$$

$$I_{Kur} = (G_{Kur} a_{ur} i_{ur} f_{IKur}^{ecav} + G_{Kurp} a_{urp} i_{urp}(1 - f_{IKur}^{ecav}))(V - E_K) \tag{A.229}$$

$$\frac{df_{IKur}^{ecav}}{dt} = \frac{k_{IKur\_PP}[PP1]^{ecav}(1 - f_{IKur}^{ecav})}{K_{IKur\_PP} + (1 - f_{IKur}^{ecav})} - \frac{k_{IKur\_PKA}[C]^{ecav} f_{IKur}^{ecav}}{K_{IKur\_PKA} + f_{IKur}^{ecav}} \tag{A.230}$$

$$a_{ss} = \frac{1}{1 + e^{-(V+22.5)/7.7}} \tag{A.231}$$

$$i_{ss} = \frac{1}{1 + e^{(V+45.2)/5.7}} \tag{A.232}$$

$$\tau_{aur} = \frac{6.1}{e^{0.0629(V+40.0)} + e^{-0.0629(V+40.0)}} + 2.058 \tag{A.233}$$

$$\tau_{iur} = 1200.0 - \frac{170.0}{1 + e^{(V+45.2)/5.7}} \tag{A.234}$$

$$\frac{da_{ur}}{dt} = \frac{a_{ss} - a_{ur}}{\tau_{aur}} \tag{A.235}$$

$$\frac{di_{ur}}{dt} = \frac{i_{ss} - i_{ur}}{\tau_{iur}} \tag{A.236}$$

$$\frac{da_{urp}}{dt} = \frac{a_{ss} - a_{urp}}{\tau_{aur}} \tag{A.237}$$

$$\frac{di_{urp}}{dt} = \frac{i_{ss} - i_{urp}}{\tau_{iur}} \tag{A.238}$$

Rapidly inactivating transient outward K^+ current module

Parameter	Definition	Value	Reference
$G_{Kto,f}$	Specific maximum conductivity for the rapidly inactivating transient outward K^+ current (non-phosphorylated)	0.3846 pA/pF	Petkova-Kirova et al. [26]
$G_{Kto,fp}$	Specific maximum conductivity for the rapidly inactivating transient outward K^+ current (phosphorylated)	$G_{Kto,f}$	Petkova-Kirova et al. [26]
k_{IKto,f_PKA}	Rate of $I_{Kto,f}$ phosphorylation by PKA	$4.38983 \cdot 10^{-2}$ μM^{-1} s^{-1}	This paper
K_{IKto,f_PKA}	Relative affinity for $I_{Kto,f}$ phosphorylation by PKA	0.27623	This paper
k_{IKto,f_PP}	Rate of $I_{Kto,f}$ dephosphorylation by PP1	$9.09678 \cdot 10^{-2}$ μM^{-1} s^{-1}	This paper
K_{IKto,f_PP}	Relative affinity for $I_{Kto,f}$ dephosphorylation by PP1	0.23310	This paper

$$I_{Kto,f} = (G_{Kto,f} a_{to,f}^3 i_{to,f} (1 - f_{IKto,f}^{ecav}) + G_{Kto,fp} a_{to,fp}^3 i_{to,fp} f_{IKto,f}^{ecav})(V - E_K) \tag{A.239}$$

$$\frac{df_{IKto,f}^{ecav}}{dt} = \frac{k_{IKto,f\_PKA}[C]^{ecav}(1 - f_{IKto,f}^{ecav})}{K_{IKto,f\_PKA} + (1 - f_{IKto,f}^{ecav})} - \frac{k_{IKto,f\_PP}[PP1]^{ecav} f_{IKto,f}^{ecav}}{K_{IKto,f\_PP} + f_{IKto,f}^{ecav}} \tag{A.240}$$

$$\frac{da_{to,f}}{dt} = \alpha_a(1 - a_{to,f}) - \beta_a a_{to,f} \tag{A.241}$$

$$\frac{di_{to,f}}{dt} = \alpha_i(1 - i_{to,f}) - \beta_i i_{to,f} \tag{A.242}$$

$$\frac{da_{to,fp}}{dt} = \alpha_{ap}(1 - a_{to,fp}) - \beta_{ap} a_{to,fp} \tag{A.243}$$

$$\frac{di_{to,fp}}{dt} = \alpha_{ip}(1 - i_{to,fp}) - \beta_{ip} i_{to,fp} \tag{A.244}$$

$$\alpha_a = 0.18064 e^{0.03577(V+33.0)} \tag{A.245}$$

$$\beta_a = 0.3956 e^{-0.06237(V+33.0)} \tag{A.246}$$

$$\alpha_i = \frac{0.000152 e^{-(V+15.5)/7.0}}{0.067083 e^{-(V+35.5)/7.0} + 1} \tag{A.247}$$

$$\beta_i = \frac{0.00095 e^{(V+35.5)/7.0}}{0.051335 e^{(V+35.5)/7.0} + 1} \tag{A.248}$$

$$\alpha_{ap} = 0.18064 e^{0.03577(V+17.0)} \tag{A.249}$$

$$\beta_{ap} = 0.3956 e^{-0.06237(V+17.0)} \tag{A.250}$$

$$\alpha_{ip} = \frac{0.000152 e^{-(V+7.5)/7.0}}{0.067083 e^{-(V+27.5)/7.0} + 1} \tag{A.251}$$

$$\beta_{ip} = \frac{0.00095 e^{(V+27.5)/7.0}}{0.051335 e^{(V+27.5)/7.0} + 1} \tag{A.252}$$

Time-independent K^+ current module

$$\alpha_{\mathrm{K1}} = \frac{1.02}{1 + \exp(0.2385(V - E_K - 59.215))} \tag{A.253}$$

$$\beta_{K1} = \frac{0.8\exp(0.08032(V - E_K + 5.476)) + \exp(0.06175(V - E_K - 594.31))}{1 + \exp(-0.5143(V - E_K + 4.753))} \quad (A.254)$$

$$I_{K1} = 0.27\sqrt{\frac{[K^+]_o}{5400}}\frac{\alpha_{K1}}{\alpha_{K1} + \beta_{K1}}(V - E_K) \quad (A.255)$$

Phospholamban module

Paramet er	Definition	Value	Reference
$K_{m,up}^{np}$	Half-saturation constant for SR Ca^{2+}-ATPase pump (non-phosphorylated)	0.41 μM	This paper
$K_{m,up}^{p}$	Half-saturation constant for SR Ca^{2+}-ATPase pump (phosphorylated)	0.31 μM	This paper
v_3	SR Ca^{2+}-ATPase maximum pump rate	306.0 μM s^{-1}	This paper
k_{PLB_PKA}	Rate of PLB phosphorylation by PKA	0.108917 μM^{-1} s^{-1}	This paper
K_{PLB_PKA}	Relative affinity for PLB phosphorylation by PKA	$4.90970 \cdot 10^{-4}$	Heijman et al. [8]
k_{PLB_PP1}	Rate of PLB dephosphorylation by PP1	$4.41956 \cdot 10^{-2}$ μM^{-1} s^{-1}	This paper
K_{PLB_PP1}	Relative affinity for PLB dephosphorylation by PP1	$1.69376 \cdot 10^{-2}$	This paper

$$K_{m,up} = K_{m,up}^{np}(1 - f_{PLB,p}^{cyt}) + K_{m,up}^{p} f_{PLB,p}^{cyt} \quad (A.256)$$

$$\frac{df_{PLB,p}^{cyt}}{dt} = \frac{k_{PLB\_PKA} \cdot [C]^{cyt} \cdot (1 - f_{PLB,p}^{cyt})}{K_{PLB\_PKA} + (1 - f_{PLB,p}^{cyt})} - \frac{k_{PLB\_PP1} \cdot [PP1]_f^{cyt} \cdot f_{PLB,p}^{cyt}}{K_{PLB\_PP1} + f_{PLB,p}^{cyt}} \quad (A.257)$$

Troponin I module

Parameter	Definition	Value	Reference
$[LTRPN]_{tot}$	Total cytosolic troponin low-affinity site concentration	70.0 μM	Bondarenko et al. [25]
$[HTRPN]_{tot}$	Total cytosolic troponin high-affinity site concentration	140.0 μM	Bondarenko et al. [25]
k_{htrpn}^{+}	Ca^{2+} on rate constant for troponin high-affinity sites	2.37 μM s^{-1}	Bondarenko et al. [25]

k_{htrpn}^{-}	Ca^{2+} off rate constant for troponin high-affinity sites	0.032 s^{-1}	Bondarenko et al. [25]
k_{ltrpn}^{+}	Ca^{2+} on rate constant for troponin low-affinity sites	32.7 $\mu M\ s^{-1}$	Bondarenko et al. [25]
$k_{ltrpn,np}^{-}$	Ca^{2+} off rate constant for troponin low-affinity sites (non-phosphorylated)	19.6 s^{-1}	Bondarenko et al. [25]
$k_{ltrpn,p}^{-}$	Ca^{2+} off rate constant for troponin low-affinity sites (phosphorylated)	29.4 s^{-1}	This paper
k_{TnI_PKA}	Rate of TnI phosphorylation by PKA	0.0247254 $\mu M^{-1}\ s^{-1}$	This paper
K_{TnI_PKA}	Relative affinity for TnI phosphorylation by PKA	$2.71430 \cdot 10^{-5}$	Heijman et al. [8]
k_{TnI_PP2A}	Rate of TnI dephosphorylation by PP2A	0.0865898 $\mu M^{-1}\ s^{-1}$	This paper
K_{TnI_PP2A}	Relative affinity for TnI dephosphorylation by PP2A	0.801420	This paper

$$k_{ltrpn}^{-} = k_{ltrpn,np}^{-}(1 - f_{TnI,p}^{cyt}) + k_{ltrpn,p}^{-} f_{TnI,p}^{cyt} \tag{A.258}$$

$$\frac{df_{TnI,p}^{cyt}}{dt} = \frac{k_{TnI\_PKA} \cdot [C]^{cyt} \cdot (1 - f_{TnI,p}^{cyt})}{K_{TnI\_PKA} + (1 - f_{TnI,p}^{cyt})} - \frac{k_{TnI\_PP2A} \cdot [PP2A]^{cyt} \cdot f_{TnI,p}^{cyt}}{K_{TnI\_PP2A} + f_{TnI,p}^{cyt}} \tag{A.259}$$

$$\frac{d[LTRPNCa]}{dt} = k_{ltrpn}^{+}[Ca^{2+}]_i([LTRPN]_{tot} - [LTRPNCa]) - k_{ltrpn}^{-}[LTRPNCa] \tag{A.260}$$

$$\frac{d[HTRPNCa]}{dt} = k_{htrpn}^{+}[Ca^{2+}]_i([HTRPN]_{tot} - [HTRPNCa]) - k_{htrpn}^{-}[HTRPNCa] \tag{A.261}$$

ELECTROPHYSIOLOGICAL PART. UNAFFECTED BY PKA

Membrane potential

$$\frac{dV}{dt} = -\frac{1}{C_m}(I_{CaL} + I_{p(Ca)} + I_{NaCa} + I_{Cab} + I_{Na} + I_{Nab} + I_{NaK} + I_{Kto,f} + I_{K1} + I_{Kur} + I_{Kss} + I_{Kr} + I_{Cl,Ca} - I_{stim}) \quad \text{(A.262)}$$

Calcium dynamics: Calcium concentrations

$$\frac{d[Ca^{2+}]_i}{dt} = B_i \left\{ J_{leak} + J_{xfer} - J_{up} - J_{trpn} - (I_{Cab} - 2I_{NaCa} + I_{p(Ca)} + I_{CaL}^{cav}) \frac{A_{cap}C_m}{2V^{cyt}F} \right\} \quad \text{(A.263)}$$

$$\frac{d[Ca^{2+}]_{ss}}{dt} = B_{ss} \left\{ J_{rel} \frac{V_{JSR}}{V_{ss}} - J_{xfer} \frac{V^{cyt}}{V_{ss}} - I_{CaL}^{ecav} \frac{A_{cap}C_m}{2V_{ss}F} \right\} \quad \text{(A.264)}$$

$$\frac{d[Ca^{2+}]_{JSR}}{dt} = B_{JSR} \left\{ J_{tr} - J_{rel} \right\} \quad \text{(A.265)}$$

$$\frac{d[Ca^{2+}]_{NSR}}{dt} = \left\{ J_{up} - J_{leak} \right\} \frac{V^{cyt}}{V_{NSR}} - J_{tr} \frac{V_{JSR}}{V_{NSR}} \quad \text{(A.266)}$$

$$B_i = \left\{ 1 + \frac{[CMDN]_{tot} K_m^{CMDN}}{(K_m^{CMDN} + [Ca^{2+}]_i)^2} \right\}^{-1} \quad \text{(A.267)}$$

$$B_{ss} = \left\{ 1 + \frac{[CMDN]_{tot} K_m^{CMDN}}{(K_m^{CMDN} + [Ca^{2+}]_{ss})^2} \right\}^{-1} \quad \text{(A.268)}$$

$$B_{JSR} = \left\{ 1 + \frac{[CSQN]_{tot} K_m^{CSQN}}{(K_m^{CSQN} + [Ca^{2+}]_{JSR})^2} \right\}^{-1} \quad \text{(A.269)}$$

Calcium dynamics: Calcium fluxes

$$J_{rel} = v_1(P_{O1} + P_{O2} + P_{O1p} + P_{O2p})([Ca^{2+}]_{JSR} - [Ca^{2+}]_{ss} P_{RyR}) \quad \text{(A.270)}$$

$$J_{tr} = \frac{[Ca^{2+}]_{NSR} - [Ca^{2+}]_{JSR}}{\tau_{tr}} \quad \text{(A.271)}$$

$$J_{xfer} = \frac{[Ca^{2+}]_{ss} - [Ca^{2+}]_i}{\tau_{xfer}} \tag{A.272}$$

$$J_{leak} = v_2([Ca^{2+}]_{NSR} - [Ca^{2+}]_i) \tag{A.273}$$

$$J_{up} = v_3 \frac{[Ca^{2+}]_i^2}{K_{m,up}^2 + [Ca^{2+}]_i^2} \tag{A.274}$$

$$\begin{aligned} J_{trpn} = {} & k_{htrpn}^{+}[Ca^{2+}]_i([HTRPN]_{tot} - [HTRPNCa]) - k_{htrpn}^{-}[HTRPNCa] \\ & + k_{ltrpn}^{+}[Ca^{2+}]_i([LTRPN]_{tot} - [LTRPNCa]) - k_{ltrpn}^{-}[LTRPNCa] \end{aligned} \tag{A.275}$$

$$\frac{dP_{RyR}}{dt} = -0.04P_{RyR} - 0.1\frac{I_{CaL}^{ecav}}{I_{CaL,\max}} e^{-\frac{(V+5.0)^2}{648.0}} \tag{A.276}$$

Calcium pump current

$$I_{p(Ca)} = I_{p(Ca)}^{\max} \frac{[Ca^{2+}]_i^2}{K_{m,p(Ca)}^2 + [Ca^{2+}]_i^2} \tag{A.277}$$

Na^+/Ca^{2+} exchanger current

$$\begin{aligned} I_{NaCa} = {} & k_{NaCa} \frac{1}{K_{m,Na}^3 + [Na^+]_o^3} \frac{1}{K_{m,Ca} + [Ca^{2+}]_o} \frac{1}{1 + k_{sat} e^{(\eta-1)VF/RT}} \\ & \times (e^{\eta VF/RT}[Na^+]_i^3[Ca^{2+}]_o - 2.0e^{(\eta-1)VF/RT}[Na^+]_o^3[Ca^{2+}]_i) \end{aligned} \tag{A.278}$$

Calcium background current

$$I_{Cab} = G_{Cab}(V - E_{CaN}) \tag{A.279}$$

$$E_{CaN} = \frac{RT}{2F}\ln\left(\frac{[Ca^{2+}]_o}{[Ca^{2+}]_i}\right) \tag{A.280}$$

Sodium dynamics: Sodium concentration

$$\frac{d[Na^{+}]_i}{dt} = -(I_{Na} + +I_{Nab} + 3I_{NaCa} + 3I_{NaK})\frac{A_{cap}C_m}{V^{cyt}F} \tag{A.281}$$

Sodium background current

$$I_{Nab} = G_{Nab}(V - E_{Na}) \tag{A.282}$$

Potassium dynamics: Potassium concentration

$$\frac{d[K^{+}]_i}{dt} = -(I_{Kto,f} + I_{Kto,s} + I_{Kur} + I_{Kss} + I_{K1} + I_{Kr} + I_{Ks} - 2I_{NaK})\frac{A_{cap}C_m}{V^{cyt}F} \tag{A.283}$$

Non-inactivating steady-state K^{+} current

$$I_{Kss} = G_{Kss}a_{Kss}(V - E_K) \tag{A.284}$$

$$\frac{da_{Kss}}{dt} = \frac{a_{ss} - a_{Kss}}{\tau_{Kss}} \tag{A.285}$$

$$\tau_{Kss} = \frac{1235.5}{e^{0.0862(V+40.0)} + e^{-0.0862(V+40.0)}} + 13.17 \tag{A.286}$$

Rapid delayed rectifier K^{+} current

$$I_{Kr} = G_{Kr}O_{Kr}(V - E_{Kr}) \tag{A.287}$$

$$E_{Kr} = \frac{RT}{F}\ln\left(\frac{0.98[K^{+}]_o + 0.02[Na^{+}]_o}{0.98[K^{+}]_i + 0.02[Na^{+}]_i}\right) \tag{A.288}$$

$$C_{Kr0} = 1 - (C_{Kr1} + C_{Kr2} + O_{Kr} + I_{Kr1}) \tag{A.289}$$

$$\frac{dC_{Kr1}}{dt} = \alpha_{a0} C_{Kr0} - \beta_{a0} C_{Kr1} + k_b C_{Kr2} - k_f C_{Kr1} \tag{A.290}$$

$$\frac{dC_{Kr2}}{dt} = k_f C_{Kr1} - k_b C_{Kr2} + \beta_{a1} O_{Kr} - \alpha_{a1} C_{Kr2} \tag{A.291}$$

$$\frac{dO_{Kr}}{dt} = \alpha_{a1} C_{Kr2} - \beta_{a1} O_{Kr} + \beta_{ir} I_{Kr1} - \alpha_{ir} O_{Kr} \tag{A.292}$$

$$\frac{dI_{Kr1}}{dt} = \alpha_{ir} O_{Kr} + \beta_{ir} I_{Kr1} \tag{A.293}$$

$$\alpha_{a0} = 0.022348 e^{0.01176V} \tag{A.294}$$

$$\beta_{a0} = 0.047002 e^{-0.0631V} \tag{A.295}$$

$$\alpha_{a1} = 0.013733 e^{0.038198V} \tag{A.296}$$

$$\beta_{a1} = 0.0000689 e^{-0.04178V} \tag{A.297}$$

$$\alpha_{ir} = 0.090821 e^{0.023391(V+5.0)} \tag{A.298}$$

$$\beta_{ir} = 0.006497 e^{-0.03268(V+5.0)} \tag{A.299}$$

Ca^{2+}-activated Cl^{-} current

$$I_{Cl,Ca} = G_{Cl,Ca} O_{Cl,Ca} \frac{[Ca^{2+}]_i}{[Ca^{2+}]_i + K_{m,Cl}} (V - E_{Cl}) \tag{A.300}$$

$$O_{Cl,Ca} = \frac{0.2}{1 + e^{-(V-46.7)/7.8}} \tag{A.301}$$

Extracellular ion concentrations

Parameter	Definition	Value	Reference
$[K^+]_o$	Extracellular K^+ concentration	5,400 μM	Bondarenko et al. [25]
$[Na^+]_o$	Extracellular Na^+ concentration	140,000 μM	Bondarenko et al. [25]
$[Ca^{2+}]_o$	Extracellular Ca^{2+} concentration	1,800 μM	Bondarenko et al. [25]

Sarcoplasmic reticulum parameters

Parameter	Definition	Value	Reference
v_2	Ca^{2+} leak rate constant from the NSR	$1.74 \cdot 10^{-2}$ s^{-1}	Bondarenko et al. [25]
τ_{tr}	Time constant for transfer from NSR to JSR	0.02 s	Bondarenko et al. [25]
τ_{xfer}	Time constant for transfer from subspace to cytosol	0.008 s	Bondarenko et al. [25]

Calmodulin and calsequestrin parameters

Parameter	Definition	Value	Reference
$[CMDN]_{tot}$	Total cytosolic calmodulin concentration	50.0 μM	Bondarenko et al. [25]
$[CSQN]_{tot}$	Total JSR calsequestrin concentration	15,000.0 μM	Bondarenko et al. [25]
K_m^{CMDN}	Ca^{2+} half-saturation constant for calmodulin	0.238 μM	Bondarenko et al. [25]
K_m^{CSQN}	Ca^{2+} half-saturation constant for calsequestrin	800.0 μM	Bondarenko et al. [25]

Membrane current parameters

Parameter	Definition	Value	Reference
C_m	Specific membrane capacitance	1.0 $\mu F/cm^2$	Bondarenko et al. [25]
F	Faraday constant	96.5 C/mmol	Bondarenko et al. [25]
T	Absolute temperature	298 K	Bondarenko et al. [25]
R	Ideal gas constant	8.314 J mol^{-1} K^{-1}	Bondarenko et al. [25]

k_{NaCa}	Scaling factor for Na^+/Ca^{2+} exchanger	275 pA/pF	This paper
$K_{m,Na}$	Na^+ half-saturation constant for Na^+/Ca^{2+} exchanger	87,500 μM	Bondarenko et al. [25]
$K_{m,Ca}$	Ca^{2+} half-saturation constant for Na^+/Ca^{2+} exchanger	1,380 μM	Bondarenko et al. [25]
k_{sat}	Na^+/Ca^{2+} exchanger saturation factor at very negative potentials	0.27	This paper
η	Controls voltage dependence of Na^+/Ca^{2+} exchanger	0.35	Bondarenko et al. [25]
$I^{\max}_{p(Ca)}$	Maximum sarcolemmal Ca^{2+} pump current	0.051	This paper
$K_{m,p(Ca)}$	Ca^{2+} half-saturation constant for sarcolemmal Ca^{2+} pump current	0.5 μM	Bondarenko et al. [25]
G_{Cab}	Maximum background Ca^{2+} current conductance	0.000284 mS/μF	This paper
G_{Nab}	Maximum background Na^+ current conductance	0.0063 mS/μF	This paper
$G_{Kto,s}$	Specific maximum conductivity for the slowly inactivating transient outward K^+ current	0.0 mS/μF	Bondarenko et al. [25]
G_{Kss}	Specific maximum conductivity for the noninactivating steady-state K^+ current	0.0611 mS/μF	Petkova-Kirova et al. [26]
G_{Ks}	Specific maximum conductivity for the slow delayed rectifier K^+ current	0.00575 mS/μF	Bondarenko et al. [25]
G_{Kr}	Specific maximum conductivity for the rapid delayed rectifier K^+ current	0.078 mS/μF	Bondarenko et al. [25]
k_f	Rate constant for the rapid delayed rectifier K^+ current	23.761 s^{-1}	Bondarenko et al. [25]
k_b	Rate constant for the rapid delayed rectifier K^+ current	36.778 s^{-1}	Bondarenko et al. [25]
$G_{Cl,Ca}$	Specific maximum conductivity for the Ca^{2+}-activated Cl^- current	10.0 mS/μF	Bondarenko et al. [25]
$K_{m,Cl}$	Half-saturation constant for the Ca^{2+}-activated Cl^- current	10.0 μM	Bondarenko et al. [25]
E_{Cl}	Reversal potential for the Ca^{2+}-activated Cl^- current	−40 mV	Bondarenko et al. [25]

INITIAL CONDITIONS

Variable	Definition	Initial Value
V	Membrane potential	–78.2787 mV
$[Ca^{2+}]_i$	Myoplasmic Ca^{2+} concentration	0.100157 μM
$[Ca^{2+}]_{ss}$	Subspace SR Ca^{2+} concentration	0.100157 μM
$[Ca^{2+}]_{JSR}$	Junctional SR Ca^{2+} concentration	1081.23 μM
$[Ca^{2+}]_{NSR}$	Network SR Ca^{2+} concentration	1081.23 μM
[LTRPNCa]	Concentration Ca^{2+} bound by low-affinity troponin binding sites	8.66981 μM
[HTRPNCa]	Concentration Ca^{2+} bound by high-affinity troponin binding sites	123.369 μM
O^{cav}	L-type Ca^{2+} channel conducting state (non-phosphorylated, caveolae)	$0.320206 \cdot 10^{-11}$
C_1^{cav}	L-type Ca^{2+} channel closed state (non-phosphorylated, caveolae)	0.973685
C_2^{cav}	L-type Ca^{2+} channel closed state (non-phosphorylated, caveolae)	$0.524483 \cdot 10^{-2}$
C_3^{cav}	L-type Ca^{2+} channel closed state (non-phosphorylated, caveolae)	$0.105944 \cdot 10^{-4}$
C_4^{cav}	L-type Ca^{2+} channel closed state (non-phosphorylated, caveolae)	$0.951124 \cdot 10^{-8}$
C_P^{cav}	L-type Ca^{2+} channel closed state (non-phosphorylated, caveolae)	$0.320207 \cdot 10^{-11}$
I_1^{cav}	L-type Ca^{2+} channel inactivated state (non-phosphorylated, caveolae)	$0.308577 \cdot 10^{-11}$
I_2^{cav}	L-type Ca^{2+} channel inactivated state (non-phosphorylated, caveolae)	$0.217536 \cdot 10^{-7}$
I_3^{cav}	L-type Ca^{2+} channel inactivated state (non-phosphorylated, caveolae)	$0.209641 \cdot 10^{-7}$
O_p^{cav}	L-type Ca^{2+} channel conducting state (phosphorylated, caveolae)	$0.562222 \cdot 10^{-10}$
C_{1p}^{cav}	L-type Ca^{2+} channel closed state (phosphorylated, caveolae)	$0.206347 \cdot 10^{-1}$
C_{2p}^{cav}	L-type Ca^{2+} channel closed state (phosphorylated, caveolae)	$0.421668 \cdot 10^{-3}$
C_{3p}^{cav}	L-type Ca^{2+} channel closed state (phosphorylated, caveolae)	$0.323128 \cdot 10^{-5}$
C_{4p}^{cav}	L-type Ca^{2+} channel closed state (phosphorylated, caveolae)	$0.110051 \cdot 10^{-7}$
C_{Pp}^{cav}	L-type Ca^{2+} channel closed state (phosphorylated, caveolae)	$0.140555 \cdot 10^{-10}$
I_{1p}^{cav}	L-type Ca^{2+} channel inactivated state (phosphorylated, caveolae)	$0.541817 \cdot 10^{-10}$
I_{2p}^{cav}	L-type Ca^{2+} channel inactivated state (phosphorylated, caveolae)	$0.100683 \cdot 10^{-6}$
I_{3p}^{cav}	L-type Ca^{2+} channel inactivated state (phosphorylated, caveolae)	$0.970287 \cdot 10^{-7}$
O^{ecav}	L-type Ca^{2+} channel conducting state (non-phosphorylated, extracaveolae)	$0.286851 \cdot 10^{-11}$
C_1^{ecav}	L-type Ca^{2+} channel closed state (non-phosphorylated, extracaveolae)	0.872261
C_2^{ecav}	L-type Ca^{2+} channel closed state (non-phosphorylated, extracaveolae)	$0.469850 \cdot 10^{-2}$
C_3^{ecav}	L-type Ca^{2+} channel closed state (non-phosphorylated, extracaveolae)	$0.949082 \cdot 10^{-5}$

C_4^{ecav}	L-type Ca^{2+} channel closed state (non-phosphorylated, extracaveolae)	$0.852050 \cdot 10^{-8}$
C_P^{ecav}	L-type Ca^{2+} channel closed state (non-phosphorylated, extracaveolae)	$0.286852 \cdot 10^{-11}$
I_1^{ecav}	L-type Ca^{2+} channel inactivated state (non-phosphorylated, extracaveolae)	$0.276420 \cdot 10^{-11}$
I_2^{ecav}	L-type Ca^{2+} channel inactivated state (non-phosphorylated, extracaveolae)	$0.194870 \cdot 10^{-7}$
I_3^{ecav}	L-type Ca^{2+} channel inactivated state (non-phosphorylated, extracaveolae)	$0.187798 \cdot 10^{-7}$
O_p^{ecav}	L-type Ca^{2+} channel conducting state (phosphorylated, extracaveolae)	$0.328449 \cdot 10^{-9}$
C_{1p}^{ecav}	L-type Ca^{2+} channel closed state (phosphorylated, extracaveolae)	0.120548
C_{2p}^{ecav}	L-type Ca^{2+} channel closed state (phosphorylated, extracaveolae)	$0.246338 \cdot 10^{-2}$
C_{3p}^{ecav}	L-type Ca^{2+} channel closed state (phosphorylated, extracaveolae)	$0.188771 \cdot 10^{-4}$
C_{4p}^{ecav}	L-type Ca^{2+} channel closed state (phosphorylated, extracaveolae)	$0.642918 \cdot 10^{-7}$
C_{Pp}^{ecav}	L-type Ca^{2+} channel closed state (phosphorylated, extracaveolae)	$0.821123 \cdot 10^{-10}$
I_{1p}^{ecav}	L-type Ca^{2+} channel inactivated state (phosphorylated, extracaveolae)	$0.316528 \cdot 10^{-9}$
I_{2p}^{ecav}	L-type Ca^{2+} channel inactivated state (phosphorylated, extracaveolae)	$0.588189 \cdot 10^{-6}$
I_{3p}^{ecav}	L-type Ca^{2+} channel inactivated state (phosphorylated, extracaveolae)	$0.566840 \cdot 10^{-6}$
C_{Na3}	Fast Na^+ channel closed state (non-phosphorylated)	0.436222
C_{Na2}	Fast Na^+ channel closed state (non-phosphorylated)	$0.132248 \cdot 10^{-1}$
C_{Na1}	Fast Na^+ channel closed state (non-phosphorylated)	$0.161178 \cdot 10^{-3}$
O_{Na}	Fast Na^+ channel open state (non-phosphorylated)	$0.367777 \cdot 10^{-6}$
IF_{Na}	Fast Na^+ channel inactivated state (non-phosphorylated)	$0.153271 \cdot 10^{-3}$
$I1_{Na}$	Fast Na^+ channel inactivated state (non-phosphorylated)	$0.146044 \cdot 10^{-4}$
$I2_{Na}$	Fast Na^+ channel inactivated state (non-phosphorylated)	$0.545874 \cdot 10^{-7}$
IC_{Na2}	Fast Na^+ channel inactivated state (non-phosphorylated)	$0.125760 \cdot 10^{-1}$
IC_{Na3}	Fast Na^+ channel inactivated state (non-phosphorylated)	0.414822
C_{Na3p}	Fast Na^+ channel closed state (phosphorylated)	$0.610809 \cdot 10^{-1}$
C_{Na2p}	Fast Na^+ channel closed state (phosphorylated)	$0.185179 \cdot 10^{-2}$
C_{Na1p}	Fast Na^+ channel closed state (phosphorylated)	$0.225696 \cdot 10^{-4}$
O_{Nap}	Fast Na^+ channel open state (phosphorylated)	$0.515006 \cdot 10^{-7}$
IF_{Nap}	Fast Na^+ channel inactivated state (phosphorylated)	$0.214630 \cdot 10^{-4}$
$I1_{Nap}$	Fast Na^+ channel inactivated state (phosphorylated)	$0.217162 \cdot 10^{-5}$
$I2_{Nap}$	Fast Na^+ channel inactivated state (phosphorylated)	$0.301835 \cdot 10^{-7}$

IC_{Na2p}	Fast Na^+ channel inactivated state (phosphorylated)	$0.176099 \cdot 10^{-2}$
IC_{Na3p}	Fast Na^+ channel inactivated state (phosphorylated)	$0.580859 \cdot 10^{-1}$
P_{C1}	RyR channel closed state (non-phosphorylated)	0.996216
P_{C2}	RyR channel closed state (non-phosphorylated)	$0.961561 \cdot 10^{-4}$
P_{O1}	RyR channel open state (non-phosphorylated)	$0.854737 \cdot 10^{-5}$
P_{O2}	RyR channel open state (non-phosphorylated)	$0.360412 \cdot 10^{-10}$
P_{C1p}	RyR channel closed state (phosphorylated)	$0.367832 \cdot 10^{-2}$
P_{C2p}	RyR channel closed state (phosphorylated)	$0.986431 \cdot 10^{-6}$
P_{O1p}	RyR channel open state (phosphorylated)	$0.526065 \cdot 10^{-7}$
P_{O2p}	RyR channel open state (phosphorylated)	$0.369705 \cdot 10^{-12}$
$[Na^+]_i$	Myoplasmic Na^+ concentration	10,508.5 μM
$[K^+]_i$	Myoplasmic K^+ concentration	145,400 μM
$f_{PLM,p}^{cav}$	Fraction of phosphorylated phospholemman	0.225905
f_{IKur}^{ecav}	Fraction of non-phosphorylated I_{Kur}	0.908852
a_{ur}	Activation gate of non-phosphorylated I_{Kur}	$0.713943 \cdot 10^{-3}$
i_{ur}	Inactivation gate of non-phosphorylated I_{Kur}	0.996991
a_{urp}	Activation gate of phosphorylated I_{Kur}	$0.713943 \cdot 10^{-3}$
i_{urp}	Inactivation gate of phosphorylated I_{Kur}	0.996991
$f_{IKto,f}^{ecav}$	Fraction of phosphorylated $I_{Kto,f}$	0.252661
$a_{to,f}$	Activation gate of non-phosphorylated $I_{Kto,f}$	$0.533799 \cdot 10^{-2}$
$i_{to,f}$	Inactivation gate of non-phosphorylated $I_{Kto,f}$	0.999945
$a_{to,fp}$	Activation gate of phosphorylated $I_{Kto,f}$	$0.111499 \cdot 10^{-2}$
$i_{to,fp}$	Inactivation gate of phosphorylated $I_{Kto,f}$	0.999983
$f_{PLB,p}^{cyt}$	Fraction of phosphorylated phospholamban	0.186637
$f_{TnI,p}^{cyt}$	Fraction of phosphorylated troponin I	0.364102
P_{RyR}	RyR modulation factor	$0.254152 \cdot 10^{-11}$
a_{Kss}	Activation gate of I_{Kss}	$0.713943 \cdot 10^{-3}$
C_{K0}	mERG channel closed state	0.997365
C_{K1}	mERG channel closed state	$0.135218 \cdot 10^{-2}$
C_{K2}	mERG channel closed state	$0.873596 \cdot 10^{-3}$
O_K	mERG channel open state	$0.332600 \cdot 10^{-3}$
I_K	mERG channel inactivated state	$0.763767 \cdot 10^{-4}$
$[R_{\beta1}]_{PKA,tot}^{cav}$	Concentration of total β_1-ARs phosphorylated by PKA (caveolae)	$0.799452 \cdot 10^{-3}$ μM

$[R_{\beta 1}]^{cav}_{GRK2,tot}$	Concentration of total β_1-ARs phosphorylated by GRK2 (caveolae)	$0.626341 \cdot 10^{-27}$ μM
$[G_s]^{cav}_{\alpha,GTP}$	Concentration of active $G_{s\alpha}$ subunit (caveolae)	$0.132189 \cdot 10^{-2}$ μM
$[G_s]^{cav}_{\beta\gamma}$	Concentration of $G_{s\beta\gamma}$ subunit (caveolae)	$0.180824 \cdot 10^{-2}$ μM
$[G_s]^{cav}_{\alpha,GDP}$	Concentration of inactive $G_{s\alpha}$ subunit (caveolae)	$0.487356 \cdot 10^{-3}$ μM
$[R_{\beta 1}]^{ecav}_{PKA,tot}$	Concentration of total β_1-ARs phosphorylated by PKA (extracaveolae)	$0.478002 \cdot 10^{-1}$ μM
$[R_{\beta 1}]^{ecav}_{GRK2,tot}$	Concentration of total β_1-ARs phosphorylated by GRK2 (extracaveolae)	$0.626341 \cdot 10^{-27}$ μM
$[G_s]^{ecav}_{\alpha,GTP}$	Concentration of active $G_{s\alpha}$ subunit (extracaveolae)	$0.230801 \cdot 10^{-1}$ μM
$[G_s]^{ecav}_{\beta\gamma}$	Concentration of $G_{s\beta\gamma}$ subunit (extracaveolae)	$0.237276 \cdot 10^{-1}$ μM
$[G_s]^{ecav}_{\alpha,GDP}$	Concentration of inactive $G_{s\alpha}$ subunit (extracaveolae)	$0.648475 \cdot 10^{-3}$ μM
$[R_{\beta 1}]^{cyt}_{PKA,tot}$	Concentration of total β_1-ARs phosphorylated by PKA (cytosol)	$0.155949 \cdot 10^{-2}$ μM
$[R_{\beta 1}]^{cyt}_{GRK2,tot}$	Concentration of total β_1-ARs phosphorylated by GRK2 (cytosol)	$0.626341 \cdot 10^{-27}$ μM
$[G_s]^{cyt}_{\alpha,GTP}$	Concentration of active $G_{s\alpha}$ subunit (cytosol)	$0.331511 \cdot 10^{-3}$ μM
$[G_s]^{cyt}_{\beta\gamma}$	Concentration of $G_{s\beta\gamma}$ subunit (cytosol)	$0.663570 \cdot 10^{-3}$ μM
$[G_s]^{cyt}_{\alpha,GDP}$	Concentration of inactive $G_{s\alpha}$ subunit (cytosol)	$0.333058 \cdot 10^{-3}$ μM
$[cAMP]^{cav}_{AC56}$	cAMP concentration produced by AC5/6 (caveolae)	0.000000 μM
$[cAMP]^{ecav}_{AC47}$	cAMP concentration produced by AC4/7 (extracaveolae)	0.000000 μM
$[cAMP]^{cyt}_{AC56}$	cAMP concentration produced by AC5/6 (cytosol)	0.000000 μM
$[cAMP]^{cyt}_{AC47}$	cAMP concentration produced by AC4/7 (cytosol)	0.000000 μM
$[PDE3]^{cav}_{p}$	Concentration of phosphorylated PDE3 (caveolae)	$0.125103 \cdot 10^{-1}$ μM
$[PDE4]^{cav}_{p}$	Concentration of phosphorylated PDE4 (caveolae)	$0.580798 \cdot 10^{-2}$ μM
$[cAMP]^{cav}_{PDE2}$	cAMP concentration degraded by PDE2 (caveolae)	0.000000 μM
$[cAMP]^{cav}_{PDE3}$	cAMP concentration degraded by PDE3 (caveolae)	0.000000 μM
$[cAMP]^{cav}_{PDE4}$	cAMP concentration degraded by PDE4 (caveolae)	0.000000 μM
$[PDE4]^{ecav}_{p}$	Concentration of phosphorylated PDE4 (extracaveolae)	$0.158226 \cdot 10^{-1}$ μM
$[cAMP]^{ecav}_{PDE2}$	cAMP concentration degraded by PDE2 (extracaveolae)	0.000000 μM
$[cAMP]^{ecav}_{PDE4}$	cAMP concentration degraded by PDE4 (extracaveolae)	0.000000 μM
$[PDE3]^{cyt}_{p}$	Concentration of phosphorylated PDE3 (cytosol)	$0.120998 \cdot 10^{-2}$ μM
$[PDE4]^{cyt}_{p}$	Concentration of phosphorylated PDE4 (cytosol)	$0.373102 \cdot 10^{-2}$ μM
$[cAMP]^{cyt}_{PDE2}$	cAMP concentration degraded by PDE2 (cytosol)	0.000000 μM

$[cAMP]_{PDE3}^{cyt}$	cAMP concentration degraded by PDE3 (cytosol)	0.000000 μM
$[cAMP]_{PDE4}^{cyt}$	cAMP concentration degraded by PDE4 (cytosol)	0.000000 μM
$[cAMP]_{PKA}^{cav}$	cAMP concentration change due to binding to PKA (caveolae)	7.92317 μM
$[ARC]^{cav}$	Concentration of PKA RC dimer with 1 cAMP molecule bound (caveolae)	0.299288 μM
$[A_2RC]^{cav}$	Concentration of PKA RC dimer with 2 cAMP molecules bound (caveolae)	$0.303358 \cdot 10^{-1}$ μM
$[A_2R]^{cav}$	Concentration of PKA R subunit with 2 cAMP molecules bound (caveolae)	0.858440 μM
$[C]^{cav}$	Concentration of free PKA catalytic subunit (caveolae)	$0.459397 \cdot 10^{-1}$ μM
$[PKIC]^{cav}$	Concentration of PKI inactivated PKA catalytic subunit (caveolae)	0.823499 μM
$[cAMP]_{PKA}^{ecav}$	cAMP concentration change due to binding to PKA (extracaveolae)	6.74029 μM
$[ARC]^{ecav}$	Concentration of PKA RC dimer with 1 cAMP molecule bound (extracaveolae)	0.653988 μM
$[A_2RC]^{ecav}$	Concentration of PKA RC dimer with 2 cAMP molecules bound (extracaveolae)	0.132861 μM
$[A_2R]^{ecav}$	Concentration of PKA R subunit with 2 cAMP molecules bound (extracaveolae)	1.17000 μM
$[C]^{ecav}$	Concentration of free PKA catalytic subunit (extracaveolae)	0.147623 μM
$[PKIC]^{ecav}$	Concentration of PKI inactivated PKA catalytic subunit (extracaveolae)	1.03338 μM
$[cAMP]_{PKA}^{cyt}$	cAMP concentration change due to binding to PKA (cytosol)	9.32461 μM
$[ARC]^{cyt}$	Concentration of PKA RC dimer with 1 cAMP molecule bound (cytosol)	$0.996350 \cdot 10^{-1}$ μM
$[A_2RC]^{cyt}$	Concentration of PKA RC dimer with 2 cAMP molecules bound (cytosol)	$0.140099 \cdot 10^{-1}$ μM
$[A_2R]^{cyt}$	Concentration of PKA R subunit with 2 cAMP molecules bound (cytosol)	0.273868 μM
$[C]^{cyt}$	Concentration of free PKA catalytic subunit (cytosol)	$0.665022 \cdot 10^{-1}$ μM
$[PKIC]^{cyt}$	Concentration of PKI inactivated PKA catalytic subunit (cytosol)	0.218365 μM
$[Inhib1]_{p,tot}^{cyt}$	Concentration of total phosphorylated PP1 inhibitor 1 (cytosol)	$0.213571 \cdot 10^{-1}$ μM
$[cAMP]^{cav}$	Concentration of cAMP in caveolae	0.253399 μM
$[cAMP]^{ecav}$	Concentration of cAMP in extracaveolae	0.507889 μM
$[cAMP]^{cyt}$	Concentration of cAMP in cytosol	0.407775 μM
